# A recurrent *PJA1* variant in trigonocephaly and neurodevelopmental disorders

**DOI:** 10.1002/acn3.51093

**Published:** 2020-06-12

**Authors:** Toshimitsu Suzuki, Toshifumi Suzuki, Matthieu Raveau, Noriko Miyake, Genki Sudo, Yoshinori Tsurusaki, Takaki Watanabe, Yuki Sugaya, Tetsuya Tatsukawa, Emi Mazaki, Atsushi Shimohata, Itaru Kushima, Branko Aleksic, Tomoko Shiino, Tomoko Toyota, Yoshimi Iwayama, Kentaro Nakaoka, Iori Ohmori, Aya Sasaki, Ken Watanabe, Shinichi Hirose, Sunao Kaneko, Yushi Inoue, Takeo Yoshikawa, Norio Ozaki, Masanobu Kano, Takeyoshi Shimoji, Naomichi Matsumoto, Kazuhiro Yamakawa

**Affiliations:** ^1^ Department of Neurodevelopmental Disorder Genetics Institute of Brain Science Nagoya City University Graduate School of Medical Science Nagoya Aichi 467‐8601 Japan; ^2^ Laboratory for Neurogenetics RIKEN Center for Brain Science Wako Saitama 351‐0198 Japan; ^3^ Department of Human Genetics Yokohama City University Graduate School of Medicine Yokohama Kanagawa 236‐0004 Japan; ^4^ Department of Obstetrics and Gynecology Juntendo University Faculty of Medicine Tokyo 113‐8421 Japan; ^5^ Faculty of Nutritional Science Sagami Women's University Sagamihara Kanagawa 252‐0383 Japan; ^6^ Department of Neurophysiology Graduate School of Medicine The University of Tokyo Tokyo 113‐0033 Japan; ^7^ Department of Psychiatry Nagoya University Graduate School of Medicine Nagoya Aichi 466‐8550 Japan; ^8^ Medical Genomics Center Nagoya University Hospital Nagoya Aichi 466‐8560 Japan; ^9^ Laboratory for Molecular Psychiatry RIKEN Center for Brain Science Wako Saitama 351‐0198 Japan; ^10^ National Epilepsy Center NHO Shizuoka Institute of Epilepsy and Neurological Disorders Aoi‐ku Shizuoka 420‐8688 Japan; ^11^ Department of Special Needs Education Okayama University Graduate School of Education Okayama 700‐8530 Japan; ^12^ Department of Pathology and Laboratory Medicine Tokyo Dental College Ichikawa General Hospital Ichikawa Chiba 272‐8513 Japan; ^13^ Section of Bone Function Department of Bone and Joint Diseases National Center for Geriatrics and Gerontology (NCGG) Obu Aichi 474‐8511 Japan; ^14^ Department of Pediatrics School of Medicine and Research Institute for the Molecular Pathomechanisms of Epilepsy Fukuoka University Fukuoka Fukuoka 814‐0180 Japan; ^15^ Department of Neuropsychiatry Hirosaki University Graduate School of Medicine Hirosaki Aomori 036‐8562 Japan; ^16^ North Tohoku Epilepsy Center Minato Hospital Hachinohe 031‐0813 Japan; ^17^ Department of Neurosurgery Okinawa Pref. Nanbu Medical Center and Children’s Medical Center Arakawa Haebaru Okinawa 901‐1193 Japan; ^18^ Present address: Department of Physiology Nippon Medical School Tokyo 113‐8602

## Abstract

**Objective:**

Neurodevelopmental disorders (NDDs) often associate with epilepsy or craniofacial malformations. Recent large‐scale DNA analyses identified hundreds of candidate genes for NDDs, but a large portion of the cases still remain unexplained. We aimed to identify novel candidate genes for NDDs.

**Methods:**

We performed exome sequencing of 95 patients with NDDs including 51 with trigonocephaly and subsequent targeted sequencing of additional 463 NDD patients, functional analyses of variant *in vitro,* and evaluations of autism spectrum disorder (ASD)‐like phenotypes and seizure‐related phenotypes *in vivo*.

**Results:**

We identified *de novo* truncation variants in nine novel genes; *CYP1A1*, *C14orf119*, *FLI1*, *CYB5R4*, *SEL1L2, RAB11FIP2, ZMYND8, ZNF143,* and *MSX2. MSX2* variants have been described in patients with cranial malformations, and our present patient with the *MSX2 de novo* truncation variant showed cranial meningocele and partial epilepsy. MSX2 protein is known to be ubiquitinated by an E3 ubiquitin ligase PJA1, and interestingly we found a *PJA1* hemizygous p.Arg376Cys variant recurrently in seven Japanese NDD patients; five with trigonocephaly and one with partial epilepsy, and the variant was absent in 886 Japanese control individuals. *Pja1* knock‐in mice carrying p.Arg365Cys, which is equivalent to p.Arg376Cys in human, showed a significant decrease in PJA1 protein amount, suggesting a loss‐of‐function effect of the variant. *Pja1* knockout mice displayed moderate deficits in isolation‐induced ultrasonic vocalizations and increased seizure susceptibility to pentylenetetrazole.

**Interpretation:**

These findings propose novel candidate genes including *PJA1* and *MSX2* for NDDs associated with craniofacial abnormalities and/or epilepsy.

## Introduction

Neurodevelopmental disorders (NDDs) are estimated to affect nearly 5% of children,^1^ and display a wide variety of phenotypes with various combinations of intellectual disability (ID), communication and social deficits, and delays in the acquisition of motor or language milestones. Even though recent large‐scale DNA sequencing studies allowed the identification of hundreds of candidate genes for NDDs,^2^, ^3^, ^4^ a large portion of the cases still remain unexplained. NDDs are often associated with comorbidities, among which epilepsy^5^ and craniofacial malformations^6^ are the most common. Across the various reports so far, patients showing craniofacial malformations have phenotypes ranging from microcephaly to macrocephaly, with a multitude of other forms affecting the shape and/or size of the skull. In previous works, we reported trigonocephaly, a form of craniosynostosis, in which the early closure of the metopic suture leads to a metopic ridge in patients affected with motor, learning and speech developmental delays.^7^, ^8^ In a collaborative work we have recently identified *de novo* truncating variants in *PHF21A* in three patients of NDD with macrocephaly and/or trigonocephaly.^9^

In this study, to identify novel candidate genes for NDDs, we performed exome or targeted sequencing on DNAs of 558 Japanese NDD patients with a rather predominant focus on those associated with trigonocephaly, and identified rare *de novo*, hemizygous, homozygous, and compound heterozygous variants in genes including functionally related *MSX2* and *PJA1*. Functional analyses of these variants *in vitro* and *in vivo* further supported that these are genes for NDDs.

## Materials and Methods

### Patients

All patients and in‐house control individuals analyzed were Japanese. For the exome sequencing, a total of 95 patients with neurodevelopmental disorders (NDD) associated with epilepsy and/or trigonocephaly from 85 families and 575 in‐house controls (male:281, female:294) were analyzed (Tables S1 and S2). Basically, the diagnostic criteria for autism symptom of patients with trigonocephaly were the score (9 or more points) of Pervasive Developmental Disorders – Autism Society Japan Rating Scale (PARS). For the targeted sequencing of *PJA1* and *MSX2,* an additional set of 463 patients with NDD associated with epilepsy and/or trigonocephaly and an additional independent set of 311 in‐house controls (male:181, female:130) were analyzed (Tables S2 and S3).

### Patient consent

The experimental protocols were approved by the Ethical Committee of RIKEN Institution and by the participating hospitals and universities. Written informed consents were obtained from all individuals and/or their families in compliance with the relevant Japanese regulations.

### Exome sequencing

Genomic DNAs were extracted from peripheral venous blood samples using QIAamp DNA Blood Midi Kit (Qiagen). Exome sequencing was performed as previously reported.^10^, ^11^ DNAs were captured using the SureSelect Human All Exon 50 Mb v5 kit (Agilent Technologies) or the SeqCap EZ Exome Library v2.0 (Roche NimbleGen) according to the manufacturer’s instructions and sequenced on a HiSeq2000 or HiSeq2500 system (Illumina). Reads were aligned to the human genomic reference hg19 (UCSC Genome Browser). After merging the BAM files of all members in each family using SAMtools, local realignments around insertion‐deletion variants (indels) and base quality score recalibration were performed with the Genome Analysis Toolkit.^12^ Variants registered in the dbSNP database (dbSNP135 or dbSNP137) which were not flagged as “clinically associated” as well as variants listed in the National Heart, Lung, and Blood Institute (NHLBI) exome variant server (EVS) database (ESP5400 or ESP6500 exomes) were excluded. We also excluded variants found in 575 in‐house control exomes previously sequenced in Yokohama City University Graduate School of Medicine. Variants that passed the filters were annotated using ANNOVAR.^13^ All variants were validated by Sanger sequencing.

### Targeted sequencing

We designed PCR primers to amplify candidate variants identified by exome sequencing as well as to screen all coding regions of *PJA1* (NM_145119) and *MSX2* (NM_002449). Genomic DNA from peripheral blood was amplified by PCR. Genomic DNA samples, OKI‐005‐5 and OKI‐005‐6, were extracted from saliva using Oragene DISCOVER (DNA Genotek Inc). Primer sequences and PCR conditions are available upon request. The PCR products were purified using ExoSAP‐IT PCR product Cleanup (Affymetrix) and analyzed by direct sequencing using an ABI PRISM 3730xl Genetic Analyzer.

### Model mice creation

*Pja1* and *Msx*2 recombinant lines were engineered using CRISPR/Cas9‐mediated mutagenesis as described by Wang and colleagues.^14^ Briefly, single‐guide RNA (sgRNA) were designed to target the *Pja1* (NM_001290555, Knockout: GGCTTCGGTACTTCCTGCGC AGG; knockin: GCTTCGGTACTTCCTGCGCA GGG) and the *Msx2* (NM_013601, CTATGGACAGGTACTGTTTCTGG). Forward and reverse oligonucleotides corresponding to these sgRNA were annealed and cloned into pX330‐U6‐Chimeric_BB‐CBh‐hSpCas9 plasmid (Plasmid #42230, Addgene) digested with BbsI.^15^

Cas9 mRNA (100 ng/μL) and sgRNAs (50 ng/μL) were microinjected into the cytoplasm of C57BL/6J fertilized eggs. For the *Pja1* knockin line, a single‐stranded oligodonor carrying the c.1093C>T point variant with c.1098G>A (silent variant) to destroy Protospacer Adjacent Motif (PAM) sequence was added to the injection mix (50 ng/μL). Resulting pups were screened and variants identified by DNA sequencing. These lines were backcrossed for two–three generations on C57BL/6J background before the start of the phenotype screening.

### Western blotting

To check PJA1 protein expression, brains were sampled from 9‐ to 10‐week‐old mice (*N* = 4 WT and 4 *Pja1* ^KI/Y^ and *N* = 3 WT and 3 *Pja1* ^KO/Y^). One hemisphere per sample was homogenized in ice‐cold 1X phosphate‐buffered saline (PBS) supplemented with protease inhibitors (Complete, Roche). Homogenates were centrifuged at 20,000*g* for 15 min. 20 μg of proteins was separated on 5–20% gradient SDS‐polyacrylamide gel (Super Sep Ace, Wako pure reagents) and performed Western blotting. The membrane was processed through sequential incubations with primary anti‐PJA1 rabbit polyclonal antibody (1:200 dilution, 17687‐AP, Proteintech) overnight at 4°C and then with secondary horseradish peroxidase (HRP) conjugated anti‐rabbit IgG antibody (1:10,000 Jackson Immuno Research). Labeled proteins were revealed using enhanced chemiluminescence (ECL) detection (Perkin‐Elmer). Membranes were re‐probed with anti‐GAPDH rabbit antibody (1:2,000; Santa Cruz Biotechnology) and revealed as described earlier. Band intensities were quantified using NIH ImageJ software (National Institute of Health).

### Gene expression analysis by RT‐qPCR

Brains were collected from 2‐to 3‐week‐old mice (*N* = 3 WT and 3 *Msx2* ^164fs/164fs^). Total RNA was prepared and cDNA was generated from 1μg of total RNA using Prime Script RT‐PCR kit with gDNA Eraser (TaKaRa). Real‐time quantitative PCR (RT‐qPCR) was performed in triplicates using SsoAdvanced Universal SYBR Green Supermix (Bio‐Rad) on an ABI PRISM 7900 thermocycler. Primers were designed using *Msx2* (NM_013601) as follows: Fwd‐ACCACATCCCAGCTTCTAGC and Rev‐CTTTTCGCCTTAGCCCTTCG. *Gusb* was used as housekeeper gene for gene expression level normalization using the following primers: Fwd‐ACTGACACCTCCATGTATCCCAAG and Rev‐CAGTAGGTCACCAGCCCGATG. Data were processed using manufacturer’s Sequence Detection System Ver2.4 software (Applied Biosystems).

### Animals and experimental conditions

All animal breeding and experimental procedures were performed in accordance with the ARRIVE guidelines and the guidelines of the Animal Experiments Committee of RIKEN Center for Brain Science. Independent groups of mice were generated for ultrasonic vocalization (*N* = 7 WT and 7 *Pja1* ^KO/Y^; *N* = 10 WT and 10 *Pja1* ^KI/Y^), postnatal milestones (*N* = 8 WT and 18 *Pja1* ^KO/Y^), behavior screening in adult mice (*N* = 12 WT and 12 *Pja1* ^KO/Y^; *N* = 12 WT and 12 *Pja1* ^KI/Y^), and seizure susceptibility to pentylenetetrazole (PTZ) (*N* = 18 WT and 26 *Pja1* ^KO/Y^). Independent groups were also generated for the *Msx2* knockout model for ultrasonic vocalization (*N* = 7 WT and 10 *Msx* ^164fs/+^) and behavior screening in adult mice (*N* = 11 WT and 13 *Msx2* ^164fs/+^).

### Ultrasonic vocalizations (USV)

Isolation‐induced USV were tested as previously described.^16^ Pups (postnatal day 6) were individually removed from the nest and placed in a plastic container on a layer of bedding chips. An experimenter blind to the genotype of the pups. Calls were classified into 10 categories commonly observed in C57BL/6 pups.^17^

### Open‐field

Open‐field test was performed as previously described.^16^ Mice (8 weeks old) were placed in a 60 × 60 cm square automated open‐field homogeneously. Data were acquired and analyzed using manufacturer’s tracking software (TimeOFCR4; O’Hara & Co).

### Social behavior: 3‐chambers task

The 3‐chambers test was performed as previously described.^16^ One wire quarter‐cylinder‐shaped cage was placed in a corner of the side chambers and used to enclose 8‐week‐old C57BL/6J stranger mice. *Pja1* ^KO/Y^ and *Msx2* ^164fs/+^ (12 weeks old) were tested. Data were acquired and analyzed using manufacturer’s tracking software (TimeCSI; O’Hara & Co).

The *Pja1* ^KI/Y^ (12 weeks old) was also tested. The trapping cages for stranger mice were wire cylinder‐shaped mesh cages (10 cm diameter, 15 cm high) placed at the center of the side chambers. The test was run as described previously^16^ and mice behavior was video‐recorded. Interactions (number and duration) as well as time spent in the different chambers were manually analyzed by an experimenter blind to the genotype.

### Tube test for social dominance

Tube test was performed as previously described.^16^ Mice (15 weeks old) were first allowed to run twice through the tube to habituate to the apparatus. Each mouse was submitted to four rounds with a different opponent every time. Mice combinations and starting side were randomized to prevent any bias.

### Barnes maze

The Barnes maze test was performed as previously described.^16^ Mice (17–19 weeks old) were first given a 5‐min habituation to the board and 1‐min habituation to a black Plexiglas escape box. The location of the box was randomized across the animals, but remained identical for a given mouse. A probe test (3‐min‐free exploration without escape box) was conducted 24 h after the last training session. Reverse training was conducted in similar way (four consecutive days, three trials per day), with the escape box placed 180° from its original location. A “reverse probe test” (3‐min‐free exploration without escape box) was conducted 24 h after the last reverse training trial. Data were acquired and analyzed using manufacturer’s tracking software (TimeBCM; O’Hara & Co).

### Seizure susceptibility

The protocol has been described previously.^18^ Briefly, pentylenetetrazole (PTZ; P6500, SIGMA Aldrich) dissolved in PBS was administered intraperitaneously (50 mg/kg). Animal behavior (9 weeks old) was then monitored and video‐recorded for a maximum of 10 min. Severity score were given on a scale from 1 to 5: (1) myoclonic, (2) tonic, (3) generalized seizure, (4) full body and limbs extension, and (5) death. Latency to generalized seizures and death were recorded and a maximum value of 600 sec was given to mice that did not reach these stages.

### Whole‐genome expression arrays

Hippocampus and prefrontal cortex were collected from *Pja1* ^KO/Y^ and *Msx2* ^164fs/164fs^ (4–6 weeks old) and their respective littermates (*N* = 3 per genotype) and flash frozen in liquid nitrogen. Total RNA was extracted and labeled using a Low Input Quick Amp Labeling kit (Agilent Technologies). Labeled cRNA were then hybridized onto Agilent SurePrint G3 Mouse Gene Expression 8x60K v2 arrays (Agilent Technologies). Arrays were scanned using a high‐resolution laser microarray scanner and data processed using the Agilent Feature Extraction software (Agilent Technologies). Further processing and statistical analyses were conducted using GeneSpring GX (Agilent Technologies) using filters for fold change > 2 and significance set at *P* < 0.05 for Student’s *t*‐test with Benjamini–Hochberg’s False Discovery Rate correction.

### Statistical analysis

Unless stated otherwise, statistical significance was assessed using one‐way ANOVA for parameters with a single value per individual and two‐way repeated measures ANOVA for parameters involving repeated measures. Statistical significance was calculated using KyPlot v2.0 software (Kyens Lab). Experiments were conducted in a blinded manner.

### Data availability

The data that support the findings of this study are available upon request.

## Results

### Identification of genes mutated in patients with NDD

First, we performed exome sequencing as previously described^19^ on a group of 95 NDD patients from 85 families including at least 51 cases associated with trigonocephaly and 40 with epilepsy (Tables S1 and S2). Parents were available for 69 families (79 patients), in which we identified 62 *de novo* variants in 38 patients affecting 57 genes and these variants were not found in our 575 in‐house control individuals (Table S4). Among these, truncation variants (frameshift, nonsense, and splice site) were found in 17 genes. Eight of those (*SCN1A, IQSEC2*, *STXBP1*, *CACNA1E, ARID1B, DDX3X*, *WHSC1*, *PHF21A*) have been known to be responsible for NDDs associated with IDs or epilepsies.^9^, ^20^, ^21^, ^22^, ^23^, ^24^, ^25^, ^26^, ^27^, ^28^, ^29^ The remaining nine (*MSX2*, *CYP1A1*, *C14orf119*, *FLI1*, *CYB5R4*, *SEL1L2, RAB11FIP2, ZMYND8,* and *ZNF143*) were novel for NDD so far to our knowledge.

**Figure 1:**
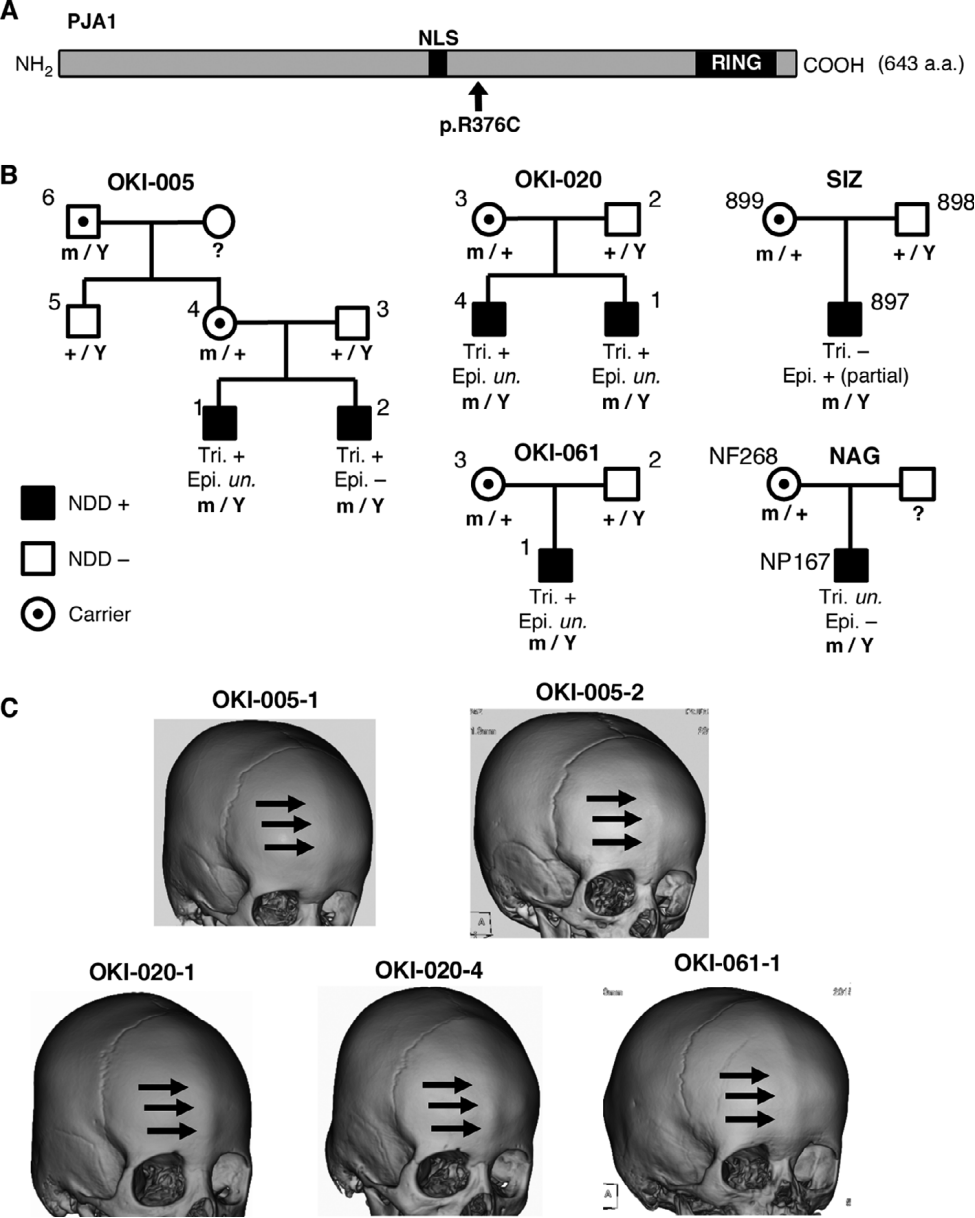
A recurrent missense variant in *PJA1* in patients with neurodevelopmental disorder and trigonocephaly. (A) The recurrent hemizygous missense variant c.1126C>T (p.Arg376Cys; p.R376C) is localized downstream from the nuclear localization signal (NLS) and upstream from the RING domain in PJA1 protein. (B) The p.R376C variant appeared in seven male patients from five unrelated families with neurodevelopmental disorder (NDD) associated with mild trigonocephaly (Tri.) or epilepsy (Epi.). The variant was identified by exome sequencing (families OKI‐005, OKI‐020, and SIZ) and targeted sequencing (families OKI‐061 and NAG). (C) Three‐dimensional computed tomography (3D‐CT) images acquired from the five individuals with mild trigonocephaly showed a characteristic metopic ridge (arrows).

The exome sequencing also identified 55 genes with hemizygous variants (Table S5), 16 genes with homozygous variants (Table S6), and 15 genes with compound heterozygous variants (Table S7) that were not found in our 575 in‐house control individuals. Remarkably, a hemizygous missense variant c.1126C>T (p.Arg376Cys; p.R376C) in the Praja ring finger ubiquitin ligase gene *PJA1* (Fig. 1A) was highly recurrent and together with a subsequent targeted sequencing of additional set of 463 independent cases of NDD (Tables S2 and S3), it appeared in seven male patients of NDD from five independent Japanese families (Fig. 1B, Tables S5 and S8, and Supplementary Data). All the *PJA1* p.R376C variants found in the seven NDD patients were inherited from their mothers who were not reported to have any symptoms. Among the seven patients, five (OKI‐005‐1, OKI‐005‐2, OKI‐020‐4, OKI‐020‐1, and OKI‐061‐1) were diagnosed with mild trigonocephaly, whereas one patient (SIZ‐897) without obvious craniofacial malformation displayed partial epileptic seizures (Fig. 1B and C, Table S8 and Supplementary Data). We succeeded to expand the pedigree tree only for the family OKI‐005 and found that *PJA1* p.R376C variant also appeared in maternal grand‐father without NDD (OKI‐005‐6) (Fig. 1B) which rather compromises the suspected pathological role of the variant itself (See Discussion). A haplotype analysis revealed that all the seven patients shared common haplotypes in a ~616 kb region spanning the *PJA1* p.R376C variant (Fig. S1), indicating a founder effect. In addition to *PJA1*, this 616 kb fragment contains two more genes: *LINC00269*, a predictive long intergenic nonprotein‐coding RNA, and *FAM155B*, a protein‐encoding gene of unknown function. Our exome sequencing did not identify any variants in *FAM155B* in the patients tested and did not cover *LINC00269*. Two additional heterozygous missense variants (c.623C>T and c.1457C>A; p.Ser208Phe and p.Pro486His, respectively) in *PJA1* were identified in independent female patients of NDD (SIZ‐978 and OKI‐011‐1), but both variants were inherited from hemizygous carrier fathers without NDD symptoms (Table S8 and Supplementary Data). These variants in *PJA1* were absent in our 886 in‐house Japanese control individuals (575 controls for exome sequencing and 311 additional independent controls for targeted sequencing) and absent or very rare in the genome aggregation database (gnomAD) (Table S8), in that the *PJA1* p.R376C variant was observed in non‐Asian populations at very low rate (Table S9).

PJA1 protein has been reported to ubiquitinate and degrade the transcription factor protein MSX2, a regulator of osteogenesis,^30^, ^31^ and variants in *MSX2* have been reported in cases of cranial malformations such as enlarged parietal foramina.^32^, ^33^ Interestingly, our analysis revealed a novel *de novo* frameshift variant c.516_517insG (p.Ala173fs: p.A173fs) in *MSX2* in a Japanese patient of autism spectrum disorder (ASD) associated with a cranial malformation (cranial meningocele) who also developed partial epileptic seizures (SIZ‐894, Tables S4 and S8), which is according to our knowledge the first report of a *de novo* truncation variant in *MSX2* in a patient with NDD. We further sequenced *MSX2* in the additional set of 463 independent cases of NDD (Tables S2 and S3), and found three missense variants c.74G>T, c.175C>T, and c.694G >A (p.Gly25Val, p.Pro59Ser, and p.Ala232Thr, respectively) in three patients (Table S8). These variants in *MSX2* were absent in our 886 in‐house Japanese control individuals and absent or very rare in the gnomAD (Table S8).

### *In vitro* and *in vivo* characterizations suggest a loss‐of‐function effect of the recurrent missense variant in *PJA1*

**Figure 2:**
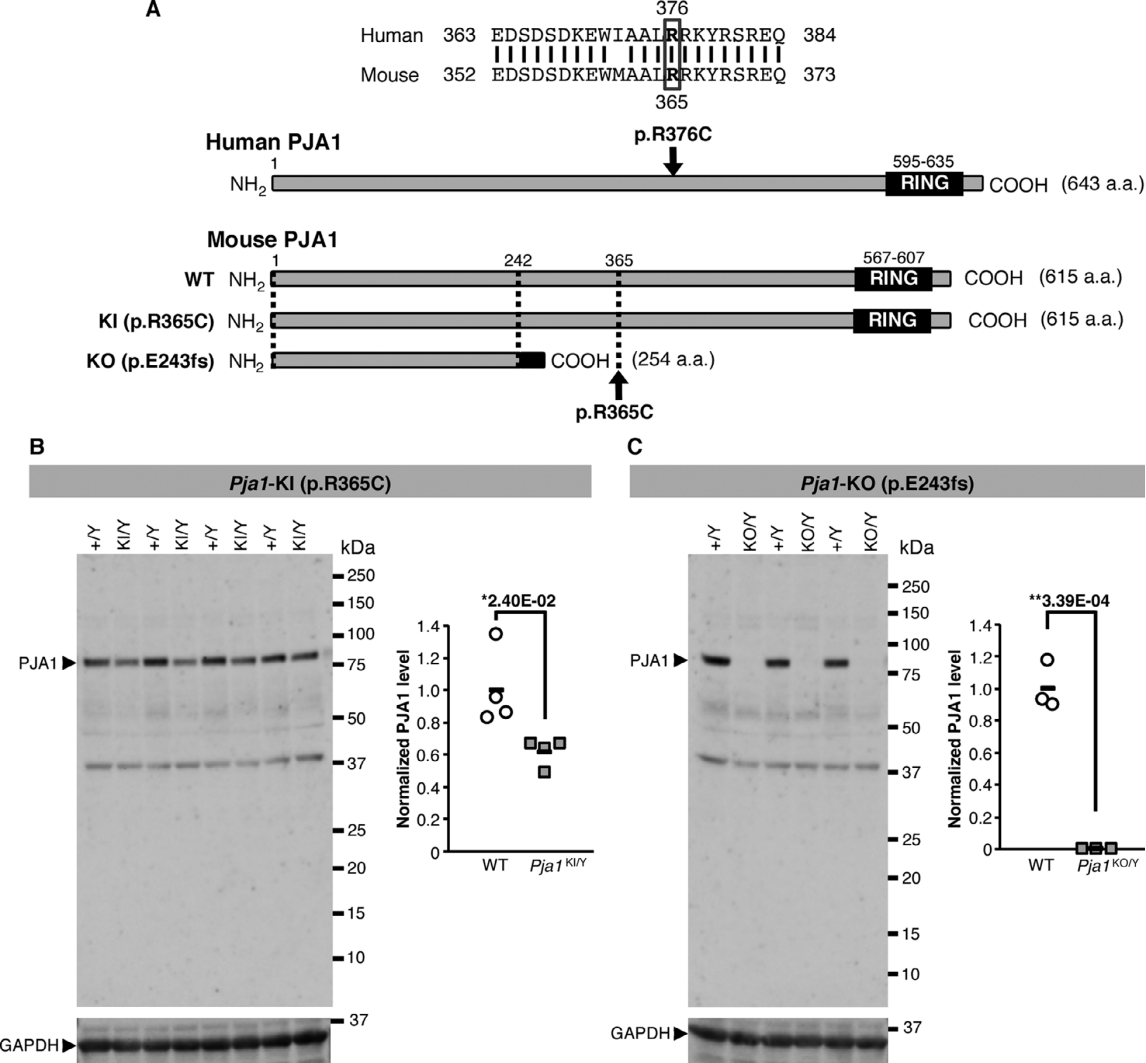
The recurrent *PJA1* variant in patients with NDD causes a drastic decrease in PJA1 protein amount in mice. (A) The amino acid sequence of PJA1 is highly conserved between human and mouse and the arginine residue #376 in human is found at #365 in mouse. A knock‐in mouse was generated by introducing the missense variant c.1093C>T (p.R365C) mimicking the c.1126C>T (p.R376C) variant identified in patients with NDD, and a knockout mouse was created with a frameshift c.729_744GGAACCGGTGGTGAGAdel (p.E243fs) at the amino acid position #243. (B, C) Western blots of proteins extracted from the mouse brains showed a significant decrease (38.1%) of PJA1 protein in the p.R365C hemizygous knock‐in mice (B) and a complete loss in the p.E243fs hemizygous knockout mice (C). Horizontal bars in B and C represent groups’ average values. One‐way ANOVA with significance set at (*) *P* < 0.05 and (**)*P* < 0.01.

We next assessed the functional consequence of the NDD/trigonocephaly associated *PJA1* p.R376C variant *in vivo* by generating a knock‐in mouse line (*Pja1* ^KI/Y^) with a c.1093C>T (p.R365C) variant, which is equivalent to the p.R376C variant in human, and a knockout mouse line (*Pja1* ^KO/Y^) with a *Pja1* frameshift truncation variant, c.729_744GGAACCGGTGGTGAGAdel (p.E243fs) (Fig. 2A). *Pja1* ^KI/Y^ and *Pja1* ^KO/Y^ mice did not show overt morphological abnormalities, and morphometric measurement of the skull did not reveal significant malformations in both lines (Fig. S2). We submitted *Pja1* ^KI/Y^ mice to a behavioral testing battery in order to investigate communication, spontaneous activity, learning, memory, and social skills. Although we observed a significant increase in winning rate in the social tube dominance test and a short yet significant delay in the reverse learning phase of the Barnes maze task in *Pja1* ^KI/Y^ animals, most of the parameters did not show significant changes between *Pja1* ^KI/Y^ mice and their wild‐type (WT) littermates (Figs. S3 and S4).

Western blot analyses revealed a significant decrease in PJA1 protein amount in the brain of *Pja1* ^KI/Y^ mice (Fig. 2B), and a complete loss in *Pja1* ^KO/Y^ mice as expected (Fig. 2C). PJA1 has been reported to indirectly bind to MSX2 via the DLXIN1 (MAGE‐D1) intermediary protein, ubiquitinate and degrade them.^30^ Because of the unavailability of reliable anti‐MSX2 antibodies (see Supplementary Data), instead we measured DLXIN1 protein amount in cortical extracts from the brains of *Pja1* ^KI/Y^ and *Pja1* ^KO/Y^ mice and found mild increases in DLXIN1 protein amounts in both models compared to their respective WT littermates, though the differences did not reach the statistical significance (Fig. S5).

We also investigated the functional impact of the mouse p.R365C variant *in vitro* (Fig. S6). The mouse p.R365C variant is found in the long isoform of PJA1, and corresponds to the p.R148C variant in the short isoform (Fig. S6A) which has previously been used to study the PJA1‐dependent ubiquitination and degradation of MSX2 and DLXIN1.^30^ We therefore generated expression constructs for the short isoform of PJA1 with p.R148C mutant or WT alleles, and transfected them into HEK293T cells together with a construct expressing mouse MSX2. Western blot analyses showed that MSX2 protein amount was significantly decreased in the presence of PJA1 and that this degradation was largely suppressed by the proteasome inhibitor MG132 (Fig. S6B) as previously reported.^30^ PJA1 also facilitated the ubiquitination of MSX2 (Fig. S6C) as previously shown.^31^ The p.R148C variant in PJA1 did not affect either of the PJA1‐dependent degradation or ubiquitination of MSX2 (Fig. S6B and C), suggesting that at equivalent amount of PJA1 protein the variant does not affect its function. However, because of the significant decrease in PJA1 protein amount in *Pja1* ^KI/Y^ mice (Fig. 2B), the ultimate effect of the p.R365C variant is presumably a loss‐of‐function.

### *Pja1* ^KO/Y^ mice show moderate deficits in isolation‐induced ultrasonic vocalizations and lack the preference for social novelty

Because of the assumption of the loss‐of‐function nature of the p.R376C variant, we performed behavioral analyses on *Pja1* ^KO/Y^ mice expecting to see clearer or stronger disease phenotypes than in *Pja1* ^KI/Y^ mice. We designed a postnatal behavioral screening (Fig. S7A) in order to assess the elementary developmental landmarks commonly seen in newborn and juvenile (from postnatal days 6 to 16, P6‐P16) mice^34^ and that can be altered in mouse models of neurodevelopmental disorders.^35^ We did not observe significant changes in *Pja1* ^KO/Y^ mice in the physical development (Fig. S7B) and the acquisition of developmental milestones for early (surface righting, negative geotaxis, forelimb grasp; Fig. S7C–E) and late stages (cliff aversion, open‐field, eye opening, auditory startle; Fig. S7F–I).

**Figure 3:**
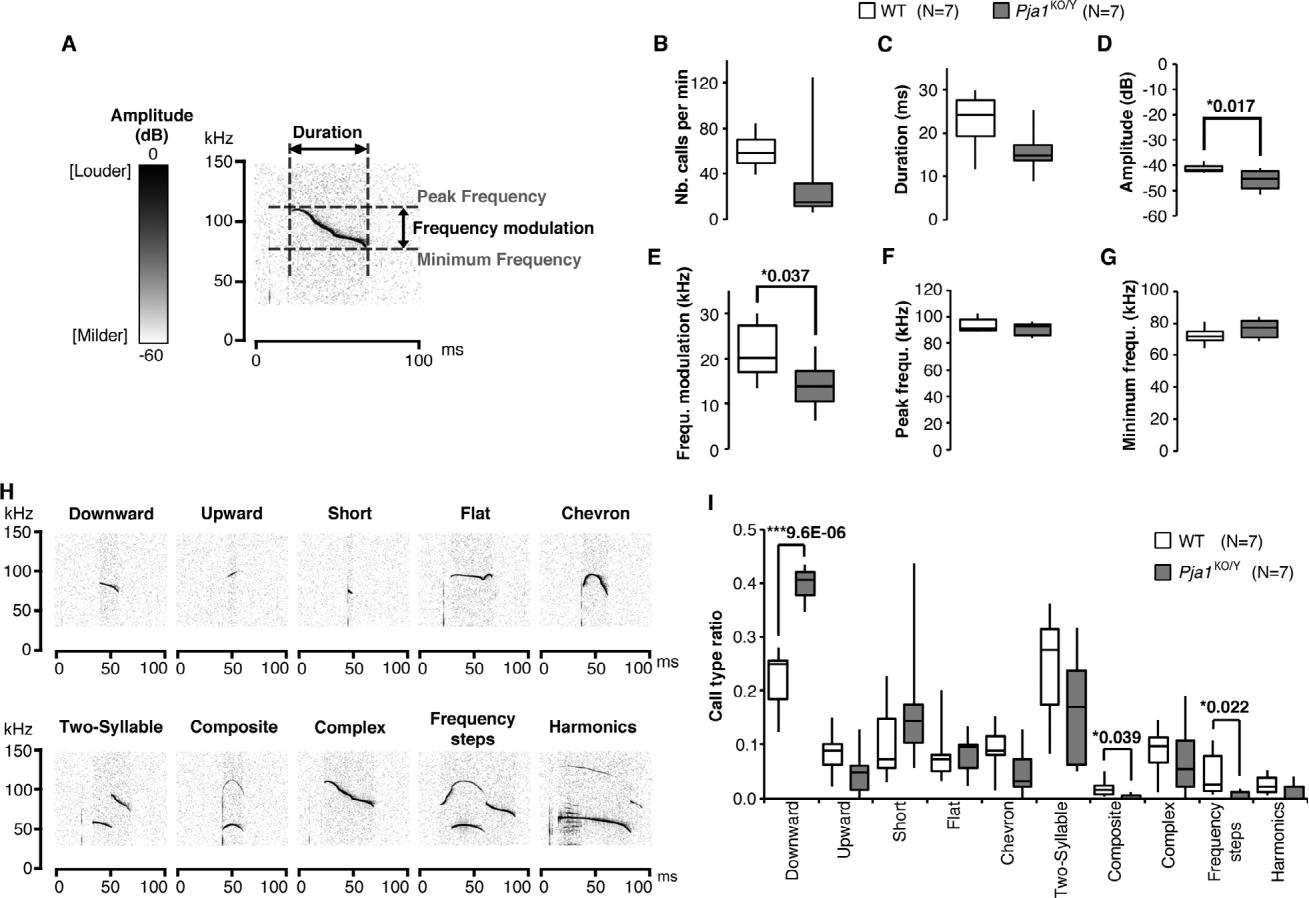
Moderate impairments in isolation‐induced ultrasonic vocalization in infantile *Pja1* ^KO/Y^ mice. (A) Isolation‐induced ultrasonic vocalizations (USV) produced by pups at postnatal day 6 were analyzed for intrinsic parameters relative to their amplitude, duration and frequency characteristics. (B–G) *Pja1* ^KO/Y^ pups showed tendencies for smaller number (B), shorter duration (C), significantly lower amplitude (D), and frequency modulation (E) of calls. Their peak frequency (F) and in minimum frequency (G) were mostly unchanged. (H, I) Calls were classified into 10 major categories commonly observed in C57BL/6 pups. Examples (H) were extracted from randomly chosen tracks from the WT group. The repertoire produced by *Pja1* ^KO/Y^ pups (I) showed a significant shift toward simple calls, with a significant increase in “downward” vocalizations at the expense of the more sophisticated “composite” and “frequency steps” types. One‐way ANOVA with significance set at (*) *P* < 0.05 and (***)*P* < 0.001.

ASD is characterized by a combination of social deficits, communication impairments and repetitive behavior or stereotypies. Behaviors mimicking these symptoms can be assessed in mouse and ASD‐like phenotypes have been reported in several renowned models such as BTBR or *Mef2c* and *Mecp2* knockout mice.^36^, ^37^, ^38^ Isolating pups from the mother mice triggers isolation‐induced ultrasonic vocalizations (USV) that tend to peak at P6.^39^ We performed quantitative and qualitative analyses of USV on *Pja1* ^KO/Y^ pups (Fig. 3). Significant decreases in amplitude, frequency modulation and a tendency for shorter vocalizations were observed in *Pja1* ^KO/Y^ pups compared to their WT littermates (Call duration: *P* = 0.052, Fig. 3B–G). USV calls can be classified into 10 main categories^17^ (Fig. 3H). The repertoire produced by *Pja1* ^KO/Y^ pups showed a significant shift toward “downward” calls at the expense of more complex calls as they produced fewer “composite” and “frequency steps” vocalizations (Fig. 3I).

**Figure 4:**
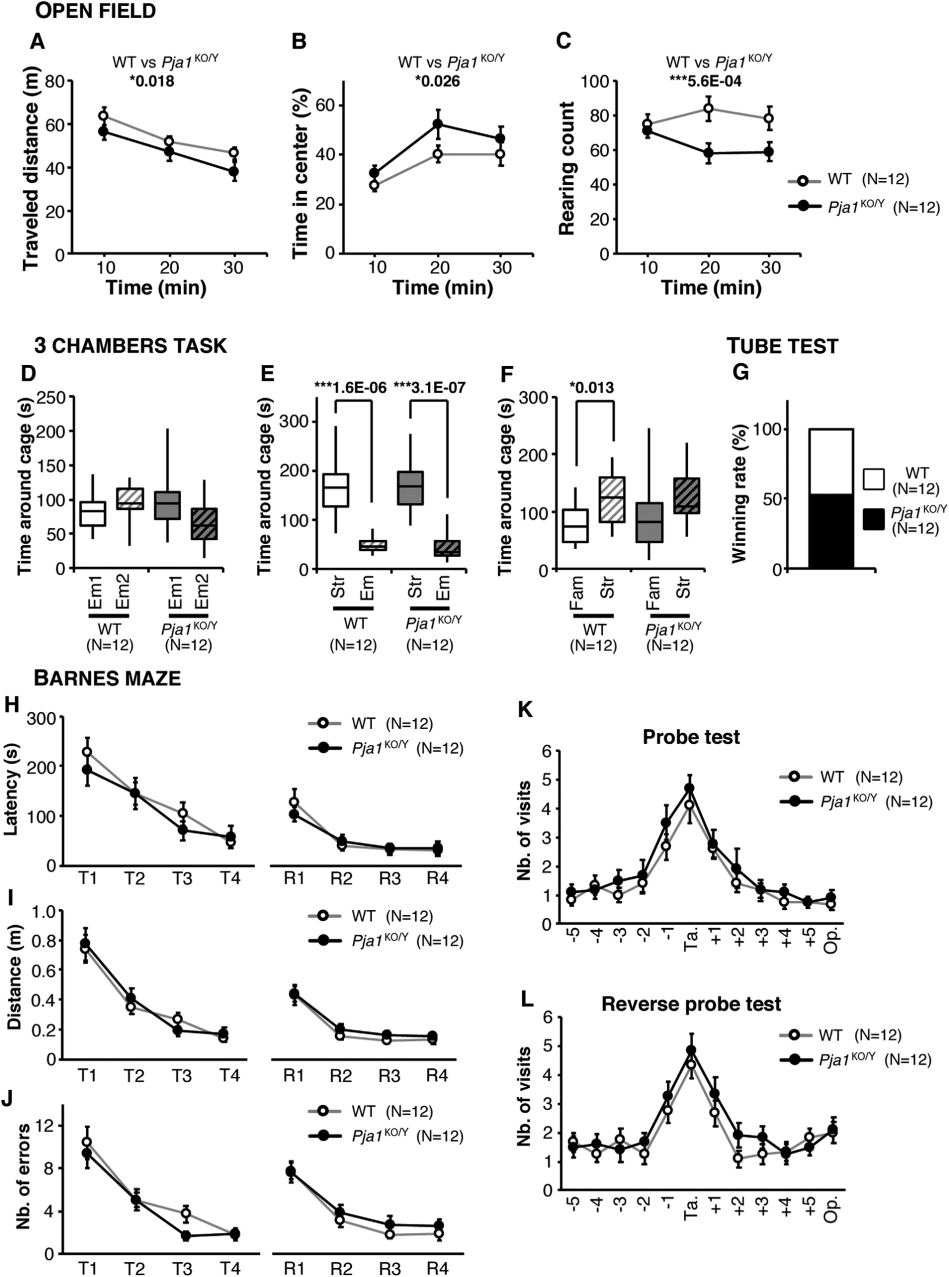
Impairments in exploratory behavior and social memory in *Pja1* ^KO/Y^ mice. (A–C) Open‐field task. *Pja1* ^KO/Y^ mice travelled slightly yet significantly shorter distances than their WT littermates (A) and spent significantly more time in the central area (B) suggesting a decrease in anxiety‐like behavior. The number of rearing was significantly decreased in the *Pja1* ^KO/Y^ group (C). (D–F) 3‐chambers task. While no preference for a specific side was seen in the habituation period (D), mice from both groups spent significantly more time investigating the stranger’s side (Str) than the empty side (Em) during the sociability phase of the task (E). In the preference for social novelty phase, whereas WT mice significantly spent more time on the stranger mouse (Str) than the familiar one (Fam), this discrimination did not reach the significance level in *Pja1* ^KO/Y^ mice (F). (G) In the tube test, the wining rate of *Pja1* ^KO/Y^ opposed to their WT littermates did not differ significantly from the 50% random outcome. (H–L) Barnes maze task. Spatial learning was conserved in *Pja1* ^KO/Y^ mice as they showed a similar latency (H), distance travelled (I), and number of errors (J) to reach the target hole during the primary learning (training T1 to T4) as well as the reverse learning periods (R1 to R4). In the probe test (K) as well as in the reverse probe test (L), the performance of *Pja1* ^KO/Y^ mice was not significantly different from that of their WT littermates. Ta.: target hole; Op.: Opposite side hole. Values in (A–C) and (H–L) are expressed as mean ± standard error of the mean. Two‐way ANOVA (A–C, H–L), Chi‐square test (G) or one‐way ANOVA (D–F) with significance set at (*) *P* < 0.05 and (***) *P* < 0.001.

We further performed a series of behavior screening on adult *Pja1* ^KO/Y^ mice (Fig. 4). *Pja1* ^KO/Y^ animals showed a mild, but significant decrease in exploratory activity (distance travelled and number of rearing) and anxiety (center stay) in the open‐field task (Fig. 4A–C). The 3‐chambers task, designed to investigate social behavior, did not show significant changes in sociability, but a mild deficit in the preference for social novelty, an index of social memory, in the *Pja1* ^KO/Y^ mice (Fig. 4D–F). The tube test, designed to assess social dominance, did not reveal any significant bias in wining rate between WT and *Pja1* ^KO/Y^ groups (Fig. 4G). Finally, we investigated hippocampus‐dependent memory using the Barnes maze task in which mice are required to learn the position of an escape box during a first learning phase and to learn the new position in a second “reverse” learning phase^40^ (Fig. 4H–L). In this task, *Pja1* ^KO/Y^ mice performance was not significantly different from their WT littermates indicating that spatial learning and memory were not altered in this model. In the course of this behavior screening we did not observe obvious signs of repetitive behavior (excessive grooming, circling, or insistence).

### *Pja1* ^KO/Y^ mice show increased seizure susceptibility to PTZ

**Figure 5:**
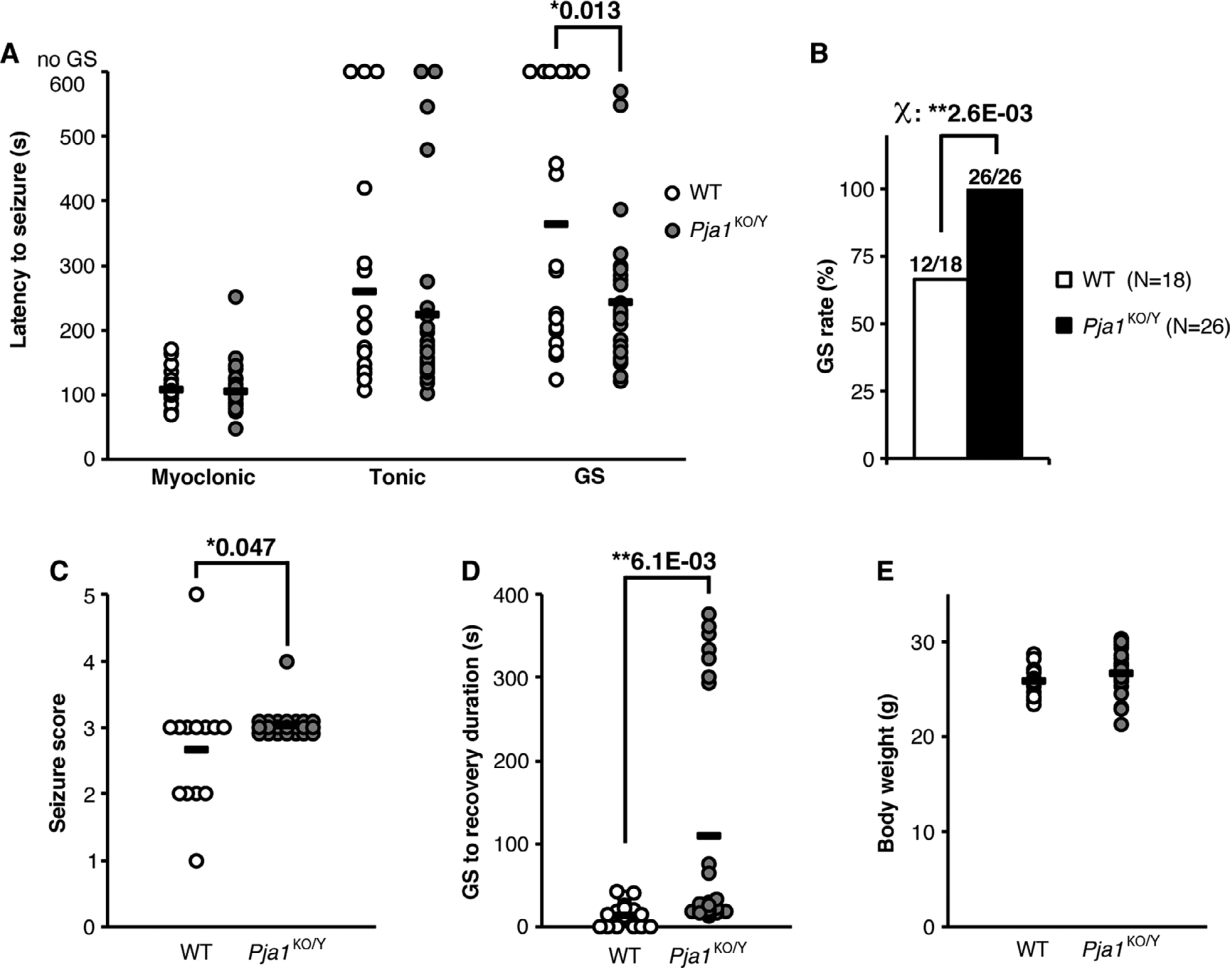
Increased seizure susceptibility in *Pja1* ^KO/Y^ mice. (A) Upon injection of pentylenetetrazole (PTZ, 50 mg/kg), whereas the latency to myoclonic and tonic seizures was not significantly different between the groups, the latency to generalized seizures (GS) was significantly decreased in *Pja1* ^KO/Y^ mice. (B) The percentage of mice that developed GS was significantly higher in the *Pja1* ^KO/Y^ group, all the mutant mice reaching that stage. (C) The seizure score was significantly higher in the *Pja1* ^KO/Y^ group. (D) Although WT mice recovered from GS in a short period, the time necessary for *Pja1* ^KO/Y^ mice to recover was significantly longer. (E) No difference in bodyweight between *Pja1* ^KO/Y^ mice and their WT littermates. Horizontal bars in A, C, D, and E represent groups average values. One‐way ANOVA (A, C, D, E) or Chi‐square test (B) with significance set at (*) *P* < 0.05 and (**) *P* < 0.01.

In rodents, pentylenetetrazole (PTZ), an inhibitor of GABA_A_ receptors, is most commonly used to model acute seizures and widely in the search for antiepileptic drugs.^41^, ^42^ Because an epileptic phenotype was reported in one of the patients with the p.R376C variant in *PJA1* and as we did not observe spontaneous epileptic seizures in *Pja1* ^KI/Y^ or *Pja1* ^KO/Y^ mice, we investigated seizure susceptibility to PTZ in *Pja1* ^KO/Y^ mice (Fig. 5). Although the latency to myoclonic and tonic seizures did not differ significantly, the latency to generalized convulsive seizures (GS) was significantly shorter and seizure severity significantly stronger in *Pja1* ^KO/Y^ mice at 9 weeks old (Fig. 5A–C). Moreover, the time necessary to recover from GS was significantly longer in the *Pja1* ^KO/Y^ group (Fig. 5D). *Pja1* ^KO/Y^ mice are the first to our knowledge to show such a prolonged recovery‐time.

### *Msx2* ^165fs/+^ pups display a mild deficit in isolation‐induced ultrasonic vocalizations, whereas overall behavioral characteristics are conserved in adult mice

In the present work, we also identified variants in *MSX2* in four patients with NDD including one with a truncation variant (p.A173fs) showing partial epilepsy as well (Tables S4 and S8). We therefore generated *Msx2* knockout lines (*Msx*2^164fs/+^ and *Msx2* ^165fs/+^) carrying c.491_492insC and c.492_493insA truncations by frameshift (p.K164fs and p.Q165fs, respectively), mimicking the p.A173fs variant in the patient with NDD and partial epilepsy (Fig. S8A and B) and investigated whether these mice show NDD‐like phenotypes. Homozygous knockout mice (*Msx*2^164fs/164fs^ and *Msx2* ^165fs/165fs^) showed the characteristic absence of fur and significant growth delay (Fig. S8C and D), as previously reported in *Msx2* ^−/−^ mice.^43^ However, *Msx*2^164fs/164fs^ and *Msx2* ^165fs/165fs^ did not show cranial foramen and generalized seizure‐like episodes that were reported in *Msx2* ^−/−^ mice,^43^ the reason for this discrepancy is currently unknown. At P6, *Msx*2^164fs/+^ pups produced significantly fewer USV calls than their WT littermates, but other parameters including the repertoire, were not significantly altered (Fig. S9). In adult *Msx*2^164fs/+^ mice, we did not observe significant changes in exploratory behavior, social behavior (including social memory and social dominance), and spatial learning and memory (Fig. S10).

### Expression profiling in the brain of *Pja1* ^KO/Y^ and *Msx*2^164fs/164fs^ mice

We further performed DNA microarray analyses and measured whole‐genome level mRNA expression changes in the prefrontal cortex and hippocampus of *Pja1* ^KO/Y^ and *Msx*2^164fs/164fs^ mice. *Pja1* ^KO/Y^ mice showed 2.5‐fold decrease in *Pja1* mRNA expression in both cortex and hippocampus, suggesting that the p.E243fs variant in the mice decreased the stability of *Pja1* mRNA. Significant decreases in mRNAs were also observed for *Klhl31*, AU023639, *Acsl1,* and *Klfl7* (by 2.8‐, 2.3‐, 2.2‐, and 2.1‐fold, respectively), potentially contributing to the disease phenotypes in *Pja1* ^KO/Y^ mice. In *Msx*2^164fs/164fs^ samples, we did not identify significantly deregulated genes (data not shown) suggesting that changes in genome‐wide genes' expressions were rather limited at the mRNA level.

## Discussion

In this study, we have identified nine novel *de novo* truncation variants in patients of NDD: *CYP1A1*, *C14orf119*, *FLI1*, *CYB5R4*, *SEL1L2*, *RAB11FIP2*, *ZMYND8*, *ZNF143,* and *MSX2*. More surprisingly, we have found a recurrent hemizygous missense variant in *PJA1* in a total of seven male patients with NDDs, five of which also had a mild trigonocephaly. Although several contiguous gene deletions including *PJA1* locus have been reported in cases of craniofacial malformations,^44^ according to our knowledge the present work is the first report of human diseases specifically associated with *PJA1* variants. PJA1, a member of the E3 ubiquitin ligase family, was originally identified as a component of embryonic development,^45^ but its potential role in craniofacial and brain development was unknown to date. In one family, the hemizygous *PJA1* p.R376C variant was inherited from the maternal grandfather without known NDD symptoms suggesting that the variant may have an incomplete penetrance or present a somatic mosaicism in healthy carriers. Although *PJA1* p.R376C was otherwise associated with NDD phenotypes, and despite our exome sequencing not identifying other candidate variants in a ~616 kb region with common haplotypes in all patients tested, we do not exclude the possibility of an oligogenic effect with additional genetic modifiers acting in combination to *PJA1* in cases of NDD. Further studies using large set of patients with NDD are required to elucidate the causal relationship between *PJA1* variants and NDD in detail.

In order to investigate the functional consequences of the variant or a loss of *Pja1* we have generated models of the missense *in vitro* and models of the missense and a knockout *in vivo*. Although the *in vitro* study did not identify a clear impact of the missense variant on the ability of PJA1 to ubiquitinate and degrade its targets DLXIN1 and MSX2, it resulted in a significant decrease in PJA1 protein level in the brain of the mice with the equivalent missense variant through an as‐yet unknown mechanism. Furthermore, the knockout mice *Pja1* ^KO/Y^ showed moderate deficits in isolation‐induced USVs, a widely used behavioral measure to assess developmental delays and communication deficits in rodent models for ASD, and increased seizure susceptibility to PTZ. Although it is still unknown for the exact effect of missense variant, together with the observation of mild increases in DLXIN protein amount in *Pja1* ^KI/Y^ and *Pja1* ^KO/Y^ mice, these results suggest that the ultimate effect of the mouse p.R365C and human p.R376C variants in *PJA1* is a loss‐of‐function that may lead to an increase in MSX2 protein amount in *Pja1* ^KI/Y^ and *Pja1* ^KO/Y^ mice as well as in patients with the p.R376C variant. Such loss‐of‐function effect of *PJA1* p.R376C variant in patients with NDD/trigonocephaly would be consistent to the previous observations of craniosynostosis in patients with 5q‐partial trisomy spanning *MSX2* ^46^, ^47^ and in transgenic mice overexpressing MSX2^48^ in that increases of MSX2 protein could contribute to their pathological phenotypes.

Assuming a loss‐of‐function effect for the missense variant *PJA1* p.R376C, we have designed a behavioral screening using *Pja1* ^KO/Y^ model mice completely lacking PJA1 protein. Juvenile *Pja1* ^KO/Y^ mice showed moderate deficits in isolation‐induced USVs resembling the deficits in mice with haploinsufficiency of *Dyrk1a*, a major candidate gene for a syndromic form of ASD.^16^ These vocalization deficits may mimic the speech learning delays observed in all of the patients with the *PJA1* p.R376C hemizygous variant. In adults, aside from a decrease in spontaneous exploratory activity and signs of decreased anxiety, *Pja1* ^KO/Y^ mice had a mild deficit in the 3‐chambers social task, lacking the preference for social novelty commonly observed in rodents. Overall, *Pja1* ^KO/Y^ mice displayed only a few behavioral differences as compared to WT, with minimal relevance to the symptoms of ASD. Additionally, we investigated *Msx*2^164fs/+^ mice lacking a copy of *Msx2*, one of the targets for PJA1‐mediated ubiquitination. Though milder than in *Pja1* ^KO/Y^ pups, juvenile *Msx*2^164fs/+^ mice also showed a mild deficit in isolation‐induced USVs. Comparatively moderate NDD‐like phenotypes of *Msx2*‐deficient mice may suggest that additional genetic or environmental factors are required to cause the full disease phenotype.

In conclusion, our results may indicate that variants of functionally related *PJA1* and *MSX2* genes contribute to NDDs associating with epilepsy and/or craniofacial abnormalities and also indicate that it would be worthwhile to precisely investigate the phenotypes of *Pja1* and *Msx2* mutant mice in the future.

## Conflict of Interest

Nothing to report.

## Supporting information


**Figure S1.** Common haplotypes in a ~616 kb region surrounding the p.Arg376Cys variant in *PJA1* in all 7 NDD patients.
**Figure S2.** Conserved skull morphology in *Pja1* ^KI/Y^ and *Pja1* ^KO/Y^ mice.
**Figure S3.** Conserved exploratory behavior, social behavior, and spatial learning/memory in *Pja1* ^KI/Y^ mice.
**Figure S4.** Isolation‐induced ultrasonic vocalizations are conserved in *Pja1* ^KI/Y^ pups.
**Figure S5.** DLXIN1 protein amount is not significantly changed in brains of *Pja1* ^KI/Y^ and *Pja1* ^KO/Y^ mice.
**Figure S6.** The R148C variant in PJA1 does not affect proteasome‐mediated degradation of MSX2.
**Figure S7.** Unaffected developmental milestones in *Pja1* ^KO/Y^ mice
**Figure S8.** Modeling of the *MSX2 de novo* p.A173fs frameshift variant found in a patient of NDD recapitulates knockout phenotype in mice.
**Figure S9.** Decreased isolation‐induced vocalizations in *Msx2* ^164fs/+^ pups without changes in call properties or vocalization repertoire.
**Figure S10.** Conserved exploratory behavior, social behavior, and spatial learning/memory in *Msx2* ^164fs/+^ mice.

**Table S1.** 95 NDD patients, their parents and associated phenotypes for whole‐exome sequencing.
**Table S2.** Number of NDD patients per associated phenotypes and analysis.
**Table S3.** 463 NDD patients, their parents and associated phenotypes for targeted sequencing
**Table S4.** *De novo* mutations identified by whole‐exome sequencing.
**Table S5.** Hemizygous mutations identified by whole‐exome sequencing.
**Table S6.** Homozygous mutations identified by whole‐exome sequencing.
**Table S7.** Compound heterozygous mutations identified by whole‐exome sequencing.
**Table S8.** *PJA1* and *MSX2* nonsynonymous mutations and phenotypic features in individuals with the mutations.
**Table S9.** Allele frequency of PJA1 p.Arg376Cys mutation in various populations.
