# Supplementary material for: A recurrent PJA1 variant in trigonocephaly and neurodevelopmental disorders
Source: Ann Clin Transl Neurol. 2020 Jun 12;7(7):1117–31. doi: 10.1002/acn3.51093 (PMC7359110; doi:10.1002/acn3.51093)
Supplement: Supplementary file 1 — Figure S1. Common haplotypes in a ~616 kb region surrounding the p.Arg376Cys variant in PJA1 in all 7 NDD patients. Figure S2. Conserved skull morphology in Pja1 KI/Y and Pja1 KO/Y mice. Figure S3. Conserved exploratory behavior, social behavior, and spatial learning/memory in Pja1 KI/Y mice. Figure S4. Isolation‐induced ultrasonic vocalizations are conserved in Pja1 KI/Y pups. Figure S5. DLXIN1 protein amount is not significantly changed in brains of Pja1 KI/Y and Pja1 KO/Y mice. Figure S6. The R148C variant in PJA1 does not affect proteasome‐mediated degradation of MSX2. Figure S7. Unaffected developmental milestones in Pja1 KO/Y mice Figure S8. Modeling of the MSX2 de novo p.A173fs frameshift variant found in a patient of NDD recapitulates knockout phenotype in mice. Figure S9. Decreased isolation‐induced vocalizations in Msx2 164fs/+ pups without changes in call properties or vocalization repertoire. Figure S10. Conserved exploratory behavior, social behavior, and spatial learning/memory in Msx2 164fs/+ mice. [file ACN3-7-1117-s001.pdf]

Genotype	OKI-005-1	A	C	G	C	T	A	C	T	G	A	G	**T**	T	A	G	G	A	C	G	G	A	G
	OKI-005-2	A	C	G	C	T	A	C	T	G	A	G	**T**	T	A	G	G	A	C	G	G	A	G
	OKI-020-4	A	C	G	C	T	A	C	T	G	A	G	**T**	T	A	G	G	A	C	G	G	A	G
	OKI-020-1	A	C	G	C	T	A	C	T	G	A	G	**T**	T	A	G	G	A	C	G	G	A	G
	SIZ-897	A	C	G	A	C	A	C	T	G	A	G	**T**	T	A	G	G	A	C	G	A	C	A
	OKI-061-1	A	C	G	C	T	A	C	T	G	A	G	**T**	T	A	G	G	A	C	G	G	A	G
	NAG-NP167	A	T	G	C	T	A	C	T	G	A	G	**T**	T	A	G	G	A	C	G	G	A	G
Nucleotide change		A>G	T>C	C>G	C>A	T>C	A>G	T>C	C>T	A>G	C>A	C>G	**C>T**	C>T	A>G	G>A	G>T	A>G	G>C	G>T	A>G	C>A	A>G
MAF in 1kG		0.422	0.447	0.384	0.387	0.421	0.495	0.417	0.325	0.337	0.485	0.191	**0.000**	0.437	0.465	0.402	0.425	0.490	0.418	0.421	0.357	0.473	0.444
Position (build GRCh37/hg19)		67,970,237	68,058,027	68,083,221	68,108,910	68,112,868	68,123,244	68,181,227	68,187,998	68,188,255	68,207,989	68,353,870	**68,381,956**	68,384,125	68,388,145	68,389,129	68,389,459	68,394,966	68,398,239	68,400,821	68,729,506	68,732,665	68,734,955
Distance from rs745369655 (kb)		412	324	299	273	269	259	201	194	194	174	28	**0**	2	6	7	8	13	16	19	348	351	353
SNP ID		rs5936898	rs554433	rs421343	rs241745	rs241742	rs241739	rs4844159	rs5936651	rs5937128	rs992688	rs6625400	**rs745369655 (p.Arg376Cys)**	rs6624385	rs5936661	rs4596804	rs5937164	rs5981179	rs1926355	rs5981182	rs501342	rs1171943	rs2025825

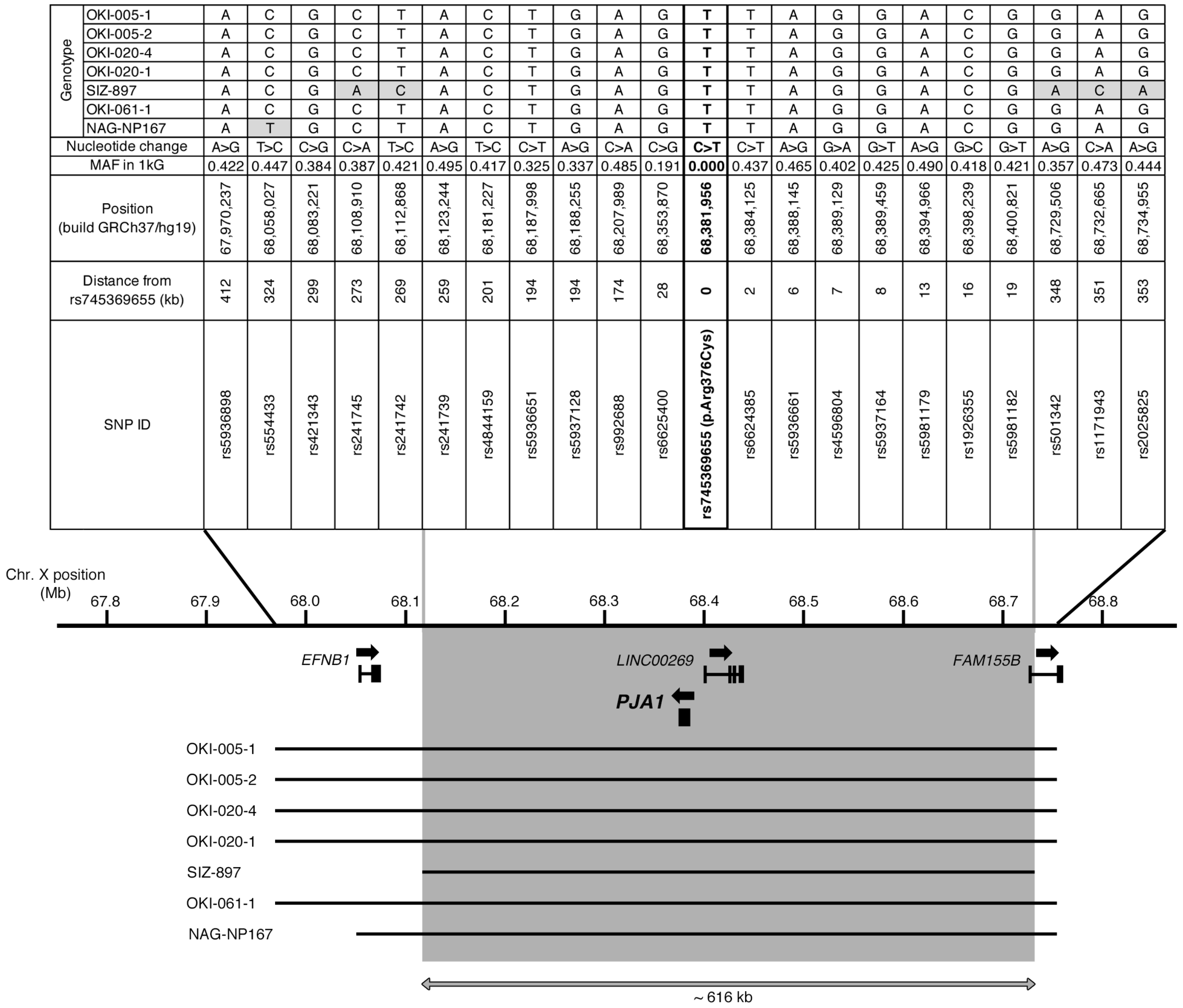

Supplementary Figure 1 | Common haplotypes in a ~616 kb region surrounding the p.Arg376Cys variant in *PJA1* in all 7 NDD patients. Haplotypes of NDD patients surrounding the *PJA1* locus for a selection of single nucleotide polymorphisms (SNP) with their respective nucleotide change, minor allele frequency in 1000 Genomes Browser database (MAF in 1kG) and genetic position. Common haplotypes suggest a founder effect for the p.R376C variant in these 7 patients. Two patients (SIZ-897 and NAG-NP-167) show different genotypes in 6 distant markers (light grey highlight). Common SNP analysis shows a ~616 kb minimal common region (dark grey highlight) containing two additional genes, *LINC00269* and *FAM155B*. No candidate variants were identified for these genes in our exome sequencing analysis (Supplementary Tables 4-7).

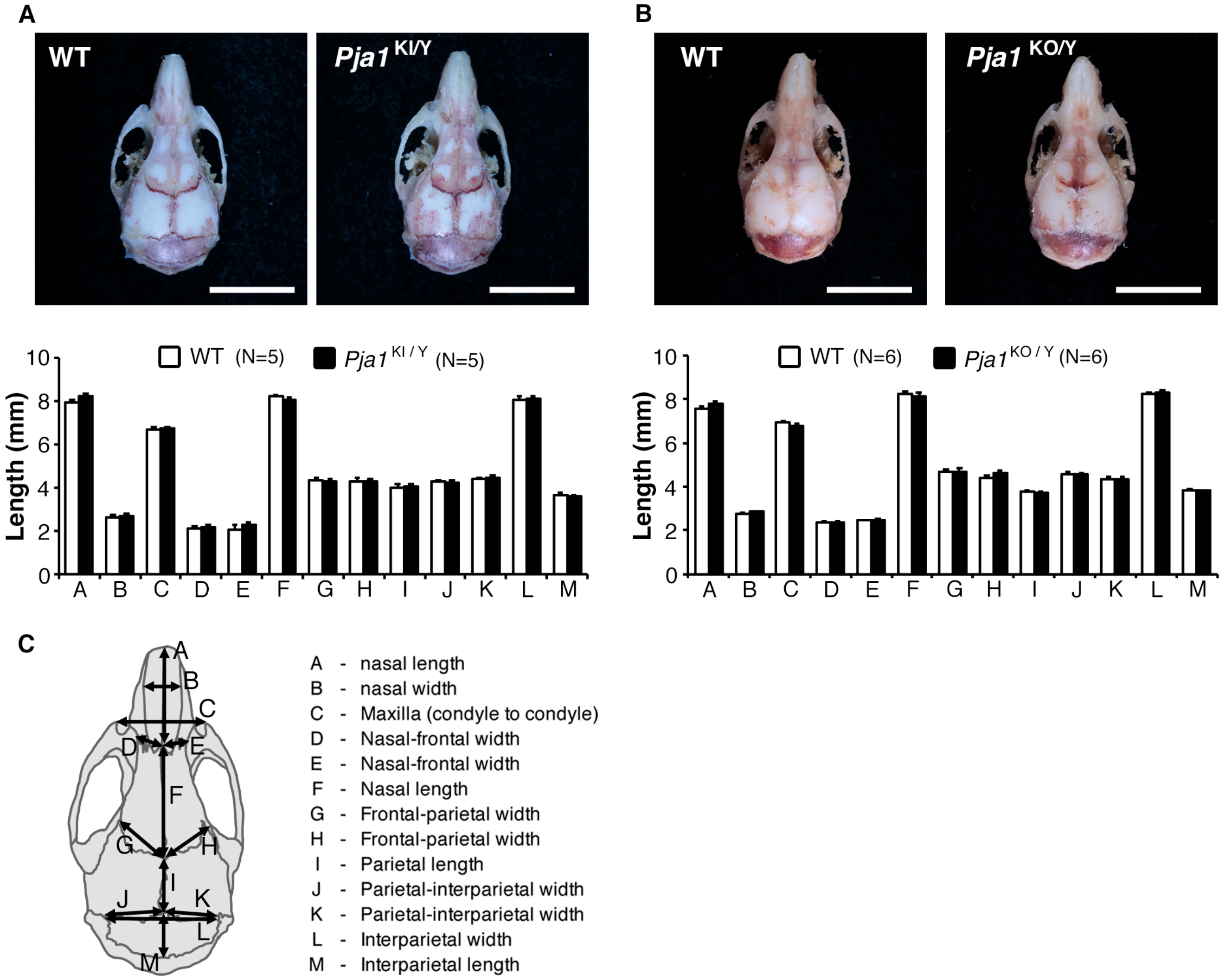

Supplementary Figure 2 | Conserved skull morphology in *Pja1*$^{KI/Y}$ and *Pja1*$^{KO/Y}$ mice. Skulls from 5 months old mice fixed in 70% ethanol were prepared for a simple morphometric comparative study. Skulls from *Pja1*$^{KI/Y}$ (**A**) and *Pja1*$^{KO/Y}$ (**B**) mice did not show overt differences compared to their respective WT littermates. Precise measurement did not reveal significant differences for a selection of thirteen characteristic distances using suture junction points as landmarks (**C**). Scale bars correspond to 1 cm. Values are expressed as mean ± standard error of the mean. One-way ANOVA with significance set at $p<0.05$.

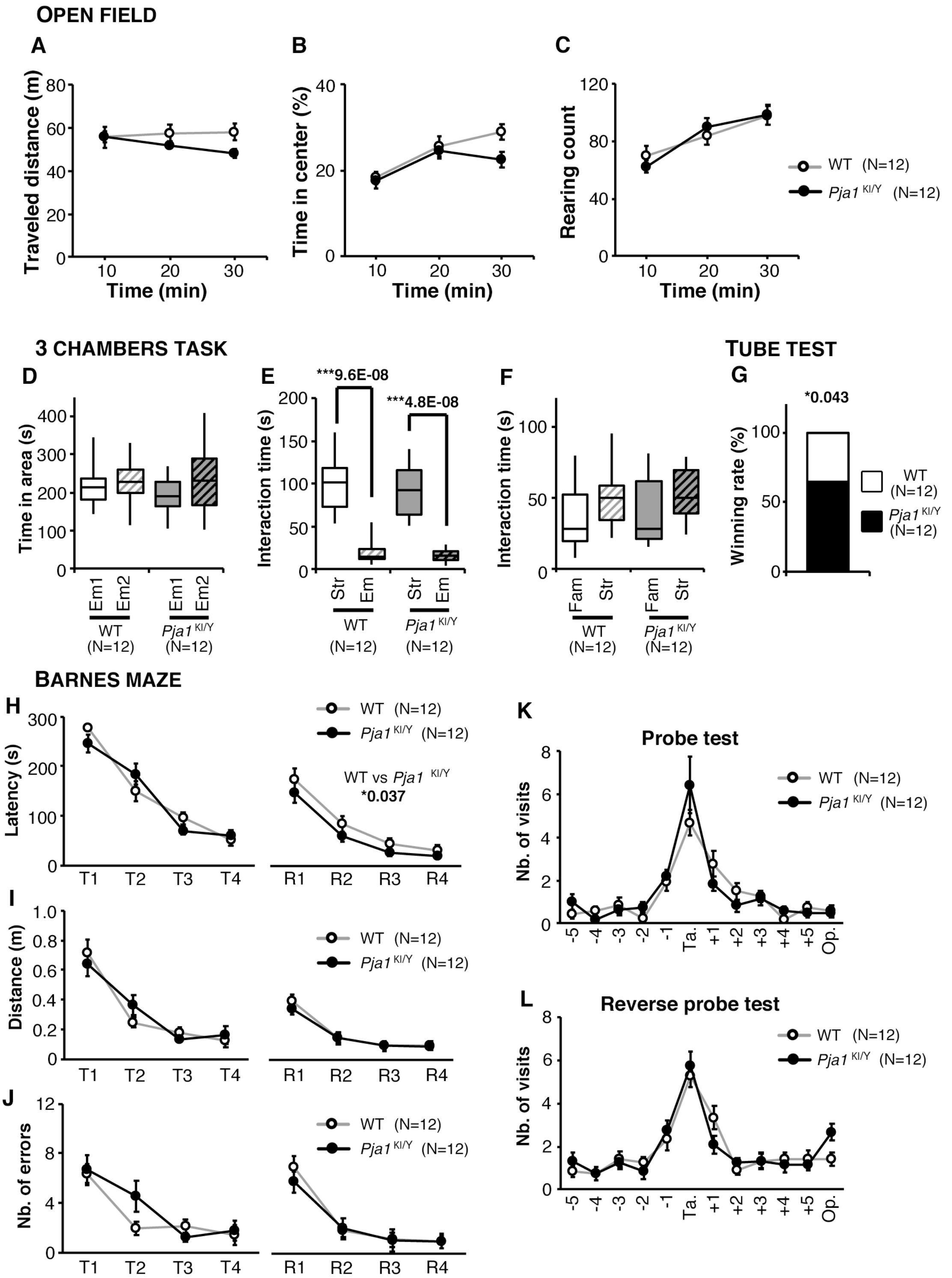

Supplementary Figure 3 I Conserved exploratory behavior, social behavior and spatial learning/memory in *Pja1*$^{KI/Y}$ mice. In the open field task, *Pja1*$^{KI/Y}$ mice did not show significant differences compared to WT littermates in terms of travelled distance (**A**), time spent in the central area (**B**) or number of rearing (**C**) during the 30 minutes of the task. In the 3 chambers task, while no preference for a specific side was seen in the habituation period (**D**), mice from both groups spent significantly more time investigating the stranger's side (Str) than the empty side (Em) during the sociability phase of the task (**E**). In the preference for social novelty phase, the performance of WT and *Pja1*$^{KI/Y}$ mice did not differ significantly, though both groups did not show a significant preference for the stranger mouse over the familiar one (**F**). (**G**) In the tube test, *Pja1*$^{KI/Y}$ had a higher winning rate when opposed to their WT littermates, differing significantly from a 50% random outcome. In the Barnes maze task, spatial learning was conserved in *Pja1*$^{KI/Y}$ mice as they showed a similar latency (**H**), distance travelled (**I**) and number of errors (**J**) to reach the target hole during the primary learning phase, whereas a short yet significant increase in the latency to reach the target was seen in the reverse learning phase of the task. In the probe test (**K**) as well as in the reverse probe test (**L**) the performance of *Pja1*$^{KI/Y}$ mice was not significantly different from that of their WT littermates. Values in (A-C) and (H-L) are expressed as mean ± standard error of the mean. Two-way ANOVA (A-C, H-L), Chi-square test (G) or one-way ANOVA (D-F) with significance set at (*) $p<0.05$ and (***) $p<0.001$.

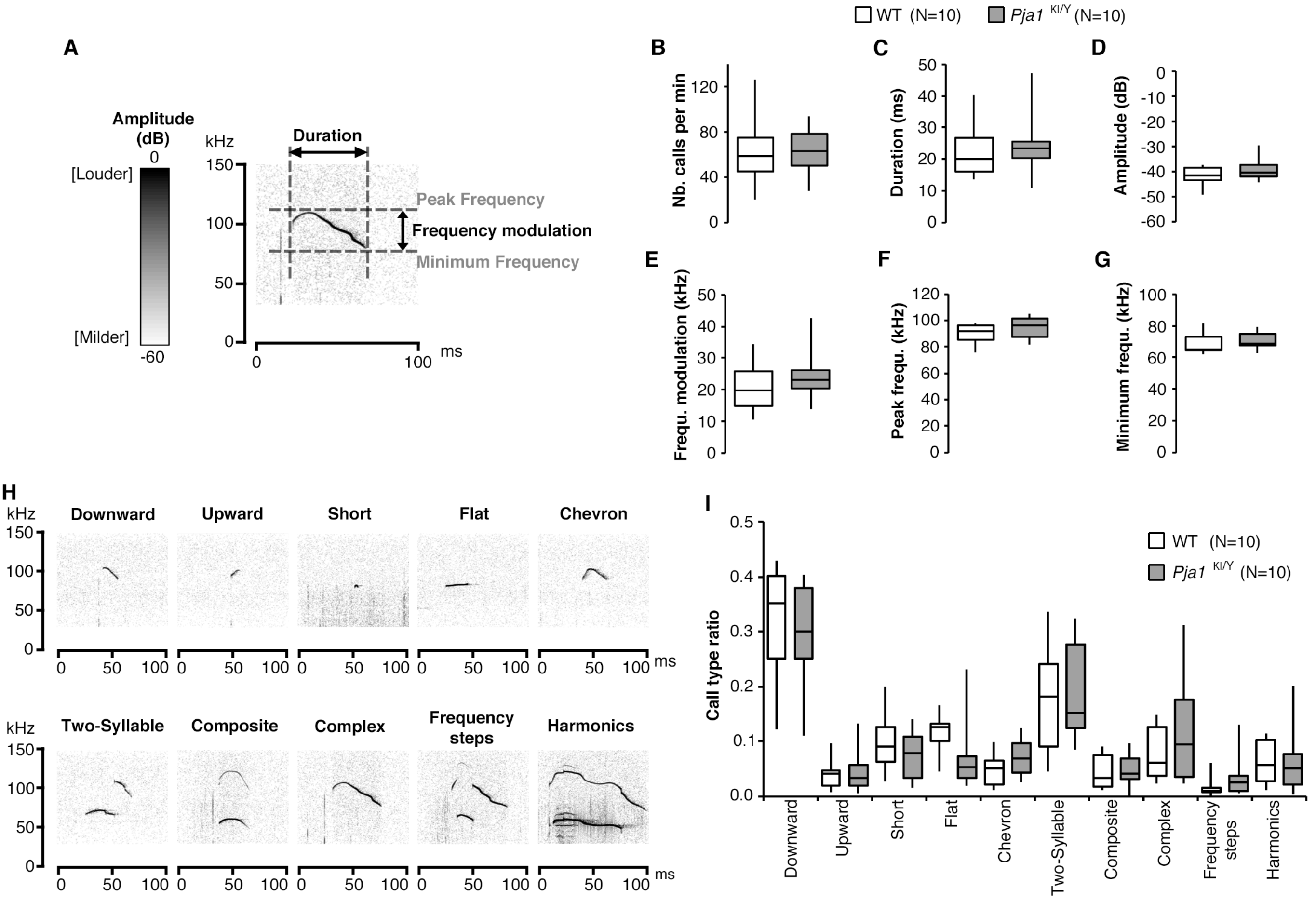

Supplementary Figure 4 | Isolation-induced ultrasonic vocalizations are conserved in *Pja1*$^{KI/Y}$ pups. (**A**) Isolation induced ultrasonic vocalizations (USV) produced by pups at postnatal day 6 were analyzed for intrinsic parameters relative to their amplitude, duration and frequency. (**B**) The number of calls produced by *Pja1*$^{KI/Y}$ pups was not significantly different from their WT littermates. The average call duration (**C**), amplitude (**D**), frequency modulation (**E**), peak frequency (**F**) and minimum frequency (**G**) of USV calls were not significantly affected in *Pja1*$^{KI/Y}$ pups. (**H**) All calls were classified into ten major categories commonly observed in C57BL/6 pups. Examples displayed were extracted from randomly chosen tracks from the WT group. (**I**) The repertoire produced by *Pja1*$^{KI/Y}$ pups did not differ significantly from that of their WT littermates. One-way ANOVA with significance set at $p<0.05$.

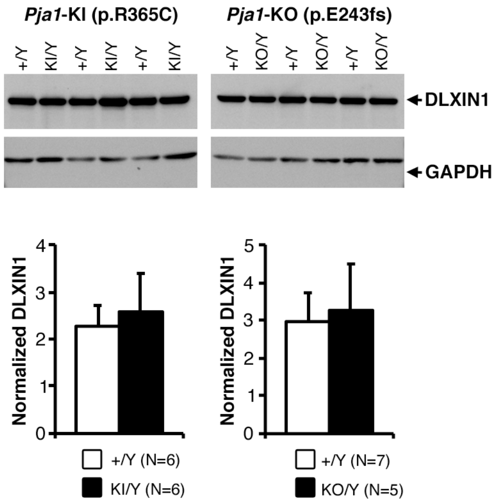

Supplementary Figure 5 l DLXIN1 protein amount is not significantly changed in brains of *Pja1*$^{KI/Y}$ and *Pja1*$^{KO/Y}$ mice. Expression level of DLXIN1 in cortical extracts from 9 weeks old *Pja1*$^{KI/Y}$ and *Pja1*$^{KO/Y}$ mice was mildly increased, although not significantly, compared to their respective WT littermates (*Pja1*-KI: WT = 6, *Pja1*$^{KI/Y}$ = 6; *Pja1*-KO: WT = 7, *Pja1*$^{KO/Y}$ = 5). Values are expressed as mean ± standard error of the mean.

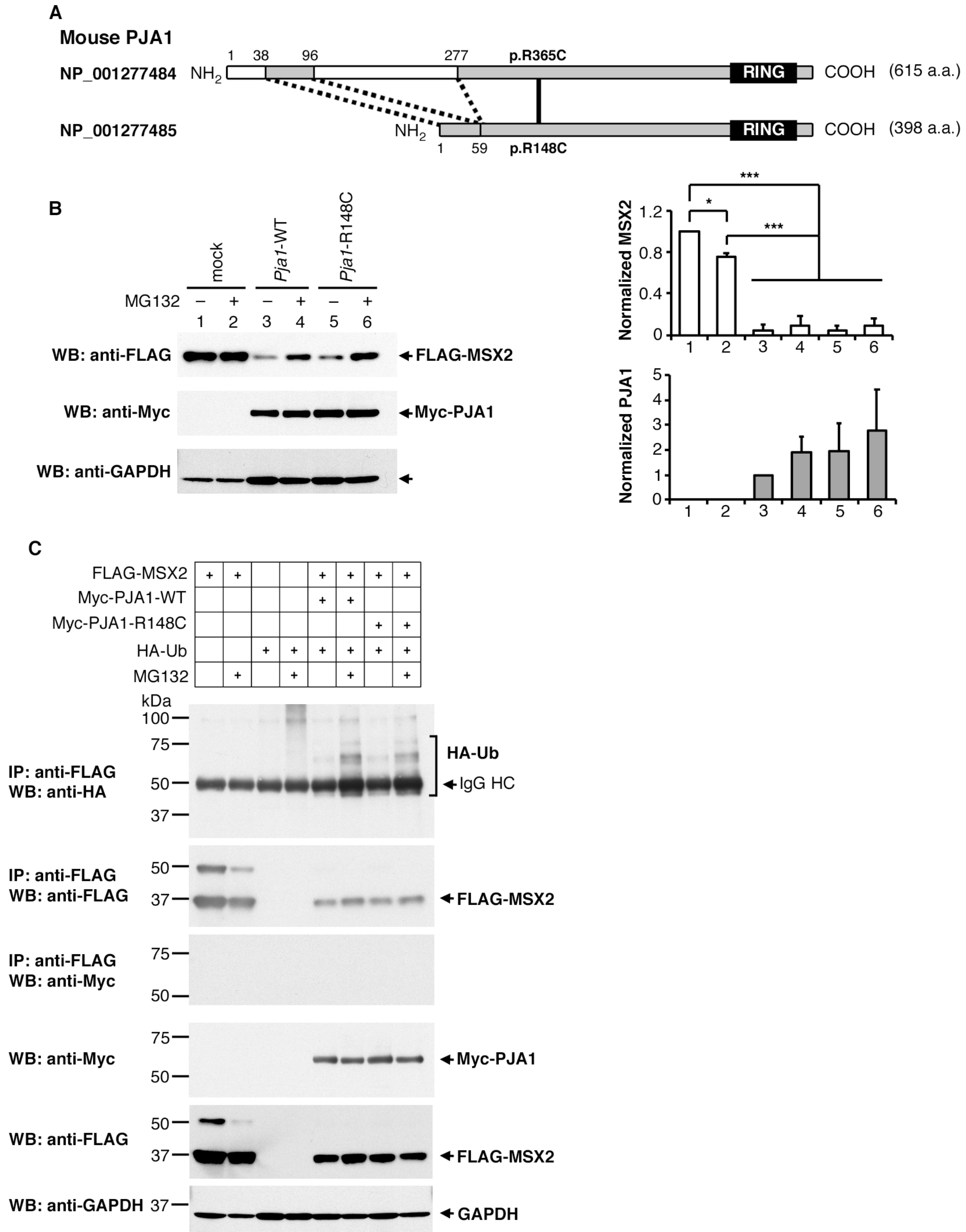

Supplementary Figure 6 | The R148C variant in PJA1 does not affect proteasome-mediated degradation of MSX2. (**A**) A c.442C>T variant (p.R148C), equivalent to the p.R365C variant in the long isoform of PJA1 (NP_001277484), was introduced into a Myc-tagged PJA1 short isoform (NP_001277485) expression construct. (**B**) Co-transfection of FLAG-MSX2 and Myc-PJA1 constructs into HEK293T cells revealed a comparable decrease in MSX2 protein for both the wild-type (WT) or mutant *Pja1* alleles. This drop in MSX2 protein was suppressed by treating the culture with the proteasome inhibitor MG-132, but the MSX2 amount in the presence of PJA1 and MG132 was still lower than that in the absence of PJA1. Experiments were made in triplicates parallelly. (**C**) Anti-FLAG immunoprecipitation followed by anti-HA western blot to reveal ubiquitinated proteins revealed bands corresponding to polyubiquitinated MSX2 that were only seen in the presence of PJA1 and amplified by proteasome inhibitors (upper panel). Anti-FLAG western blot against FLAG-MSX2 confirmed the presence of MSX2 protein, whereas anti-Myc western blot against Myc-PJA1 did not show any signal (second and third panels), indicating that MSX2 and PJA1 did not directly bind to each other as previously suggested[30]. MSX2 ubiquitination pattern was similar in the WT and mutant PJA1 constructs. PJA1 protein was however observed in the samples pre-immunoprecipitation (three lower panels), confirming proper expression of the vector. Values are expressed as mean ± standard error of the mean. One-way ANOVA and Tukey-Kramer post-hoc tests with significance set at (*) p<0.05 and (***) p<0.001.

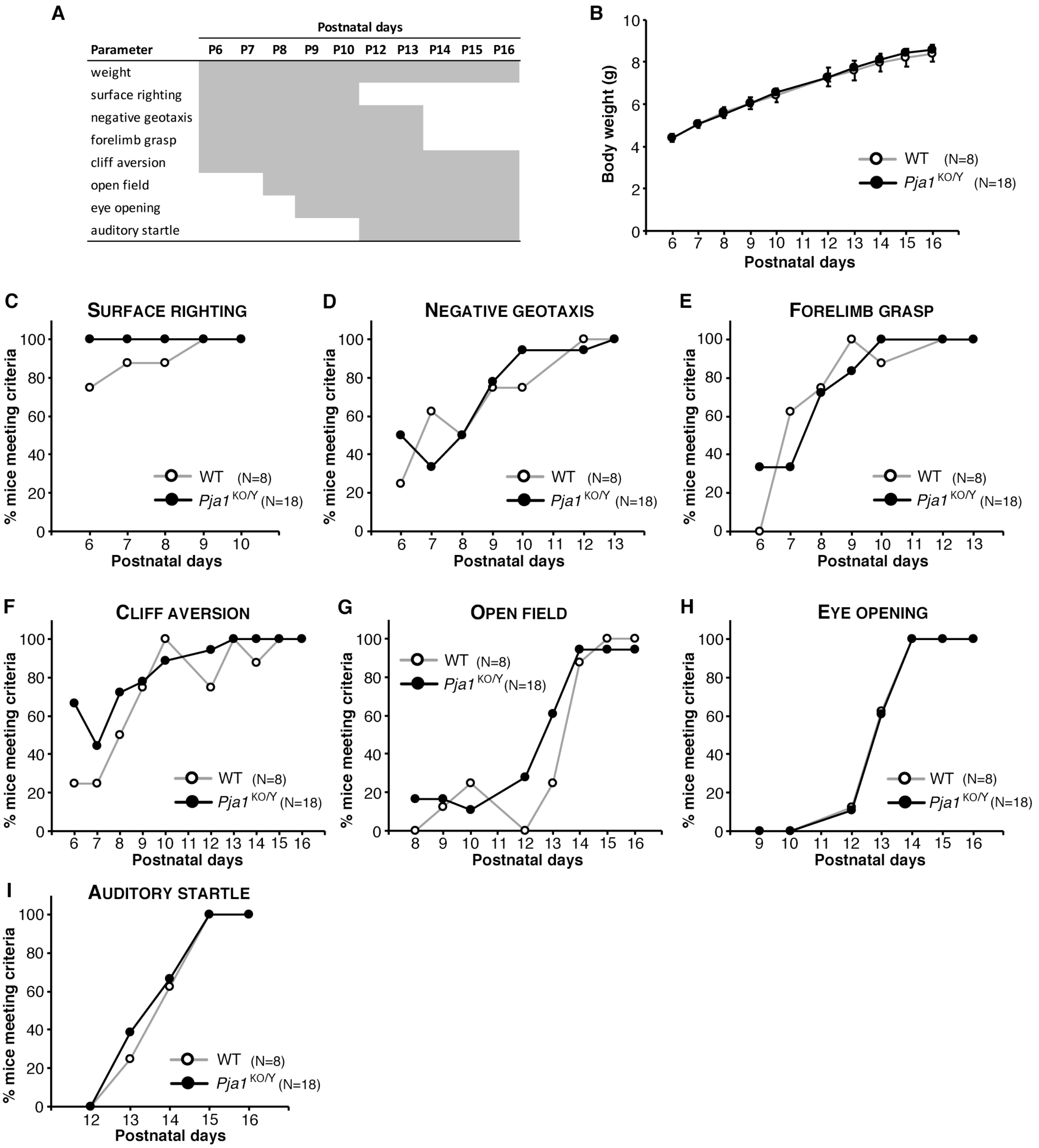

Supplementary Figure 7 I Unaffected developmental milestones in *Pja1*$^{KO/Y}$ mice. (**A**) Standard developmental milestones in *Pja1*$^{KO/Y}$ mice were assessed from postnatal day 6 through 16 (P6-P16) using a series of behavioral tasks. (**B**) The bodyweight increased gradually without showing significant differences between WT and *Pja1*$^{KO/Y}$ mice. Early postnatal milestones surface righting (**C**), negative geotaxis (**D**), forelimb grasp (**E**) and cliff aversion (**F**) were acquired by postnatal day 10 and no significant differences were seen across the groups. Later postnatal landmarks such as open field crossing (**G**), eyelid opening (**H**) and auditory startle (**I**) were also acquired by P14-P15 without significant differences between WT and *Pja1*$^{KO/Y}$ mice. Overall, postnatal neuromotor development was conserved in *Pja1*$^{KO/Y}$ juvenile mice. Values in (B) are expressed as mean ± standard error of the mean. Values in (C-I) represent the percentage of mice succeeding the task on a given day, statistical significance was assessed in regards to the time necessary to complete the task with the maximum cutoff time given to animals that failed it. Two-way ANOVA with significance set at $p<0.05$.

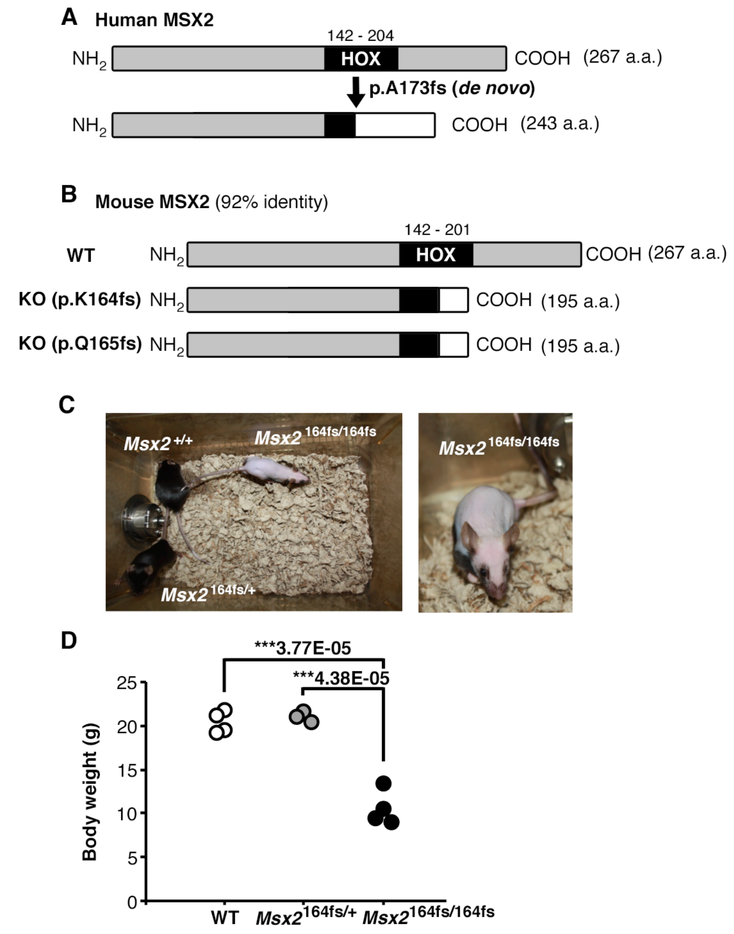

Supplementary Figure 8 | Modelling of the *MSX2 de novo* p.A173fs frameshift variant found in a patient of NDD recapitulates knockout phenotype in mice. (**A**) The p.A173fs frameshift variant leads in human to a truncated form of MSX2 and affects the homeobox domain of the protein. (**B**) The structure of MSX2 protein is highly conserved between human and mouse, and equivalent variants were introduced by CRISPR-Cas9 targeted mutagenesis, creating two mouse lines, *Msx2*$^{164fs/+}$ and *Msx2*$^{165fs/+}$ with MSX2 frameshift variants: p.K164fs and p.Q165fs, respectively. Homozygotes of both lines showed a similar phenotype with the absence of fur on the entirety of the body except for patches around the nose (**C**) and a significantly lower bodyweight at four weeks old (**D**) in *Msx2*$^{164fs/164fs}$ mice (data not shown for *Msx2*$^{165fs/165fs}$ mice). One-way ANOVA and Tukey-Kramer post-hoc tests with significance set at (***) $p<0.001$.

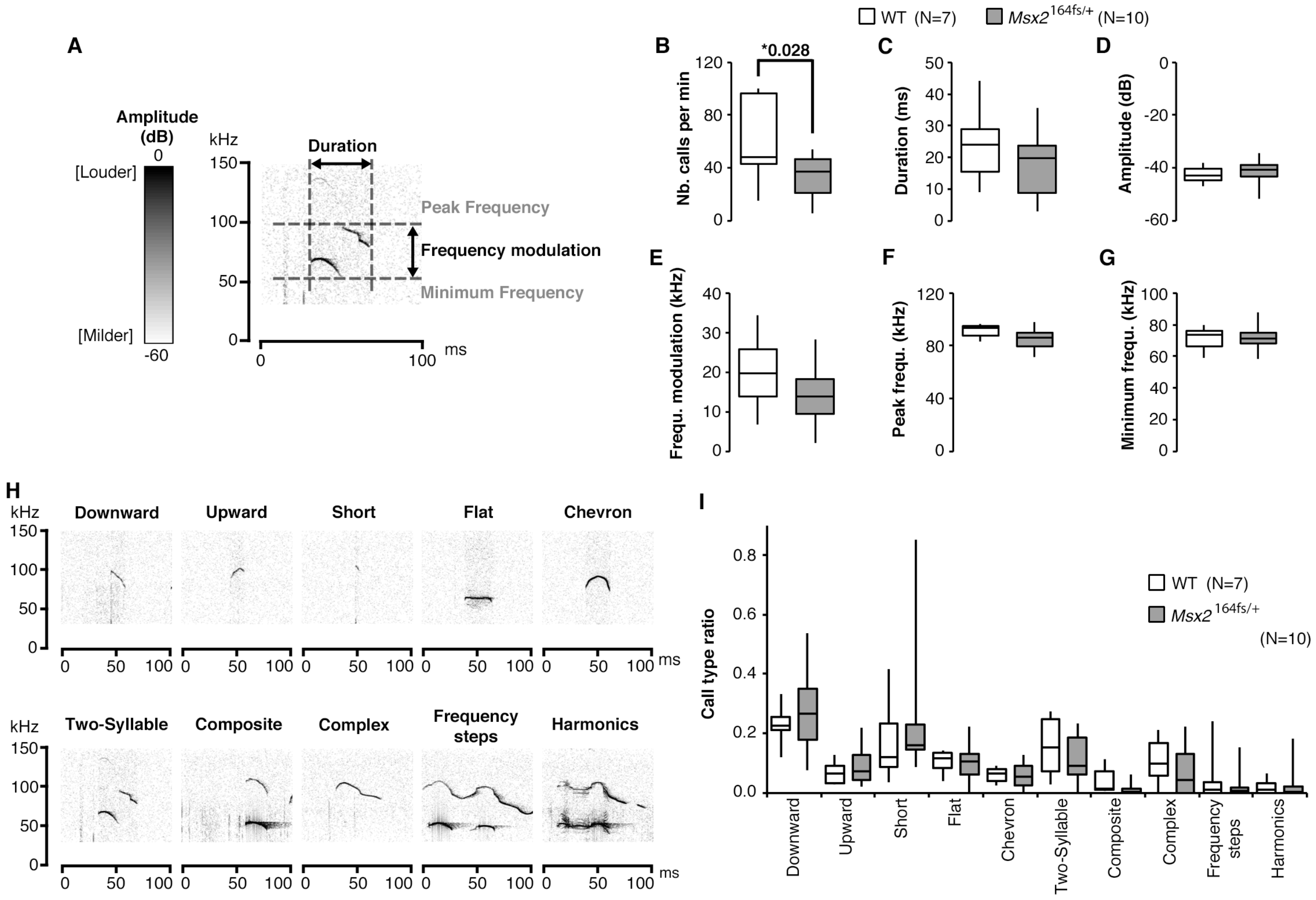

Supplementary Figure 9 I Decreased isolation-induced vocalizations in *Msx2*$^{164fs/+}$ pups without changes in call properties or vocalization repertoire. (**A**) Isolation induced ultrasonic vocalizations (USV) produced by pups at postnatal day 6 were analyzed for intrinsic parameters relative to their amplitude, duration and frequency. (**B**) The number of calls produced by *Msx2*$^{164fs/+}$ pups was significantly lower than their WT littermates. The average call duration (**C**) was slightly, yet not significantly, shorter in the *Msx2*$^{164fs/+}$ group whereas the average amplitude (**D**) was not significantly changed. The frequency modulation (**E**) of USV calls was mildly decreased but this change did not reach the significance level. The peak frequency (**F**) and minimum frequency (**G**) were not significantly affected in *Msx2*$^{164fs/+}$ pups. (**H**) All calls were classified into ten major categories commonly observed in C57BL/6 pups. Examples displayed were extracted from randomly chosen tracks from the WT group. (**I**) The repertoire produced by *Msx2*$^{164fs/+}$ pups did not differ significantly from that of their WT littermates. One-way ANOVA with significance set at (*) $p<0.05$.

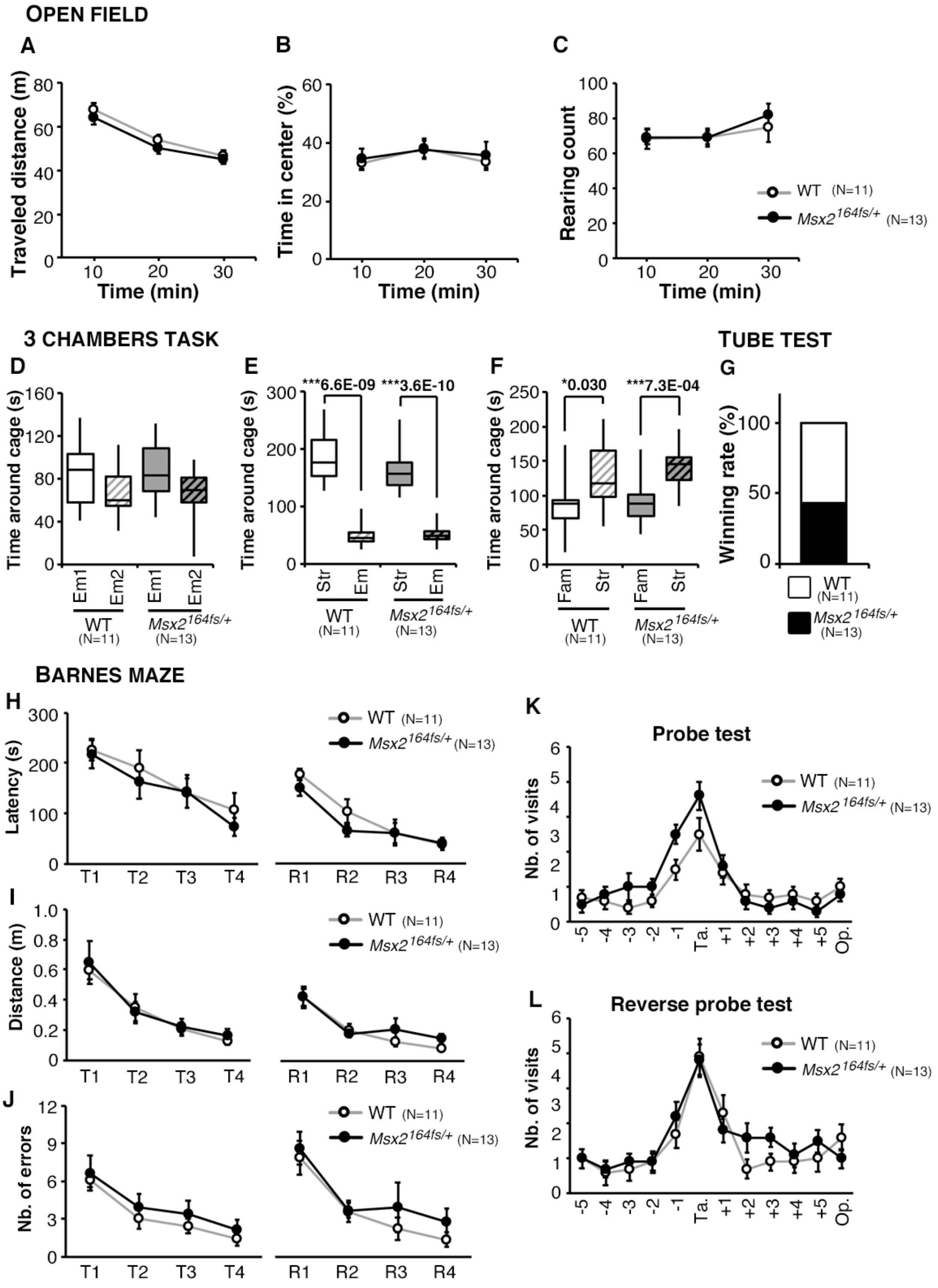

Supplementary Figure 10 I Conserved exploratory behavior, social behavior and spatial learning/memory in *Msx2*$^{164fs/+}$ mice. In the open field task, *Msx2*$^{164fs/+}$ mice did not show significant differences compared to WT littermates in terms of travelled distance (**A**), time spent in the central area (**B**) or number of rearing (**C**) during the 30 minutes of the task. In the 3 chambers task, while no preference for a specific side was seen in the habituation period (**D**), mice from both groups spent significantly more time investigating the stranger's side than the empty side during the sociability phase of the task (**E**). Mice from both groups significantly spent more time on the stranger mice than the familiar one suggesting that preference for social novelty was not altered in *Msx2*$^{164fs/+}$ animals (**F**). (**G**) In the tube test, the wining rate of *Msx2*$^{164fs/+}$ opposed to their WT littermates did not differ significantly from the 50% random outcome. In the Barnes maze task, spatial learning was conserved in *Msx2*$^{164fs/+}$ mice as they showed a similar latency (**H**), distance travelled (**I**) and number of errors (**J**) to reach the target hole during the primary learning as well as the reverse learning periods. In the probe test (**K**) as well as in the reverse probe test (**L**) the performance of *Msx2*$^{164fs/+}$ mice was not significantly different from that of their WT littermates. Values in (A-C) and (H-L) are expressed as mean ± standard error of the mean. Two-way ANOVA (A-C, H-L), Chi-square test (G) or one-way ANOVA (D-F) with significance set at (*) $p<0.05$ and (***) $p<0.01$.

Patients information

NP089

A 13-year old female with autism spectrum disorder.

NP149

A 37-year-old female with autism spectrum disorder and attention-deficit hyperactivity disorder. A delay in verbal and motor development was noted in her early childhood. She had a difficulties communicating and interacting with people. She was also easily distracted and had troubles organizing tasks and activities. She was extremely sensitive to sounds.

NP167

A 15-year-old male with autism spectrum disorder and intellectual disability. A delay in verbal development was noted in his early childhood. He exhibited an unusual interest in numbers and had problems with social interaction and communication. He easily got excited, had difficulties staying seated and displayed head-banging behavior. Psychological assessment confirmed mild intellectual disability.

OKI-005-1

A 4-year-old male when diagnosed with mild trigonocephaly with clinical symptoms. He had speech delay with a vocabulary of only a few words, presented difficulties in understanding, poor eye contacts and was unable to play with other children. He was hyperactive and was reported to be head banging and to have terrors at night every day. Three-dimensional computed tomography (3D-CT) revealed the presence of a metopic ridge with a narrow frontal area of the skull, anterior fossa and pronounced digital markings that led to a suspicion of intracranial pressure. No particular abnormality was found by MRI, apart from

the relatively small size of the frontal lobes. He underwent a decompressive cranioplasty resulting in an enlargement of the frontal area of the skull. Post-operation 3D-CT and MRI showed an increase in the size of the anterior fossa and the frontal lobes. One year later eye contacts, social play with other children and vocabulary improved, hyperactivity decreased and head banging and terrors at night ceased. The patient is now aged eleven and attends school in a special support class. He is able to speak long sentences, though slowly, and to engage in social play with other children.

OKI-005-2

The younger brother of OKI-005-1, 2-year-old at the diagnosis. He was hyperactive and had a delay in language acquisition with a vocabulary of only a few words associated with poor understanding performance. He developed poor eye contact, was described as unable to play with other children and like his brother displayed head banging behavior. He was diagnosed with trigonocephaly with a metopic ridge identified by 3D-CT and MRI showed smaller frontal lobes. He underwent decompressive cranioplasty that had the same effect than in patient OKI-005-1: enlargement of the frontal area of the skull and of the frontal lobes. A few months post-surgery head banging had ceased and hyperactivity tended to decrease. Language acquisition started to improve after a year and two-years post-surgery he was able to produce two-word sentences. He was also able to engage in social play with other children. At present, he is eight years old and attends school in a special support class, is able to use long sentences and to engage in conversations.

OKI-011-1

The 4-year old sister of OKI-011-2. She had a speech acquisition delay (unable to speak more than two-words sentences) and had poor understanding abilities especially for dealing with

numbers. She was somewhat hyperactive and showed difficulties to play with other children, including her brothers. Her developmental quotient (DQ) was estimated to 83 (below average). She was diagnosed with trigonocephaly with a metopic ridge identified by 3D-CT and MRI showed smaller frontal lobes. In a six months follow-up observation no changes were seen. Parents decided a decompressive craniotomy as she turned five. Significant progress was observed following the surgery: within a month she started to produce three-words sentences and started making conversations, her understanding also improving. After six months, her teacher at nursing school reported she had no problems in daily activities. Her DQ reached 103. She entered a regular elementary school. At present, she is in a regular high school without problems reported.

OKI-011-2

The 2-year old brother of OKI-011-1. He had been developing normally up to two years old. His DQ was 104, he could play with other children and was able to report to his parents what he had done during the day. From two years old a deterioration was observed: he was losing words and had been unable to call his friends' names. He started to call everybody using the same name. He was easily excited and started shouting non-understandable words. He was described to have a strong commitment and to easily fall while walking. His DQ estimation was 84 at when he turned three. He was diagnosed with trigonocephaly with a metopic ridge identified by 3D-CT and MRI showed smaller frontal lobes. He underwent a decompressive cranioplasty at four. After surgery he showed clear signs of progress. In one month, he became able to express emotions such as scare of darkness. His hyperactivity decreased. His friend described him as being more tender. No more regression was observed. His strong commitment improved. In two years, he was able to complete every activity proposed at his nursery school. He learned very quickly and was curious about everything. He entered a

regular elementary school without problems. At present, he attends high school and has a normal daily life.

OKI-020-1

A 4-year-old male diagnosed with trigonocephaly associated with speech delay (with a vocabulary of less than ten word) and hyperactivity. He was also reported to have poor eye contact, strong commitment to foods and toys, to be unable to play with other children and was not toilet trained. He displayed signs of sleep disorders: difficulties to fall asleep, waking up at night to get some food. He underwent a decompressive cranioplasty. A few months later, his vocabulary and understanding capabilities improved while his hyperactivity partly resolved and his sleeping problems disappeared. He also became toilet trained. One year post-surgery he was able to engage in social play, hyperactivity mostly disappeared and the persistence-like behavior was not seen anymore. He is now ten years old and is able to speak 6 words sentences. His IQ is in the low range and he attends school in a special support class.

OKI-020-4

The 11-year-old elder brother of OKI-020-1 at the diagnosis. He was diagnosed with a mild trigonocephaly associated with hyperactivity and a delay in language acquisition. No decompressive surgery was done as other associated symptoms were mild. He attends school in a special support class due to cognitive disability. His progression is described as slow. Hyperactivity is still present but became milder than at the first diagnosis.

OKI-061-1

A 3-year-old male diagnosed with a mild trigonocephaly. He had a speech learning delay and developed a vocabulary of about 20 words. He was hyperactive, had poor eye contact,

difficulties to understand rules, showed a strong commitment to his miniature cars. He had difficulties sleeping at night and was reported to have an unbalanced diet. He underwent a decompressive cranioplasty that led to an enlargement of the frontal area of the skull and an increase in volume of the frontal lobes of the brain. A few months later he was able to converse using sentences and his other symptoms improved. He is now five years old and attends a standard kindergarten.

RUM062

A 4-year-old male with autism spectrum disorder and intellectual disability. He had poor eye contact, little interest in other people, and was hyperactive. A delay in verbal development was noted at two years of age. He attended rehabilitation courses in children’s hospitals. Repetitive use of language and hypersensitivity to smell were observed.

SIZ-894

A 21-year-old male with a history of language developmental delay. He had a cranial meningocele that necessitated a surgery three days after birth. He had two episodes of convulsive seizure at 21. EEG showed right frontotemporal dominant spikes, polyspikes with slow waves. MRI was normal. There was a family history of epilepsy (a cousin).

SIZ-897

A 30-year-old male with autism spectrum disorder and intellectual disability. Convulsive seizures started at 12 years of age and occurred about once a year. From the age of 26, monthly seizures with automatism appeared and were preceded by epigastric discomfort. Seizures were pharmacoresistant. EEG showed spikes in the temporal region on the right side whereas MRI was normal. There was no family history of epilepsy.

SIZ-978

A 32-year-old female, with cerebral palsy and severe intellectual disability. An aunt had a history of epilepsy. The patient had a febrile convulsive seizure when she was eight months old. The epilepsy appeared at 17 months with head nodding. She was diagnosed with West syndrome and later Lennox-Gastaut syndrome with frequent tonic and atypical absence seizures. The seizures are medically intractable.

Antibodies against Msx2 yielded non-specific signals

The *Msx2*$^{164fs/164fs}$ mice created in the present study displayed characteristic phenotypes (Supplementary Figure 8) similar to a previous report[43]. We have confirmed the insertion and the frameshift in *Msx2* by sequencing genomic DNA and RNA. On a Western blot however all of the antibodies we tested revealed bands at ~29 KDa that were seen in brain extracts from wild-type as well as homozygous *Msx2*$^{164fs/164fs}$ carriers with a comparable intensity, suggesting a non-specific labeling (data not shown). List of antibodies tested: anti-MSX2 mouse monoclonal antibody (M04, clone 1F6, H00004488-M04, Abnova), anti-MSX2/HOX8 rabbit polyclonal antibody (NBP1-85445, NOVUS), anti-MSX2 rabbit polyclonal antibody (AB10211, MILLIPORE), anti-MSX2 mouse monoclonal antibody (B-2, sc-3939869, Santa Cruz Biotechnology) and anti-MSX2 goat polyclonal antibody (N-20, sc-17729, Santa Cruz Biotechnology).

SUPPLEMENTARY METHODS

Haplotype analysis

We designed PCR primers to amplify 21 selected single nucleotide polymorphisms (SNPs) markers flanking the p.R376C mutation in *PJA1* (rs745369655). SNPs were selected for high minor allele frequency in 1,000 genomes browser. Genomic DNA derived from the 7 patients with the p.R376C mutation was amplified by PCR using PrimeSTAR HS DNA Polymerase (TaKaRa) or KOD-plus Ver. 2 (Toyobo). Primer sequences and PCR conditions are available upon request. The PCR products were purified using ExoSAP-IT PCR product Cleanup (Affymetrix) and analyzed by direct sequencing using an ABI PRISM 3730xl Genetic Analyzer.

Skull morphology

Mice, aged 5 months old, received a lethal dose of avertin and decapitated. Skin and underlying tissue were removed and the skull placed in 70% ethanol for two weeks. Remaining tissue was cleaned off and pictures acquired. Morphometric measures were taken using a precision caliper directly from the skull, using specific landmarks at the interconnexion of the main sutures (Supplementary Figure 2). Data was acquired from 5 WT and 5 *Pja1*$^{KI/+}$ or 6 WT and 6 *Pja1*$^{KO/+}$animals.

Expression constructs and mutagenesis

The following expression constructs were used: Myc-Pja1 (2~398a.a., NP_001277485), FLAG-Msx2 (2~267 a.a., NP_038629) and 3xHA-Ubiquitin (153~228 a.a., NP_001300913). These constructs were reported previously.[30] We introduced a c.442C>T mutation (p.R148C), equivalent to the p.R376C mutation in human for this isoform, using the QuickChange Site-Directed Mutagenesis kit (Agilent Technologies) and the following oligonucleotides (Fw: cctgTgcaggaagtaccgaagccgagagc and rev: ctgcAcagggcagccatccactctttgtcg). Effective

nucleotide change and integrity of the rest of the ORF sequence were confirmed by DNA sequencing.

Protein stability and ubiquitination in cultured cells

HEK293T cells were transfected with plasmids with or without MG-132 treatment (1 μM, SIGMA-ALDRICH). 24 h after transfection, cells were homogenized in lysis buffer (10 mM Tris-HCl, 150 mM NaCl, 5 mM EDTA, 1% TritonX-100, 1% sodium deoxycholate, 0.1% SDS). Sample lysates were separated on 5-20% gradient SDS-polyacrylamide gel (Super Sep Ace, Wako pure reagents) and immunoblotted. To analyze protein ubiquitination, we performed immunoprecipitation: 24 h after transfection, lysis was done by incubating cells in 200 μl 2% SDS in TBS (20 mM Tris-HCl, pH 7.5, 150 mM NaCl) at 100 °C for 10 min. After addition of 800 μl 1% Triton-X- 100 in TBS lysates were sonicated. The lysates were subjected to immunoprecipitation of FLAG-tagged MSX2 using anti-FLAG M2 Agarose Affinity Gel (A2220, SIGMA-ALDRICH), separated on 5-20% gradient SDS-polyacrylamide gel and performed Western blotting to detect ubiquitinated MSX2 proteins. Anti-FLAG mouse monoclonal M2-Peroxidase antibody (1:4,000 dilution, A8592, SIGMA-ALDRICH), anti-HA rat monoclonal antibody (1:1,000 dilution, 3F10, 11867423001, Roche) or anti-Myc-tag rabbit polyclonal antibody (1:1,000 dilution, 2272, Cell Signaling Technology) were used. Secondary antibodies were horseradish peroxidase (HRP) conjugated anti-rabbit IgG antibody (1:10,000 Jackson Immuno Research) or HRP conjugated anti-rat IgG antibody (1:1,000 dilution, sc-2006, Santa Cruz Biotechnology).

To check the expression of DLXIN1 protein, prefrontal cortex was collected from 6 weeks old mice (N=6 WT and 6 *Pja1*$^{KI/Y}$ and N=7 WT and 5 *Pja1*$^{KO/Y}$) and flash frozen in liquid nitrogen. Samples were homogenized in ice-cold lysis buffer (10 mM PIPES pH6.8, 100 mM NaCl, 300 mM Sucrose, 3 mM $MgCl_2$, 1 mM EDTA, 0.5% Triton-X 100) supplemented with protease inhibitors (Complete). Homogenates were centrifuged at 20,000g

for 15min., separated on 5-20% gradient SDS-polyacrylamide gel and performed Western blotting. The anti-DLXIN1 rabbit polyclonal antibody (1:1,000 dilution, 74-112, Bio Academia) was used.

Postnatal developmental milestones

Pups were tested from postnatal day 6 and weighted after every day's testing session. Pups, housed with the mother, were transferred to the testing rooms 1 hour before start and every pup went through the testing battery and was then return to the home cage.

Surface righting. The pup was gently placed on its back on top of a smooth plastic sheet. The time necessary for the pup to flip on its paws was measured with a cutoff of 30 seconds from which pups were considered to have failed the task.

Negative Geotaxis. Pups were placed head-down on a metallic grid inclined at 45° and the latency to spin 180° to reach an upward position was measured, with a cutoff of 30 seconds from which pups were considered to have failed the task.

Forelimb grasp. Pups were presented with the front paws facing a 3 mm diameter wire placed 5 cm above bedding chips and was released as soon as it grasped the wire. The pup was considered to have succeeded the task if it was able to hang for at least one second. A second trial was given if the pup fell off immediately upon release.

Cliff aversion. Pups were place at the edge of an 8 cm high plastic box with the forepaws partially sticking out of the ledge. The latency to step back and/or turn away from the ledge was measured with a cutoff of 30 seconds from which pups were considered to have failed the task.

Open field. Pups were placed on top of a smooth plastic sheet at the center of a 13 cm diameter circle. The time necessary to walk out of the circle (i.e. all four paws entirely outside of the circle) was recorded with a cutoff of 30 seconds from which pups were considered to have failed the task.

Eye opening. Pups were inspected daily from postnatal day 8 to determine the first day when both eyes opened.

Auditory startle. Pups were placed on the testing bench and their reaction to a handclap at ~10 cm was recorded. Pups were considered to have succeeded the test if they responded by an involuntary jump.
