# Supplementary material for: A recurrent PJA1 variant in trigonocephaly and neurodevelopmental disorders
Source: Ann Clin Transl Neurol. 2020 Jun 12;7(7):1117–31. doi: 10.1002/acn3.51093 (PMC7359110; doi:10.1002/acn3.51093)
Supplement: Supplementary file 2 — Table S1. 95 NDD patients, their parents and associated phenotypes for whole‐exome sequencing. Table S2. Number of NDD patients per associated phenotypes and analysis. Table S3. 463 NDD patients, their parents and associated phenotypes for targeted sequencing Table S4. De novo mutations identified by whole‐exome sequencing. Table S5. Hemizygous mutations identified by whole‐exome sequencing. Table S6. Homozygous mutations identified by whole‐exome sequencing. Table S7. Compound heterozygous mutations identified by whole‐exome sequencing. Table S8. PJA1 and MSX2 nonsynonymous mutations and phenotypic features in individuals with the mutations. Table S9. Allele frequency of PJA1 p.Arg376Cys mutation in various populations. [file ACN3-7-1117-s002.pdf]

Sample ID	Diagnosis	Note	Trigonocephaly	Epilepsy	Gender
OKI-001-1	Delay in speech acquisition, Hyperkinesia, ASD, Sleep disorders, Trigonocephaly	Patient	+	Unknown	M
OKI-001-2		Father			M
OKI-001-3		Mother			F
OKI-002-1	Delay in speech acquisition, Hyperkinesia, ASD, Regression, Trigonocephaly	Patient	+	Unknown	M
OKI-002-2		Father			M
OKI-002-3		Mother			F
OKI-003-1	Delay in speech acquisition, Hyperkinesia, ASD, Regression, Sleep disorders, Self-injurious behavior, Trigonocephaly	Patient	+	Unknown	M
OKI-003-2		Father			M
OKI-003-3		Mother			F
OKI-004-1	Delay in speech acquisition, Hyperkinesia, ASD, Self-injurious behavior, Panic disorders, Sleep disorders, Trigonocephaly	Patient	+	Unknown	M
OKI-004-2		Father			M
OKI-004-3		Mother			F
OKI-005-1	Delay in speech acquisition, Hyperkinesia, ASD, Self-injurious behavior,Panic disorders,Trigonocephaly	Patient	+	-	M
OKI-005-2	Delay in speech acquisition, Self-injurious behavior, Trigonocephaly	Patient	+	-	M
OKI-005-3		Father			M
OKI-005-4		Mother			F
OKI-006-1	Motor developmental delay, Trigonocephaly	Patient	+	Unknown	F
OKI-006-2		Father			M
OKI-006-3		Mother			F
OKI-007-1	Delay in speech acquisition, Motor developmental delay, Hyperkinesia, ASD, Trigonocephaly	Patient	+	Unknown	M
OKI-007-2		Father			M
OKI-007-3		Mother			F
OKI-008-1	Delay in speech acquisition, Hyperkinesia, ASD, Sleep disorders, Trigonocephaly	Patient	+	Unknown	F
OKI-008-2		Father			M
OKI-008-3		Mother			F
OKI-009-1	Delay in speech acquisition, Motor developmental delay, Trigonocephaly	Patient	+	Unknown	F
OKI-009-2		Father			M
OKI-009-3		Mother			F
OKI-010-1	Delay in speech acquisition, Motor developmental delay, Hyperkinesia, ASD, Trigonocephaly	Patient	+	Unknown	M
OKI-010-2		Father			M
OKI-010-3		Mother			F
OKI-011-1	Delay in speech acquisition, ASD, Panic disorders, Trigonocephaly	Patient	+	-	F
OKI-011-2	Delay in speech acquisition, Motor developmental delay, Regression, Panic disorders, Trigonocephaly	Patient	+	Unknown	M
OKI-011-3		Father			M
OKI-011-4		Mother			F

OKI-012-1	Delay in speech acquisition, Hyperkinesia, ASD, Panic disorders, Trigonocephaly	Patient	+	Unknown	M
OKI-012-2		Father			M
OKI-012-3		Mother			F
OKI-013-1	Delay in speech acquisition, Hyperkinesia, ASD, Sleep disorders, Trigonocephaly	Patient	+	Unknown	M
OKI-013-2		Father			M
OKI-013-3		Mother			F
OKI-014-1	Delay in speech acquisition, Hyperkinesia, Trigonocephaly	Patient	+	Unknown	M
OKI-014-2		Father			M
OKI-014-3		Mother			F
OKI-015-1	Delay in speech acquisition, Motor developmental delay, Hyperkinesia, Trigonocephaly	Patient	+	Unknown	M
OKI-015-2		Father			M
OKI-015-3		Mother			F
OKI-016-1	Delay in speech acquisition, Hyperkinesia, ASD, Regression, Self-injurious behavior, Sleep disorders, Trigonocephaly	Patient	+	Unknown	F
OKI-016-2		Father			M
OKI-016-3		Mother			F
OKI-017-1	Delay in speech acquisition, Hyperkinesia, ASD, Regression, Panic disorders, Trigonocephaly	Patient	+	Unknown	M
OKI-017-2		Father			M
OKI-017-3		Mother			F
OKI-019-1	Delay in speech acquisition, Hyperkinesia, ASD, Grand mal seizure, Trigonocephaly	Patient	+	Unknown	M
OKI-019-2		Father			M
OKI-019-3		Mother			F
OKI-020-1	Delay in speech acquisition, Hyperkinesia, ASD, Panic disorders, Trigonocephaly	Patient	+	-	M
OKI-020-4	Delay in speech acquisition, Hyperkinesia, Trigonocephaly	Patient	+	-	M
OKI-020-2		Father			M
OKI-020-3		Mother			F
OKI-021-1	Delay in speech acquisition, Hyperkinesia, Trigonocephaly	Patient	+	Unknown	M
OKI-021-2		Father			M
OKI-021-3		Mother			F
OKI-022-1	Delay in speech acquisition, Motor developmental delay, Trigonocephaly	Patient	+	Unknown	F
OKI-022-2		Father			M
OKI-022-3		Mother			F
OKI-024-1	Delay in speech acquisition, Trigonocephaly	Patient	+	Unknown	M
OKI-024-2		Father			M
OKI-024-3		Mother			F
OKI-025-1	Delay in speech acquisition, Motor developmental delay, Sleep disorders, Trigonocephaly	Patient	+	Unknown	M
OKI-025-2		Father			M
OKI-025-3		Mother			F
OKI-026-1	Delay in speech acquisition, Hyperkinesia, Trigonocephaly	Patient	+	Unknown	M
OKI-026-2		Father			M

OKI-026-3		Mother			F
OKI-027-1	Delay in speech acquisition, Motor developmental delay, Hyperkinesia, ASD, Sleep disorders, Trigonocephaly	Patient	+	Unknown	M
OKI-027-2		Father			M
OKI-027-3		Mother			F
OKI-028-1	Delay in speech acquisition, Hyperkinesia, Sleep disorders, Trigonocephaly	Patient	+	Unknown	M
OKI-028-2		Father			M
OKI-028-3		Mother			F
OKI-029-1	Delay in speech acquisition, Hyperkinesia, ASD, Trigonocephaly	Patient	+	Unknown	F
OKI-029-2		Father			M
OKI-029-3		Mother			F
OKI-030-1	Delay in speech acquisition, Motor developmental delay, ASD, Sleep disorders, Trigonocephaly	Patient	+	Unknown	F
OKI-030-2		Father			M
OKI-030-3		Mother			F
OKI-031-1	Delay in speech acquisition, Hyperkinesia, ASD, Self-injurious behavior, Sleep disorders, Trigonocephaly	Patient	+	Unknown	F
OKI-031-2		Father			M
OKI-031-3		Mother			F
OKI-032-1	Delay in speech acquisition, Motor developmental delay, Hyperkinesia, ASD, Self-injurious behavior, Trigonocephaly	Patient	+	Unknown	M
OKI-032-2		Father			M
OKI-032-3		Mother			F
OKI-034-1	Delay in speech acquisition, Hyperkinesia, Self-injurious behavior, Panic disorders, Sleep disorders, Trigonocephaly	Patient	+	Unknown	M
OKI-034-4	Delay in speech acquisition, Hyperkinesia, Self-injurious behavior, Panic disorders, Trigonocephaly	Patient	+	Unknown	M
OKI-034-2		Father			M
OKI-034-3		Mother			F
OKI-035-1	Delay in speech acquisition, Motor developmental delay, Trigonocephaly	Patient	+	Unknown	F
OKI-035-2		Father			M
OKI-035-3		Mother			F
OKI-036-1	Delay in speech acquisition, Hyperkinesia, Self-injurious behavior, Panic disorders, Sleep disorders, Trigonocephaly	Patient	+	Unknown	F
OKI-036-2		Father			M
OKI-036-3		Mother			F
OKI-037-1	Delay in speech acquisition, Motor developmental delay, Hyperkinesia, ASD, Trigonocephaly	Patient	+	Unknown	F
OKI-037-2		Father			M
OKI-037-3		Mother			F
OKI-038-1	Delay in speech acquisition, Motor developmental delay, Trigonocephaly	Patient	+	Unknown	F
OKI-038-2		Father			M
OKI-038-3		Mother			F
OKI-039-1	Delay in speech acquisition, Hyperkinesia, ASD, Self-injurious behavior,Panic disorders,Trigonocephaly	Patient	+	Unknown	M

OKI-039-2		Father			M
OKI-039-3		Mother			F
OKI-040-1	Delay in speech acquisition, Hyperkinesia, Self-injurious behavior, Panic disorders, Trigonocephaly	Patient	+	Unknown	M
OKI-040-2		Father			M
OKI-040-3		Mother			F
OKI-041-1	Delay in speech acquisition, Hyperkinesia, ASD, Trigonocephaly	Patient	+	Unknown	M
OKI-041-2		Father			M
OKI-041-3		Mother			F
OKI-042-1	Delay in speech acquisition, Hyperkinesia, ASD, Trigonocephaly	Patient	+	-	M
OKI-042-2		Father			M
OKI-042-3		Mother			F
OKI-043-1	Delay in speech acquisition, Motor developmental delay, Hyperkinesia, ASD, Trigonocephaly	Patient	+	Unknown	M
OKI-043-2		Father			M
OKI-043-3		Mother			F
OKI-044-1	Delay in speech acquisition, Motor developmental delay, Hyperkinesia, ASD, Trigonocephaly	Patient	+	Unknown	M
OKI-044-2		Father			M
OKI-044-3		Mother			F
OKI-045-1	Delay in speech acquisition, Hyperkinesia, ASD, Trigonocephaly	Patient	+	Unknown	M
OKI-045-2		Father			M
OKI-045-3		Mother			F
OKI-046-1	Hyperkinesia, ASD, Trigonocephaly	Patient	+	Unknown	M
OKI-046-2		Father			M
OKI-046-3		Mother			F
OKI-047-1	Delay in speech acquisition, Motor developmental delay, Trigonocephaly	Patient	+	Unknown	M
OKI-047-2		Father			M
OKI-047-3		Mother			F
OKI-048-1	Delay in speech acquisition, Hyperkinesia, Trigonocephaly	Patient	+	Unknown	F
OKI-048-2		Father			M
OKI-048-3		Mother			F
OKI-050-1	Delay in speech acquisition, ASD, Trigonocephaly	Patient	+	Unknown	F
OKI-050-2		Father			M
OKI-050-3		Mother			F
OKI-052-1	Delay in speech acquisition, Hyperkinesia, ASD, Regression, Trigonocephaly	Patient	+	Unknown	M
OKI-052-2		Father			M
OKI-052-3		Mother			F
OKA-218	High-function ASD, abnormal EEG	Patient	Unknown	-	F
OKA-219	ASD, ID, Epilepsy	Patient	Unknown	+	M
OKA-216		Father			M
OKA-217		Mother			F
OKA-02-26	NDD, PE	Patient	Unknown	+	M
OKA-02-28		Father			M
OKA-02-40		Mother			F
OKA-224	ASD, Epilepsy	Patient	Unknown	+	F
OKA-225	ASD, Epilepsy	Patient	Unknown	+	M
OKA-222		Father			M
OKA-223		Mother			F

SIZ-204	SMEI (*SCN1A* mutation-negative) (moderate ID)	Patient	Unknown	+	F
SIZ-205		Father			M
SIZ-206		Mother			F
SIZ-244	Doose syndrome (DQ: 59)	Patient	Unknown	+	F
SIZ-245		Father			M
SIZ-246		Mother			F
SIZ-327	developmental delay, PE	Patient	Unknown	+	F
SIZ-328		Father			M
SIZ-329		Mother			F
SIZ-330	developmental delay, Doose syndrome	Patient	Unknown	+	M
SIZ-331		Father			M
SIZ-332		Mother			F
SIZ-335	developmental delay, SGE	Patient	Unknown	+	M
SIZ-336		Father			M
SIZ-337		Mother			F
SIZ-439	ID, ICEGTC closely related (6Y: IQ 59, 14Y: WISC IQ 62)	Patient	Unknown	+	M
SIZ-440		Father			M
SIZ-441		Mother			F
SIZ-503	moderate ID, PE	Patient	Unknown	+	F
SIZ-504		Father			M
SIZ-505		Mother			F
SIZ-552	FLE (IQ: 46)	Patient	Unknown	+	F
SIZ-553		Father			M
SIZ-554		Mother			F
SIZ-664	PE (DQ: 30)	Patient	Unknown	+	M
SIZ-665		Father			M
SIZ-666		Mother			F
SIZ-672	PE (DQ: 59)	Patient	Unknown	+	F
SIZ-673		Father			M
SIZ-674		Mother			F
SIZ-731	ASD, PE	Patient	Unknown	+	F
SIZ-732		Father			M
SIZ-733		Mother			F
SIZ-894	ASD, PE, cranial meningocele	Patient	Unknown	+	M
SIZ-895		Father			M
SIZ-896		Mother			F
SIZ-897	ASD, PE	Patient	-	+	M
SIZ-898		Father			M
SIZ-899		Mother			F
SIZ-978	ID, SGE	Patient	Unknown	+	F
SIZ-979		Father			M
SIZ-980		Mother			F
SIZ-990	mild ID, SGE	Patient	Unknown	+	F
SIZ-991		Mother			F
SIZ-992		Father			M
SIZ-965	ASD, suspected SMEI (*SCN1A* mutation-positive)	Patient	Unknown	+	F
SIZ-1007	ASD (*SCN1A* mutation-nagative)	Patient	Unknown	-	M
SIZ-1005		Father			M
SIZ-1006		Mother			F
SIZ-1008	ASD, BEECT or FLE	Patient	Unknown	+	M
SIZ-1009	ASD, BEECT or FLE	Patient	Unknown	+	M
SIZ-1015	ASD, ID, abnormal EEG	Patient	Unknown	-	M
SIZ-1003		Father			M
SIZ-1002		Mother			F
SIZ-1033	Asperger	Patient	Unknown	-	M
SIZ-1034	ASD, IE	Patient	Unknown	+	F
SIZ-1036		Father of SIZ-1034			M

SIZ-1035		Mother			F
SIZ-1070	ASD, PE	Patient	Unknown	+	F
SIZ-1071		Father			M
SIZ-1072		Mother			F
OKA-03-32	NDD, TLE	Patient	Unknown	+	M
OKA-03-33	NDD, TLE	Patient	Unknown	+	F
OKA-03-43	NDD, TLE	Patient	Unknown	+	M
OKA-03-56	NDD, TLE	Patient	Unknown	+	F
SIZ-87	ASD, ID, GE	Patient	Unknown	+	F
SIZ-178	severe ID, suspected FLE	Patient	Unknown	+	F
SIZ-396	ID (IQ 58), OLE	Patient	Unknown	+	M
SIZ-530	ID, OLE	Patient	Unknown	+	F
SIZ-575	ID (IQ 54), FLE	Patient	Unknown	+	F
SIZ-603	ID, FLE	Patient	Unknown	+	F
SIZ-779	ASD, PE	Patient	Unknown	+	F
SIZ-782	ASD, PE	Patient	Unknown	+	M
SIZ-1068	ASD, ID, Epilepsy	Patient	Unknown	+	M
SIZ-1076	ASD, ID, Epilepsy	Patient	Unknown	+	M
SIZ-1085	ASD, ID, Epilepsy	Patient	Unknown	+	M
SIZ-1101	ASD, ID, Epilepsy	Patient	Unknown	+	M

Supplementary Table 1. 95 NDD patients, their parents and associated phenotypes for whole exome sequencing.

ASD: Autism Spectrum Disorder, PDD: Pervasive Developmental Disorder, PDDNOS: PDD-Not Otherwise Specified, NDD: Neurodevelopmental Disorder, ADHD: Attention Deficit Hyperactivity Disorder, ID: Intellectual Disability, PE: Partial Epilepsy, SGE: Symptomatic Generalized Epilepsy, IE: Intractable Epilepsy, SMEI: Severe Myoclonic Epilepsy in Infancy - *SCN1A* mutation-negative cases were selected except for SIZ-965. ICEGTC: Intractable Childhood Epilepsy with Generalized Tonic Clonic seizures, BEECT: Benign Epilepsy of children with Centro-Temporal electroencephalogram (EEG) foci, TLE: Temporal Lobe Epilepsy, mTLE: mesial TLE, FLE: Frontal Lobe Epilepsy, OLE; Occipital Lobe Epilepsy, FS: Febrile Seizure, SCZ: Schizophrenia, IQ: Intelligence Quotient, DQ: Development Quotient, Y: Year-old, +: present, –: absent, M: Male, F: Female. Bold horizontal lines difine families. Upper 69 families with parents were used to identify *de novo* mutations.

NDD		Trigonocephaly		Epilepsy	WES	Targeted seq. of *PJA1 / MSX2*	Total
+	558** (548 families)	+	68	+	0	1	1
				–	6	11	17
				unknown	45	5	50
		–	1	+	1	0	1
				–	0	0	0
				unknown	0	0	0
		unkown	489	+	39	77	116
				–	4	2	6
				unknown	0	367	367
				**Total**	95*	463	558

Supplementary Table 2. Number of NDD patients per associated phenotypes and analysis.

*95 patients belong to 85 families. Parents genomic DNA was available for 79 patients from 69 families.

**The total of 558 patients includes 405 males and 153 females.

Number in parenthesis indicates the number of families recruited.

All *de novo*, hemizygous, homozygous and compound heterozygous mutations identified by WES have been confirmed by Sanger-sequencing in all samples.

All non-synonymous mutations in *PJA1* and *MSX2* identified by WES have been validated by Sanger-sequencing.

NDD: Neurodevelopmental Disorder, WES: Whole-Exome Sequencing, seq.: sequencing, +: present, –: absent.

Sample ID	Diagnosis	Note	Trigonocephaly	Epilepsy	Gender
OKI-053-1	Delay in speech acquisition, Motor developmental delay, ASD, Trigonocephaly	Patient	+	+	M
OKI-053-2		Father			M
OKI-053-3		Mother			F
OKI-054-1	Delay in speech acquisition, Motor developmental delay, Trigonocephaly	Patient	+	-	M
OKI-054-2		Father			M
OKI-054-3		Mother			F
OKI-056-1	Delay in speech acquisition, Motor developmental delay, ASD, Trigonocephaly	Patient	+	-	M
OKI-056-2		Father			M
OKI-056-3		Mother			F
OKI-057-1	Delay in speech acquisition, Motor developmental delay, Hyperkinesia, ASD, Trigonocephaly	Patient	+	-	M
OKI-057-2		Father			M
OKI-057-3		Mother			F
OKI-058-1	Delay in speech acquisition, Hyperkinesia, ASD, Regression, Trigonocephaly	Patient	+	-	M
OKI-058-2		Father			M
OKI-058-3		Mother			F
OKI-059-1	Delay in speech acquisition, Motor developmental delay, Hyperkinesia, Regression, Trigonocephaly	Patient	+	-	M
OKI-059-2		Father			M
OKI-059-3		Mother			F
OKI-060-1	Delay in speech acquisition, Motor developmental delay, Hyperkinesia, ASD, Trigonocephaly	Patient	+	-	M
OKI-060-2		Father			M
OKI-060-3		Mother			F
OKI-061-1	Delay in speech acquisition, Hyperkinesia, ASD, Trigonocephaly	Patient	+	-	M
OKI-061-2		Father			M
OKI-061-3		Mother			F
OKI-062-1	Delay in speech acquisition, Trigonocephaly	Patient	+	-	M
OKI-062-2		Father			M
OKI-062-3		Mother			F
OKI-064-1	Delay in speech acquisition, ASD, Trigonocephaly	Patient	+	-	M
OKI-064-2		Father			M
OKI-064-3		Mother			F
OKI-065-1	Delay in speech acquisition, Hyperkinesia, ASD, Trigonocephaly	Patient	+	-	M
OKI-065-2		Father			M
OKI-065-3		Mother			F
OKI-066-1	Delay in speech acquisition, Motor developmental delay, Hyperkinesia, hydrocephaly, Trigonocephaly	Patient	+	-	M
OKI-066-2		Father			M
OKI-066-3		Mother			F
OKI-033-1	Delay in speech acquisition, Hyperkinesia, ASD, Trigonocephaly	Patient	+	Unknown	M
OKI-033-3		Mother			F
OKI-049-1	Delay in speech acquisition, Hyperkinesia, Trigonocephaly	Patient	+	Unknown	M
OKI-049-3		Mother			F

OKI-051-1	Delay in speech acquisition, Hyperkinesia, Trigonocephaly	Patient	+	Unknown	M
OKI-051-2		Father			M
OKI-055-1	Hyperkinesia, Regression, Trigonocephaly	Patient	+	Unknown	M
OKI-055-2		Father			M
OKI-055-3		Mother			F
OKI-063-1	Motor developmental delay, Hyperkinesia, Trigonocephaly	Patient	+	Unknown	M
OKI-063-3		Father			F
OKI-063-4		Mother			M
OKA-42	NDD, Landau-Kleffner syndrome	Patient	Unknown	+	M
OKA-164	NDD, PE	Patient	Unknown	+	F
OKA-01-45	NDD, PE	Patient	Unknown	+	M
OKA-02-11	NDD, IGE	Patient	Unknown	+	F
OKA-02-12	NDD, PE	Patient	Unknown	+	M
OKA-02-13	NDD, IGE	Patient	Unknown	+	M
OKA-02-18	NDD, FLE	Patient	Unknown	+	M
OKA-02-25	NDD, PE	Patient	Unknown	+	M
OKA-03-62	NDD, Lennox Gastaut syndrome	Patient	Unknown	+	F
OKA-03-83	NDD, IGE	Patient	Unknown	+	M
SIZ-3	ID (IQ52), mTLE	Patient	Unknown	+	M
SIZ-4	ID (IQ56), mTLE	Patient	Unknown	+	F
SIZ-11	ID (IQ36), mTLE	Patient	Unknown	+	F
SIZ-92	ID (IQ25), FLE	Patient	Unknown	+	F
SIZ-107	SMEB (*SCN1A* mutation-negative) (DQ: 25)	Patient	Unknown	+	F
SIZ-109	SMEI (*SCN1A* mutation-negative) (DQ: 44)	Patient	Unknown	+	M
SIZ-116	severe ID, GE	Patient	Unknown	+	F
SIZ-136	ID (IQ 45), Epilepsy with myoclonic absence	Patient	Unknown	+	F
SIZ-158	moderate ID, ICEGTC	Patient	Unknown	+	M
SIZ-159	suspected SMEI (*SCN1A* mutation-negative) (DQ: 67)	Patient	Unknown	+	F
SIZ-171	ICEGTC (Walking instability, delay since 2y)	Patient	Unknown	+	F
SIZ-213	ID (IQ: 54), TLE	Patient	Unknown	+	M
SIZ-221	severe ID, ICEGTC	Patient	Unknown	+	M
SIZ-233	ID, Hyperkinesia, PE	Patient	Unknown	+	M
SIZ-236	developmental delay (IQ: 60), PE	Patient	Unknown	+	M
SIZ-242	developmental delay, PE	Patient	Unknown	+	M
SIZ-257	PE (DQ: 8)	Patient	Unknown	+	M
SIZ-274	ID, SGE	Patient	Unknown	+	M
SIZ-278	Landau-Kleffner syndrome (IQ33)	Patient	Unknown	+	M
SIZ-296	ID, Doose syndrome	Patient	Unknown	+	M
SIZ-361	severe ID, SGE	Patient	Unknown	+	F
SIZ-372	ID, Hyperkinesia, SGE	Patient	Unknown	+	M
SIZ-379	SGE (IQ 40)	Patient	Unknown	+	F
SIZ-386	PE (IQ: 47)	Patient	Unknown	+	M
SIZ-387	ID (IQ: 52), SMEB (*SCN1A* mutation-negative)	Patient	Unknown	+	F
SIZ-389	PE (IQ 61)	Patient	Unknown	+	F
SIZ-395	ID, TLE	Patient	Unknown	+	M
SIZ-450	PE (IQ20)	Patient	Unknown	+	F
SIZ-480	mild developmental delay, Doose syndrome	Patient	Unknown	+	M
SIZ-490	Hyperthermia induced partial epilepsy (IQ 65)	Patient	Unknown	+	F
SIZ-509	ID, Coppora	Patient	Unknown	+	F

SIZ-514	suspected SMEI (*SCN1A* mutation-negative) (DQ: 40)	Patient	Unknown	+	F
SIZ-541	ID, PE	Patient	Unknown	+	F
SIZ-544	Doose syndrome (IQ: 61)	Patient	Unknown	+	M
SIZ-645	ADHD (DQ: 45), PE	Patient	Unknown	+	M
SIZ-663	PE (DQ 53)	Patient	Unknown	+	M
SIZ-669	SGE (IQ 50)	Patient	Unknown	+	F
SIZ-682	ASD, PE	Patient	Unknown	+	M
SIZ-683	Chronic relapsing encephalitis (DQ 60)	Patient	Unknown	-	F
SIZ-696	ASD, epilepsy, sleep disorders	Patient	Unknown	+	M
SIZ-704	TLE (IQ 47)	Patient	Unknown	+	M
SIZ-706	ID, suspected GEFS	Patient	Unknown	+	F
SIZ-707	ID, SGE	Patient	Unknown	+	M
SIZ-722	ICEGTC (DQ 26)	Patient	Unknown	+	F
SIZ-742	developmental delay, PE	Patient	Unknown	+	F
SIZ-743	ASD, SGE	Patient	Unknown	+	F
SIZ-750	SGE (IQ 56)	Patient	Unknown	+	M
SIZ-754	Sturge-Weber syndrome (DQ 62, PE,	Patient	Unknown	+	M
SIZ-755	SGE (IQ 54)	Patient	Unknown	+	M
SIZ-789	ASD, PE	Patient	Unknown	+	M
SIZ-808	ID, SGE	Patient	Unknown	+	F
SIZ-831	ID (IQ28), PE	Patient	Unknown	+	F
SIZ-846	ASD, hyperthermia-induced partial epilepsy	Patient	Unknown	+	F
SIZ-928	severe ID, suspected SMEI (*SCN1A* mutation-negative)	Patient	Unknown	+	M
SIZ-929	MAE (IQ 65)	Patient	Unknown	+	M
SIZ-953	developmental delay, PE	Patient	Unknown	+	F
SIZ-966	ASD	Patient	Unknown	-	F
SIZ-1065	ASD, Epilepsy	Patient	Unknown	+	M
SIZ-1090	ASD, Dravet	Patient	Unknown	+	M
SIZ-1100	ASD, Epilepsy	Patient	Unknown	+	M
NAG-ASP001	NDD	Patient	Unknown	Unknown	M
NAG-ASP002	PDDNOS	Patient	Unknown	Unknown	F
NAG-ASP003	NDD	Patient	Unknown	Unknown	F
NAG-ASP004	NDD	Patient	Unknown	Unknown	F
NAG-ASP005	NDD	Patient	Unknown	Unknown	M
NAG-ASP006	NDD	Patient	Unknown	Unknown	F
NAG-ASP007	ASD	Patient	Unknown	Unknown	F
NAG-ASP008	NDD	Patient	Unknown	Unknown	M
NAG-ASP009	NDD	Patient	Unknown	Unknown	F
NAG-ASP010	NDD	Patient	Unknown	Unknown	M
NAG-ASP011	NDD	Patient	Unknown	Unknown	F
NAG-ASP012	NDD	Patient	Unknown	Unknown	M
NAG-ASP013	NDD	Patient	Unknown	Unknown	M
NAG-ASP015	NDD	Patient	Unknown	Unknown	F
NAG-ASP003	NDD	Patient	Unknown	Unknown	M
NAG-ASP018	NDD	Patient	Unknown	Unknown	F
NAG-ASP020	NDD	Patient	Unknown	Unknown	M
NAG-ASP021	NDD	Patient	Unknown	Unknown	F
NAG-ASP024	NDD	Patient	Unknown	Unknown	M
NAG-ASP025	NDD	Patient	Unknown	Unknown	M
NAG-ASP026	NDD	Patient	Unknown	Unknown	M
NAG-ASP028	NDD	Patient	Unknown	Unknown	M
NAG-ASP029	NDD	Patient	Unknown	Unknown	M
NAG-ASP030	NDD	Patient	Unknown	Unknown	M
NAG-ASP031	NDD	Patient	Unknown	Unknown	F
NAG-ASP032	NDD	Patient	Unknown	Unknown	F
NAG-ASP033	NDD	Patient	Unknown	Unknown	F
NAG-ASP035	NDD	Patient	Unknown	Unknown	M

NAG-ASP037	NDD	Patient	Unknown	Unknown	M
NAG-ASP038	NDD	Patient	Unknown	Unknown	M
NAG-ASP039	NDD	Patient	Unknown	Unknown	M
NAG-ASP040	NDD	Patient	Unknown	Unknown	M
NAG-ASP043	NDD	Patient	Unknown	Unknown	M
NAG-ASP044	NDD	Patient	Unknown	Unknown	M
NAG-ASP048	NDD	Patient	Unknown	Unknown	F
NAG-ASP049	NDD	Patient	Unknown	Unknown	F
NAG-ASP051	NDD	Patient	Unknown	Unknown	M
NAG-ASP052	NDD	Patient	Unknown	Unknown	M
NAG-ASP053	NDD	Patient	Unknown	Unknown	M
NAG-ASP054	NDD	Patient	Unknown	Unknown	M
NAG-ASP056	NDD	Patient	Unknown	Unknown	M
NAG-ASP057	NDD	Patient	Unknown	Unknown	F
NAG-ASP058	NDD	Patient	Unknown	Unknown	F
NAG-ASP059	NDD	Patient	Unknown	Unknown	M
NAG-ASP060	NDD	Patient	Unknown	Unknown	F
NAG-ASP061	NDD	Patient	Unknown	Unknown	M
NAG-ASP062	NDD	Patient	Unknown	Unknown	F
NAG-ASP063	NDD	Patient	Unknown	Unknown	F
NAG-ASP064	NDD	Patient	Unknown	Unknown	F
NAG-ASP065	PDDNOS	Patient	Unknown	Unknown	M
NAG-ASP066	PDDNOS	Patient	Unknown	Unknown	F
NAG-ASP068	NDD	Patient	Unknown	Unknown	F
NAG-ASP069	NDD	Patient	Unknown	Unknown	M
NAG-ASP071	NDD	Patient	Unknown	Unknown	M
NAG-COLPDD00001	NDD	Patient	Unknown	Unknown	M
NAG-COLPDD00002	NDD	Patient	Unknown	Unknown	M
NAG-NP001	NDD	Patient	Unknown	Unknown	M
NAG-NP002	NDD	Patient	Unknown	Unknown	M
NAG-NP004	NDD	Patient	Unknown	Unknown	M
NAG-NP007	NDD	Patient	Unknown	Unknown	M
NAG-NP008	NDD	Patient	Unknown	Unknown	M
NAG-NP009	NDD	Patient	Unknown	Unknown	M
NAG-NP011	NDD	Patient	Unknown	Unknown	M
NAG-NP012	NDD	Patient	Unknown	Unknown	M
NAG-NP013	NDD	Patient	Unknown	Unknown	M
NAG-NP014	NDD	Patient	Unknown	Unknown	M
NAG-NP016	NDD	Patient	Unknown	Unknown	F
NAG-NP021	NDD	Patient	Unknown	Unknown	M
NAG-NP022	NDD	Patient	Unknown	Unknown	F
NAG-NP023	NDD	Patient	Unknown	Unknown	M
NAG-NP024	NDD	Patient	Unknown	Unknown	M
NAG-NP027	NDD	Patient	Unknown	Unknown	M
NAG-NP028	NDD	Patient	Unknown	Unknown	F
NAG-NP030	NDD	Patient	Unknown	Unknown	M
NAG-NP032	NDD	Patient	Unknown	Unknown	M
NAG-NP033	NDD	Patient	Unknown	Unknown	M
NAG-NP034	NDD	Patient	Unknown	Unknown	M
NAG-NP035	NDD	Patient	Unknown	Unknown	M
NAG-NP038	NDD	Patient	Unknown	Unknown	M
NAG-NP039	PDDNOS	Patient	Unknown	Unknown	M
NAG-NP040	NDD	Patient	Unknown	Unknown	M
NAG-NP041	NDD	Patient	Unknown	Unknown	F
NAG-NP042	NDD	Patient	Unknown	Unknown	F
NAG-NP044	NDD	Patient	Unknown	Unknown	M
NAG-NP045	NDD	Patient	Unknown	Unknown	F
NAG-NP047	NDD	Patient	Unknown	Unknown	M
NAG-NP050	PDD, ID	Patient	Unknown	Unknown	M
NAG-NP051	NDD	Patient	Unknown	Unknown	M

NAG-NP053	NDD	Patient	Unknown	Unknown	F
NAG-NP054	NDD	Patient	Unknown	Unknown	M
NAG-NP056	NDD	Patient	Unknown	Unknown	M
NAG-NP057	NDD	Patient	Unknown	Unknown	M
NAG-NP058	NDD	Patient	Unknown	Unknown	M
NAG-NP060	NDD	Patient	Unknown	Unknown	M
NAG-NP062	NDD	Patient	Unknown	Unknown	F
NAG-NP063	NDD	Patient	Unknown	Unknown	M
NAG-NP064	NDD	Patient	Unknown	Unknown	M
NAG-NP065	NDD	Patient	Unknown	Unknown	F
NAG-NP067	NDD	Patient	Unknown	Unknown	M
NAG-NP068	NDD	Patient	Unknown	Unknown	M
NAG-NP070	NDD	Patient	Unknown	Unknown	M
NAG-NP071	NDD	Patient	Unknown	Unknown	M
NAG-NP072	NDD	Patient	Unknown	Unknown	M
NAG-NP079	NDD	Patient	Unknown	Unknown	M
NAG-NP080	NDD	Patient	Unknown	Unknown	M
NAG-NP081	NDD	Patient	Unknown	Unknown	F
NAG-NP083	NDD	Patient	Unknown	Unknown	M
NAG-NP085	Asperger	Patient	Unknown	Unknown	M
NAG-NP086	NDD	Patient	Unknown	Unknown	F
NAG-NP087	NDD	Patient	Unknown	Unknown	M
NAG-NP089	NDD	Patient	Unknown	Unknown	F
NAG-NP094	NDD	Patient	Unknown	Unknown	M
NAG-NP095	NDD	Patient	Unknown	Unknown	M
NAG-NP096	NDD	Patient	Unknown	Unknown	M
NAG-NP098	NDD	Patient	Unknown	Unknown	F
NAG-NP099	NDD	Patient	Unknown	Unknown	M
NAG-NP100	NDD	Patient	Unknown	Unknown	M
NAG-NP101	NDD	Patient	Unknown	Unknown	M
NAG-NP102	Asperger	Patient	Unknown	Unknown	F
NAG-NP104	NDD	Patient	Unknown	Unknown	M
NAG-NP105	NDD	Patient	Unknown	Unknown	F
NAG-NP106	NDD	Patient	Unknown	Unknown	F
NAG-NP107	NDD	Patient	Unknown	Unknown	M
NAG-NP108	NDD	Patient	Unknown	Unknown	F
NAG-NP109	NDD	Patient	Unknown	Unknown	M
NAG-NP110	NDD	Patient	Unknown	Unknown	M
NAG-NP113	NDD	Patient	Unknown	Unknown	F
NAG-NP114	NDD	Patient	Unknown	Unknown	M
NAG-NP115	NDD	Patient	Unknown	Unknown	M
NAG-NP116	NDD	Patient	Unknown	Unknown	M
NAG-NP117	NDD	Patient	Unknown	Unknown	M
NAG-NP118	NDD	Patient	Unknown	Unknown	F
NAG-NP119	NDD	Patient	Unknown	Unknown	M
NAG-NP120	NDD	Patient	Unknown	Unknown	F
NAG-NP121	NDD	Patient	Unknown	Unknown	M
NAG-NP122	NDD	Patient	Unknown	Unknown	M
NAG-NP123	NDD	Patient	Unknown	Unknown	M
NAG-NP124	NDD	Patient	Unknown	Unknown	M
NAG-NP126	NDD	Patient	Unknown	Unknown	M
NAG-NP127	NDD	Patient	Unknown	Unknown	M
NAG-NP128	NDD	Patient	Unknown	Unknown	M
NAG-NP131	PDDNOS	Patient	Unknown	Unknown	M
NAG-NP133	NDD	Patient	Unknown	Unknown	M
NAG-NP134	NDD	Patient	Unknown	Unknown	F
NAG-NP135	NDD	Patient	Unknown	Unknown	M
NAG-NP137	NDD	Patient	Unknown	Unknown	F
NAG-NP138	NDD	Patient	Unknown	Unknown	M
NAG-NP140	NDD	Patient	Unknown	Unknown	M

NAG-NP141	NDD	Patient	Unknown	Unknown	M
NAG-NP142	NDD	Patient	Unknown	Unknown	M
NAG-NP143	NDD	Patient	Unknown	Unknown	F
NAG-NP144	NDD	Patient	Unknown	Unknown	M
NAG-NP146	NDD	Patient	Unknown	Unknown	M
NAG-NP147	NDD	Patient	Unknown	Unknown	M
NAG-NP148	NDD	Patient	Unknown	Unknown	F
NAG-NP149	PDDNOS, ADHD	Patient	Unknown	Unknown	F
NAG-NP151	NDD	Patient	Unknown	Unknown	F
NAG-NP154	NDD	Patient	Unknown	Unknown	M
NAG-NP155	NDD	Patient	Unknown	Unknown	F
NAG-NP157	NDD	Patient	Unknown	Unknown	M
NAG-NP161	NDD	Patient	Unknown	Unknown	M
NAG-NP162	NDD	Patient	Unknown	Unknown	M
NAG-NP166	NDD	Patient	Unknown	Unknown	M
NAG-NP167	NDD	Patient	Unknown	Unknown	M
NAG-NP171	NDD	Patient	Unknown	Unknown	M
NAG-NP173	NDD	Patient	Unknown	Unknown	M
NAG-NP178	NDD	Patient	Unknown	Unknown	M
NAG-NP179	NDD	Patient	Unknown	Unknown	M
NAG-NP180	NDD	Patient	Unknown	Unknown	M
NAG-NP184	NDD	Patient	Unknown	Unknown	M
NAG-NP186	Asperger, SCZ	Patient	Unknown	Unknown	M
NAG-NP187	NDD	Patient	Unknown	Unknown	M
NAG-YP001	NDD	Patient	Unknown	Unknown	M
NAG-YP002	NDD	Patient	Unknown	Unknown	M
NAG-YP003	NDD	Patient	Unknown	Unknown	M
NAG-YP004	NDD	Patient	Unknown	Unknown	M
NAG-YP005	NDD	Patient	Unknown	Unknown	F
NAG-YP006	NDD	Patient	Unknown	Unknown	M
NAG-YP008	NDD	Patient	Unknown	Unknown	M
NAG-YP014	NDD	Patient	Unknown	Unknown	M
NAG-YP016	NDD	Patient	Unknown	Unknown	M
NAG-YP019	NDD	Patient	Unknown	Unknown	M
NAG-YP020	NDD	Patient	Unknown	Unknown	M
NAG-YP021	NDD	Patient	Unknown	Unknown	M
NAG-YP022	NDD	Patient	Unknown	Unknown	M
NAG-YP028	NDD	Patient	Unknown	Unknown	M
NAG-YP029	NDD	Patient	Unknown	Unknown	F
AP001_1	ASD	Patient	Unknown	Unknown	F
AP002_1	ASD	Patient	Unknown	Unknown	M
AP003_1	NDD	Patient	Unknown	Unknown	M
AP004_1	ASD	Patient	Unknown	Unknown	F
AP005_1	ASD	Patient	Unknown	Unknown	M
AP006_1	ASD	Patient	Unknown	Unknown	F
AP007_1	PDDNOS	Patient	Unknown	Unknown	M
AP008_1	ASD	Patient	Unknown	Unknown	F
AP011_1	ASD	Patient	Unknown	Unknown	M
AP013_1	ASD	Patient	Unknown	Unknown	F
AP016_1	Asperger	Patient	Unknown	Unknown	F
AP017_1	ASD	Patient	Unknown	Unknown	M
AP018_1	ASD	Patient	Unknown	Unknown	M
AP019_1	PDDNOS	Patient	Unknown	Unknown	M
AP020_1	PDDNOS	Patient	Unknown	Unknown	M
AP023_1	ASD	Patient	Unknown	Unknown	M
AP025_1	PDDNOS	Patient	Unknown	Unknown	M
AP026_1	PDDNOS	Patient	Unknown	Unknown	M
AP027_1	PDDNOS	Patient	Unknown	Unknown	M
AP028_1	ASD	Patient	Unknown	Unknown	M
AP029_1	ASD	Patient	Unknown	Unknown	M

AP030_1	PDDNOS	Patient	Unknown	Unknown	M
AP031_1	ASD	Patient	Unknown	Unknown	M
AP032_1	ASD	Patient	Unknown	Unknown	M
AP036_1	ASD	Patient	Unknown	Unknown	M
AP037_1	HFASD	Patient	Unknown	Unknown	M
AP039_1	PDDNOS	Patient	Unknown	Unknown	M
AP040_1	ASD	Patient	Unknown	Unknown	M
AP041_1	ASD	Patient	Unknown	Unknown	M
AP042_1	Asperger	Patient	Unknown	+	M
AP043_1	ASD	Patient	Unknown	Unknown	M
AP044_1	ASD	Patient	Unknown	Unknown	M
AP045_1	ASD	Patient	Unknown	Unknown	M
AP046_1	Asperger	Patient	Unknown	Unknown	M
AP047_1	HFASD	Patient	Unknown	Unknown	M
AP049_1	ASD	Patient	Unknown	Unknown	M
AP051_1	ASD	Patient	Unknown	Unknown	M
AP052_1	ASD	Patient	Unknown	Unknown	M
AP055_1	ASD	Patient	Unknown	Unknown	M
AP057_1	ASD	Patient	Unknown	Unknown	M
AP063_1	ASD	Patient	Unknown	Unknown	M
AP066_1	PDDNOS	Patient	Unknown	Unknown	M
AP069_1	ASD	Patient	Unknown	Unknown	M
AP070_1	ASD	Patient	Unknown	Unknown	M
AP071_1	HFPDD	Patient	Unknown	Unknown	M
AP074_1	ASD	Patient	Unknown	Unknown	M
AP075_1	ASD	Patient	Unknown	Unknown	M
AP078_1	PDDNOS	Patient	Unknown	Unknown	M
AP079_1	ASD	Patient	Unknown	Unknown	M
AP080_1	ASD	Patient	Unknown	Unknown	M
AP081_1	PDDNOS	Patient	Unknown	Unknown	M
AP082_1	ASD	Patient	Unknown	Unknown	M
AP083_1	ASD	Patient	Unknown	Unknown	M
AP084_1	ASD	Patient	Unknown	+	M
AP085_1	ASD	Patient	Unknown	Unknown	F
AP087_1	ASD	Patient	Unknown	Unknown	M
AP088_1	PDD	Patient	Unknown	Unknown	M
AP090_1	ASD	Patient	Unknown	Unknown	M
AP091_1	PDDNOS	Patient	Unknown	Unknown	F
AP092_1	PDDNOS	Patient	Unknown	Unknown	F
AP095_1	PDDNOS	Patient	Unknown	Unknown	M
AP097_1	ASD	Patient	Unknown	Unknown	M
AP098_1	ASD	Patient	Unknown	Unknown	M
AP099_1	ASD	Patient	Unknown	Unknown	M
AP102_1	ASD	Patient	Unknown	Unknown	M
AP107_1	ASD	Patient	Unknown	Unknown	M
AP108_1	PDDNOS	Patient	Unknown	Unknown	M
AP110_1	ASD	Patient	Unknown	Unknown	M
AP112_1	ASD	Patient	Unknown	Unknown	M
AP118_1	ASD	Patient	Unknown	Unknown	F
AP119_1	ASD	Patient	Unknown	Unknown	M
AP121_1	ASD	Patient	Unknown	Unknown	M
AP123_1	ASD	Patient	Unknown	Unknown	M
AP124_1	PDDNOS	Patient	Unknown	Unknown	M
AP126_1	ASD	Patient	Unknown	Unknown	F
AP127_1	ASD	Patient	Unknown	Unknown	M
AP128_1	PDDNOS	Patient	Unknown	Unknown	F
AP136_1	PDDNOS	Patient	Unknown	Unknown	M
AP138_1	PDDNOS	Patient	Unknown	Unknown	F
AP139_1	ASD	Patient	Unknown	Unknown	M
AP141_1	ASD	Patient	Unknown	Unknown	M

AP142_1	ASD	Patient	Unknown	Unknown	M
AP148_1	ASD	Patient	Unknown	Unknown	M
AP149_1	ASD	Patient	Unknown	Unknown	M
AP150_1	PDDNOS	Patient	Unknown	Unknown	M
AP158_1	ASD	Patient	Unknown	Unknown	M
AP159_1	PDDNOS	Patient	Unknown	Unknown	M
AP160_1	Asperger	Patient	Unknown	Unknown	M
AP161_1	ASD	Patient	Unknown	Unknown	M
AP162_1	PDDNOS	Patient	Unknown	Unknown	M
AP163_1	ASD	Patient	Unknown	Unknown	M
AP164_1	ASD	Patient	Unknown	Unknown	M
AP165_1	ASD	Patient	Unknown	Unknown	F
AP166_1	PDDNOS	Patient	Unknown	Unknown	M
AP168_1	ASD	Patient	Unknown	Unknown	M
AP169_1	ASD	Patient	Unknown	Unknown	M
AP171_1	ASD	Patient	Unknown	Unknown	F
AP174_1	ASD	Patient	Unknown	Unknown	M
AP175_1	PDDNOS	Patient	Unknown	Unknown	M
AP177_1	ASD	Patient	Unknown	Unknown	M
AP184_1	ASD	Patient	Unknown	Unknown	F
AP188_1	ASD	Patient	Unknown	Unknown	M
AP196_1	NDD	Patient	Unknown	Unknown	M
AP197_1	PDDNOS	Patient	Unknown	Unknown	F
AP202_1	NDD	Patient	Unknown	Unknown	M
AP204_1	ASD	Patient	Unknown	Unknown	M
AP205_1	Asperger	Patient	Unknown	Unknown	F
AP210_1	PDDNOS	Patient	Unknown	Unknown	M
AP211_1	NDD	Patient	Unknown	Unknown	F
AP212_1	Asperger	Patient	Unknown	Unknown	M
AP213_1	ASD	Patient	Unknown	Unknown	M
AP215_1	PDDNOS	Patient	Unknown	Unknown	M
AP230_1	NDD	Patient	Unknown	Unknown	M
AP233_1	HFASD	Patient	Unknown	Unknown	M
AP234_1	ASD	Patient	Unknown	Unknown	M
AP238_1	ASD	Patient	Unknown	Unknown	M
AP239_1	PDD	Patient	Unknown	Unknown	M
AP240_1	PDD	Patient	Unknown	Unknown	M
AP245_1	NDD	Patient	Unknown	Unknown	M
AP250_1	PDDNOS	Patient	Unknown	Unknown	M
AP255_1	NDD	Patient	Unknown	Unknown	M
AP256_1	NDD	Patient	Unknown	Unknown	M
HM148_3	Asperger	Patient	Unknown	Unknown	M
HM149_3	Asperger	Patient	Unknown	Unknown	M
RUM003	ASD	Patient	Unknown	Unknown	M
RUM007	ASD, suspected Epilepsy	Patient	Unknown	Unknown	M
RUM010	ASD	Patient	Unknown	Unknown	M
RUM013	PDDNOS	Patient	Unknown	Unknown	M
RUM016	ASD	Patient	Unknown	Unknown	M
RUM022	ASD, FS	Patient	Unknown	Unknown	M
RUM025	ASD	Patient	Unknown	Unknown	F
RUM031	ASD	Patient	Unknown	Unknown	F
RUM037	ASD	Patient	Unknown	Unknown	F
RUM040	ASD	Patient	Unknown	Unknown	F
RUM043	ASD	Patient	Unknown	Unknown	F
RUM047	ASD	Patient	Unknown	Unknown	M
RUM056	ASD, West Syndrome	Patient	Unknown	+	M
RUM059	ASD	Patient	Unknown	Unknown	M
RUM062	ASD	Patient	Unknown	Unknown	M
RUM065	ASD	Patient	Unknown	Unknown	M
RUM069	ASD	Patient	Unknown	Unknown	M

RUM072	PDDNOS	Patient	Unknown	Unknown	M
RUM075	ASD	Patient	Unknown	Unknown	M
RUM087	ASD	Patient	Unknown	Unknown	M
RUM093	ASD	Patient	Unknown	Unknown	M
RUM096	ASD	Patient	Unknown	Unknown	M
RUM099	ASD	Patient	Unknown	Unknown	M
RUM106	ASD	Patient	Unknown	Unknown	M
RUM109	ASD, suspected West Syndrome	Patient	Unknown	+	M
RUM112	ASD, FS	Patient	Unknown	Unknown	M
RUM119	ASD	Patient	Unknown	Unknown	M
RUM122	PDDNOS, Afebrile convulsion	Patient	Unknown	Unknown	M
RUM125	ASD	Patient	Unknown	Unknown	M
RUM128	ASD	Patient	Unknown	Unknown	F
RUM131	ASD	Patient	Unknown	Unknown	M
RUM134	ASD, FS	Patient	Unknown	Unknown	F
RUM137	ASD	Patient	Unknown	Unknown	M
RUM142	ASD, FS	Patient	Unknown	Unknown	M
RUM145	ASD, FS	Patient	Unknown	Unknown	F
RUM152	ASD	Patient	Unknown	Unknown	M
RUM155	ASD, Afebrile convulsion	Patient	Unknown	Unknown	M
RUM158	ASD	Patient	Unknown	Unknown	M
RUM161	ASD	Patient	Unknown	Unknown	M
RUM164	PDDNOS	Patient	Unknown	Unknown	F
RUM167	ASD	Patient	Unknown	Unknown	M
RUM170	ASD	Patient	Unknown	Unknown	M
RUM173	ASD	Patient	Unknown	Unknown	M
RUM177	ASD	Patient	Unknown	+	M
RUM180	ASD	Patient	Unknown	Unknown	M
RUM183	ASD	Patient	Unknown	Unknown	M
RUM186	ASD	Patient	Unknown	Unknown	M
RUM192	ASD	Patient	Unknown	Unknown	M
RUM198	ASD	Patient	Unknown	+	M
RUM201	PDDNOS, FS, Absence seizures	Patient	Unknown	+	F
RUM204	ASD	Patient	Unknown	Unknown	M
RUM207	ASD	Patient	Unknown	Unknown	F
RUM216	ASD	Patient	Unknown	Unknown	M
RUM219	ASD	Patient	Unknown	Unknown	F
RUM222	ASD	Patient	Unknown	+	M
RUM225	PDDNOS	Patient	Unknown	Unknown	F
RUM228	ASD	Patient	Unknown	Unknown	M
RUM231	ASD	Patient	Unknown	Unknown	F
RUM234	ASD	Patient	Unknown	Unknown	M
RUM237	ASD	Patient	Unknown	Unknown	M
RUM240	PDDNOS	Patient	Unknown	Unknown	M
RUM243	ASD	Patient	Unknown	+	M
RUM246	PDDNOS	Patient	Unknown	Unknown	M
RUM249	ASD	Patient	Unknown	Unknown	M
RUM252	ASD	Patient	Unknown	Unknown	M

Supplementary Table 3. 463 NDD patients, their parents and associated phenotypes for targeted sequencing.

ASD: Autism Spectrum Disorder, HF ASD: High Function ASD, PDD: Pervasive Developmental Disorder, HFPDD: High Function PDD, PDDNOS: PDD-Not Otherwise Specified, NDD: Neurodevelopmental Disorder, ADHD: Attention Deficit Hyperactivity Disorder, ID: Intellectual Disability, PE: Partial Epilepsy, SGE: Symptomatic Generalized Epilepsy, IE: Intractable Epilepsy, SMEI: Severe Myoclonic Epilepsy in Infancy - *SCN1A* mutation-negative cases were selected except for SIZ-965. ICEGTC: Intractable Childhood Epilepsy with Generalized Tonic Clonic seizures, BEECT: Benign Epilepsy of children with Centro-Temporal electroencephalogram (EEG) foci, TLE: Temporal Lobe Epilepsy, mTLE: mesial TLE, FLE: Frontal Lobe Epilepsy, OLE; Occipital Lobe Epilepsy, FS: Febrile Seizure, SCZ: Schizophrenia, IQ: Intelligence Quotient, DQ: Development Quotient, Y: Year-old, +: present, –: absent, M: Male, F: Female. Bold horizontal lines difine families.

Patient ID	Gender	Trigonocephaly	Epilepsy	Gene	Chr.	Position (hg19)	Nucleotide change	RefSeq gene ID	Amino acid change	Mutant allele count in In-house controls	Mutation taster[1]	PolyPhen2[2]	SIFT[3]
SIZ-894	M	Unknown	+	***MSX2***	5	174156298_174156299	**c.516_517insG**	NM_002449	**p.Ala173fs**	0 / 1150	+	N/A	N/A
SIZ-672	F	Unknown	+	***SCN1A****	2	166,903,486	c.1189T>G	NM_006920	p.Thr391Pro	0 / 1150	+	+	+
SIZ-965	F	Unknown	+			166,852,550_166,852,551	**c.4550_4551delAA**	NM_001165963	**p.Lys1517fs**	0 / 1150	+	N/A	N/A
				HK1	10	71,139,786	c.1200C>A	NM_000188	p.Asn400Lys	0 / 1150	+	+	-
SIZ-1034	F	Unknown	+	***IQSEC2***	X	53,285,131_53,285,132	**c.849_850insG**	NM_001111125	**p.Gly283fs**	0 / 869	+	N/A	N/A
OKI-009-1	F	+	Unknown	***DDX3X***	X	41,206,110	**c.1616-2A>G**	NM_001193416	**3' splice site**	0 / 869	N/A	N/A	N/A
				ZSWIM8	10	75,551,888	c.1591C>T	NM_001242487	p.Pro531Ser	0 / 1150	-	-	-
OKI-011-1	F	+	-	***CYB5R4***	6	84,624,133	**c.413-2A>C**	NM_016230	**3' splice site**	0 / 1150	N/A	N/A	N/A
				RBM15B	3	51,429,016	c.186C>G	NM_013286	p.Ser62Arg	0 / 1150	+	-	+
				ZBTB7A	19	4,054,699	c.532G>A	NM_015898	p.Ala178Thr	0 / 1150	-	+	N/A
OKI-011-2	M	+	Unknown	***SEL1L2***	20	13,856,763	**c.1027-2A>C**	NM_001271539	**3' splice site**	0 / 1150	N/A	N/A	N/A
OKI-017-1	M	+	Unknown	***C14orf119***	14	23,567,024	**c.157C>T**	NM_017924	**p.Gln53X**	0 / 1150	+	N/A	N/A
				INO80D	2	206,869,651	c.2525A>G	NM_017759	p.His842Arg	0 / 1150	+	-	+
OKI-019-1	M	+	Unknown	***RAB11FIP2***	10	119,805,472_119,805,473	**c.202_203delCT**	NM_014904	**p.Leu68fs**	0 / 1150	+	N/A	N/A
				ZMYND8	20	45,867,698_45,867,702	**c.2249_2253delAGAGG**	NM_001281781	**p.Gln750fs**	0 / 1150	+	N/A	N/A
OKI-022-1	F	+	Unknown	***STXBP1***	9	130,428,580	**c.794+5G>C**	NM_001032221	**5' splice site**	0 / 1150	N/A	N/A	N/A
OKI-029-1	F	+	Unknown	***CACNA1E***	1	181,719,628	**c.3674+5A>G**	NM_001205294	**5' splice site**	0 / 1150	N/A	N/A	N/A
OKI-030-1	F	+	Unknown	***CYP1A1***	15	75,015,334_75,015,344	**c.95_105delAGGTCCCCAAA**	NM_000499	**p.Gln32fs**	0 / 1150	+	N/A	N/A
						75,015,345	c.94C>G	NM_000499	p.Gln32Glu	0 / 1150	-	-	-
OKI-032-1	M	+	Unknown	***FLII***	17	18,157,447	**c.625C>T**	NM_002018	**p.Gln209X**	0 / 1150	+	N/A	N/A
				EIF3A	10	120,828,988	c.920G>T	NM_003750	p.Arg307Ile	0 / 1150	+	+	+
						120,828,989	c.919A>G	NM_003750	p.Arg307Gly	0 / 1150	+	+	-
						120,829,004	c.904C>T	NM_003750	p.Leu302Phe	0 / 1150	+	+	+
						120,829,009	c.899A>C	NM_003750	p.Tyr300Ser	0 / 1150	+	+	+
				OBSCN	1	228,468,139	c.7923G>C	NM_052843	p.Glu2641Asp	0 / 1150	-	+	-
				CTNNA3	10	67,862,971	c.1921G>A	NM_013266	p.Glu641Lys	0 / 1150	+	-	-
OKI-035-1	F	+	Unknown	***WHSC1***	4	1,905,942	**c.598-1G>C**	NM_001042424	**3' splice site**	0 / 1150	N/A	N/A	N/A
OKI-042-1	M	+	-	***PHF21A*****	11	45,957,234	**c.1738C>T**	NM_001101802	**p.Arg580X**	0 / 1150	+	N/A	N/A
				SCRIB	8	144,887,467	c.2485C>T	NM_015356	p.Arg829Trp	0 / 1150	+	+	+
OKI-044-1	M	+	Unknown	***ZNF143***	11	9,499,932	**c.374-5C>T**	NM_003442	**3' splice site**	0 / 1150	N/A	N/A	N/A
OKI-047-1	M	+	Unknown	***ARID1B***	6	157,406,035	**c.2277delC**	NM_020732	**p.Pro760fs**	0 / 1150	+	N/A	N/A
OKI-002-1	M	+	Unknown	*SH3RF3*	2	110,259,084	c.2485C>T	NM_001099289	p.Arg829Cys	0 / 1150	+	+	+
OKI-007-1	M	+	Unknown	*PTPRS*	19	5,221,096	c.3370G>A	NM_002850	p.Gly1124Ser	0 / 1150	-	-	-
OKI-008-1	F	+	Unknown	*SEMA4C*	2	97,526,862	c.2003C>T	NM_017789	p.Ala668Val	0 / 1150	+	-	-
				DGKH	13	42,783,470	c.2719C>G	NM_001204504	p.Arg907Gly	0 / 1150	+	+	+
OKI-016-1	F	+	Unknown	*CYP24A1*	20	52,779,317	c.929G>A	NM_000782	p.Arg310Gln	0 / 1150	-	-	-
OKI-021-1	M	+	Unknown	*KCTD1*	18	24,128,230	c.271G>A	NM_001142730	p.Glu91Lys	0 / 1150	-	-	+
OKI-024-1	M	+	Unknown	*TIMMDC1*	3	119,217,596	c.16C>G	NM_016589	p.Pro6Ala	0 / 1150	-	-	-
				FAT1	4	187,558,062	c.3649G>T	NM_005245	p.Val1217Leu	0 / 1150	+	+	+
OKI-025-1	M	+	Unknown	*STAG1*	3	136,059,425	c.3580G>A	NM_005862	p.Ala1194Thr	0 / 1150	+	+	+
OKI-026-1	M	+	Unknown	*KIF3C*	2	26,203,529	c.1258G>A	NM_002254	p.Gly420Arg	0 / 1150	+	+	-
				SLC12A4	16	67,997,460	c.118C>T	NM_001145961	p.His40Tyr	0 / 1150	+	-	-
OKI-027-1	M	+	Unknown	*WDFY4*	10	49,939,479	c.1454T>A	NM_020945	p.Met485Lys	0 / 1150	-	-	+
				ANKS1B	12	99,201,638	c.140T>C	NM_001204065	p.Ile207Thr	0 / 1150	-	-	-
OKI-031-1	F	+	Unknown	*TTC40*	10	134,660,510	c.6193G>A	NM_001200049	p.Ala2065Thr	0 / 1150	-	+	+
OKI-040-1	M	+	Unknown	*GNL3*	3	52,727,264	c.1106C>T	NM_014366	p.Ala369Val	0 / 1150	+	+	+
OKI-043-1	M	+	Unknown	*KIAA0196*	8	126,069,893	c.1780G>T	NM_014846	p.Asp594Tyr	0 / 1150	+	+	+
				PTBP1	19	808,702	c.1325G>A	NM_031991	p.Gly442Asp	0 / 1150	+	+	+
OKI-045-1	M	+	Unknown	*SMCO2*	12	27,623,680	c.116C>T	NM_001145010	p.Thr39Ile	0 / 1150	-	+	+
OKI-048-1	F	+	Unknown	*ZMYM4*	1	35,863,058	c.3111G>C	NM_005095	p.Lys1037Asn	0 / 1150	+	-	+
OKI-050-1	F	+	Unknown	*HIST1H3F*	6	26,250,635	c.199C>T	NM_021018	p.Pro67Ser	0 / 1150	+	+	N/A
SIZ-552	F	Unknown	+	*PAPD7*	5	6,750,594	c.C1217T	NM_006999	p.Ser363Phe	0 / 1150	+	+	+
				FSCN1	7	5,645,002	c.A1493G	NM_003088	p.Lys460Arg	0 / 1150	+	+	-
				KCNT1	9	138,671,271	c.C2870A	NM_020822	p.Phe932Leu	0 / 1150	+	+	+
				MYO1A	12	57,441,099	c.661G>A	NM_005379	p.Pro140Ser	0 / 1150	+	+	+
SIZ-664	M	Unknown	+	*TXNDC15*	5	134,210,146	c.C687C>T	NM_024715	p.Pro10Leu	0 / 1150	-	-	-
SIZ-731	F	Unknown	+	*KLHL32*	6	97,562,151	c.1120C>T	NM_052904	p.Arg374Cys	0 / 1150	+	+	+
SIZ-897	M	-	+	*POLR2H*	3	184,081,303	c.23A>G	NM_006232	p.Asp8Gly	0 / 1150	+	+	+
				TECPR2	14	102,964,014	c.4039G>A	NM_014844	p.Asp1347Asn	0 / 1150	+	+	+
				ADCY7	16	50,338,442	c.1540C>T	NM_001114	p.Pro514Ser	0 / 1150	-	-	-
SIZ-1008	M	Unknown	+	*MKL2*	16	14,234,491	c.28G>A	NM_014048	p.Gln10Lys	0 / 1150	-	+	-
SIZ-1015	M	Unknown	-	*KLHL15*	X	24,024,542	c.269A>G	NM_030624	p.Tyr90Cys (Hemi.)	0 / 869	+	+	+

Supplementary Table 4. *De novo* mutations identified by whole exome sequencing.

Mutations not found in control individuals were listed.

Truncation mutations and corresponding gene names are displayed in bold. All *de novo* mutations are heterozygous except for the p.Tyr90Cys hemizygous (Hemi) mutation of *KLHL15* in SIZ-1015. *IQSEC2*, *DDX3X* and *KLHL15* genes are located on chromosome X.

The 575 in-house controls consist of 281 males and 294 females.

Trigonocephaly / Epilepsy +: present, –: absent.

[1] Mutation taster: http://mutationtaster.org/

[2] PolyPhen2: http://genetics.bwh.harvard.edu/pph2/

Patient ID	Gender	Trigonocephaly	Epilepsy	Gene	Chr.	Position (hg19)	Nucleotide change	RefSeq gene ID	Amino acid change	Mutant allele count in In-house controls	Mutation taster[1]	PolyPhen2[2]	SIFT[3]
OKI-005-1	M	+	-	*PJA1*	X	68,381,956	c.1126C>T	NM_145119	p.Arg376Cys	0 / 869	-	-	+
OKI-005-2	M	+	-										
OKI-020-1	M	+	-										
OKI-020-4	M	+	-										
SIZ-897	M	-	+										
OKI-019-1	M	+	Unknown	*ELK1*	X	47,500,873	c.-33T>C	NM_005229	5' UTR	0 / 869	N/A	N/A	N/A
				PHKA1	X	71,825,181	**c.2755C>T**	NM_002637	**p.Arg919X**	0 / 869	+	N/A	N/A
OKI-028-1	M	+	Unknown	***OGT***	X	70,781,755	**c.1947+5A>C**	NM_181673	**5' splice site**	0 / 869	-	N/A	N/A
OKI-040-1	M	+	Unknown	***CYSLTR1***	X	77,529,182_77,529,183	**c.61_62delTT**	NM_006639	**p.Phe21fs**	0 / 869	+	N/A	N/A
OKA-02-26	M	Unknown	+	*AVPR2*	X	153,171,648	c.688C>T	NM_000054	p.Arg230Trp	0 / 869	+	+	+
				FLNA	X	153,593,562	c.1633A>G	NM_001110556	p.Met545Val	0 / 869	-	-	-
OKI-005-2	M	+	-			153,586,626	c.4696G>A	NM_001456	p.Val1566Met	0 / 869	+	+	+
				GPR112	X	135,428,872	c.3007C>T	NM_153834	p.His1003Tyr	0 / 869	-	+	+
				CHM	X	85,156,130	c.1308C>A	NM_000390	p.Asp436Glu	0 / 869	+	+	+
OKI-005-1	M	+	-										
OKA-03-43	M	Unknown	+	*DIAPH2*	X	96,638,933	c.3035G>T	NM_006729	p.Arg1012Ile	0 / 869	+	+	-
OKA-219	M	Unknown	+	*BEND2*	X	18,198,704	c.1082A>C	NM_001184767	p.Asn361Thr	0 / 869	-	+	+
				EBP	X	48,382,314	c.155G>A	NM_006579	p.Arg52His	0 / 869	-	+	+
OKI-003-1	M	+	Unknown	*STARD8*	X	67,943,523	c.2855C>G	NM_001142503	p.Pro952Arg	0 / 869	-	+	+
OKI-004-1	M	+	Unknown	*PHF8*	X	53,966,627	c.2972A>G	NM_015107	p.Asn991Ser	0 / 869	-	-	-
				GPRASP1	X	101,909,662	c.821G>A	NM_001099411	p.Gly274Glu	0 / 869	-	-	+
OKI-007-1	M	+	Unknown	*POLA1*	X	24,742,533	c.1264G>A	NM_016937	p.Ala422Thr	0 / 869	+	+	-
				ZNF75D	X	134,421,179	c.1423C>A	NM_007131	p.Gln475Lys	0 / 869	-	-	-
OKI-011-2	M	+	Unknown	*FAM47C*	X	37,026,557	c.74C>G	NM_001013736	p.Pro25Arg	0 / 869	-	-	-
OKI-013-1	M	+	Unknown	*RNF128*	X	105,937,369	c.137T>C	NM_024539	p.Ile46Thr	0 / 869	-	-	-
				LAGE3	X	153,707,199	c.56G>A	NM_006014	p.Gly19Asp	0 / 869	-	-	-
OKI-014-1	M	+	Unknown	*IDH3G*	X	153,053,009	c.584C>T	NM_004135	p.Ala195Val	0 / 869	+	-	-
OKI-017-1	M	+	Unknown	*CXorf40A*	X	148,627,336	c.160C>T	NM_178124	p.Arg54Trp	0 / 869	-	-	-
OKI-020-1	M	+	-	*SLC25A14*	X	129,506,912	c.966G>T	NM_001282195	p.Arg322Ser	0 / 869	+	-	-
OKI-024-1	M	+	Unknown	*POF1B*	X	84,537,246	c.1727C>A	NM_024921	p.Thr576Asn	0 / 869	+	+	+
OKI-025-1	M	+	Unknown	*GABRQ*	X	151,806,703	c.47T>A	NM_018558	p.Ile16Asn	0 / 869	+	+	N/A
OKI-026-1	M	+	Unknown	*ITIH6*	X	54,785,282	c.1225G>A	NM_198510	p.Val409Met	0 / 869	-	-	-
OKI-027-1	M	+	Unknown	*ZNF280C*	X	129,349,302	c.1844G>A	NM_017666	p.Arg615His	0 / 869	-	-	-
OKI-032-1	M	+	Unknown	*CACNA1F*	X	49,076,226_49,076,227	c.2441_2442insAGAAGA	NM_005183	p.814_815insGluGlu	0 / 869	-	N/A	N/A
OKI-034-1	M	+	Unknown	*LHFPL1*	X	111,874,691	c.620C>T	NM_178175	p.Thr207Ile	0 / 869	+	+	+
OKI-034-4	M	+	Unknown	*PCYT1B*	X	24,580,204	c.991T>C	NM_001163265	p.X331Gln	0 / 869	-	N/A	N/A
				WDR45	X	48,932,923	c.845A>G	NM_001029896	p.Lys282Arg	0 / 869	+	+	+
OKI-039-1	M	+	Unknown	*GYG2*	X	2,761,304	c.151G>A	NM_003918	p.Gly51Ser	0 / 869	+	+	+
OKI-041-1	M	+	Unknown	*TNMD*	X	99,840,036	c.21G>T	NM_022144	p.Glu7Asp	0 / 869	-	-	-
				ARMCX1	X	100,808,097	c.184G>A	NM_016608	p.Ala62Thr	0 / 869	-	-	-
OKI-042-1	M	+	-	*CNKSR2*	X	21,667,039	c.2693G>A	NM_001168647	p.Arg898His	0 / 869	+	+	-
OKI-044-1	M	+	Unknown	*NKAP*	X	119,070,289_119,070,294	c.639_644delCGACAG	NM_024528	p.Asp214_Ser215del	0 / 869	+	N/A	N/A
OKI-045-1	M	+	Unknown	*GNL3L*	X	54,581,081	c.1402A>G	NM_001184819	p.Thr468Ala	0 / 869	-	-	-
				MTMR8	X	63,576,094	c.272A>G	NM_017677	p.His91Arg	0 / 869	-	+	-
SIZ-330	M	Unknown	+	*PHEX*	X	22,117,137	c.947G>T	NM_000444	p.Gly316Val	0 / 869	+	-	-
				DGAT2L6	X	69,424,819	c.877A>G	NM_198512	p.Ile293Val	0 / 869	-	-	-
				PLXNA3	X	153,689,644	c.800C>T	NM_017514	p.Ala267Val	0 / 869	-	-	-
SIZ-335	M	Unknown	+	*ARHGEF9*	X	62,893,959_62,893,960	c.882_883insATT	NM_015185	p.Ile294_Asp295insIle	0 / 869	+	N/A	N/A
				ZDHHC9	X	128,962,978	c.307G>A	NM_016032	p.Ala103Thr	0 / 869	+	-	-
SIZ-439	M	Unknown	+	*TRMT2B*	X	100,275,562	c.1082C>T	NM_024917	p.Ser361Phe	0 / 869	+	+	+
				ZFY	Y	2,829,362	c.309T>G	NM_003411	p.Asp103Glu	0 / 281	N/A	-	-
SIZ-664	M	Unknown	+	*SYTL5*	X	37,981,362_37,981,364	c.1801_1803delAAG	NM_001163334	p.Lys601del	0 / 869	+	N/A	N/A
				TAF1	X	70,609,508	c.2834A>T	NM_004606	p.Asp945Val	0 / 869	+	+	+
				KDM5D	Y	21,867,964	c.4537C>G	NM_004653	p.His1513Asp	0 / 281	-	-	-
SIZ-894	M	Unknown	+	*MAP3K15*	X	19,379,697	c.3694C>G	NM_001001671	p.Pro1232Ala	0 / 869	-	+	-
SIZ-897	M	-	+	*CDX4*	X	72,667,322	c.233C>T	NM_005193	p.Pro78Leu	0 / 869	+	-	-
SIZ-1007	M	Unknown	-	*AKAP4*	X	49,957,601	c.1763G>A	NM_003886	p.Gly588Glu	0 / 869	-	+	-
SIZ-1068	M	Unknown	+	*PCDH19*	X	99,551,604	c.2977G>A	NM_001105243	p.Asp993Asn	0 / 869	+	+	-
SIZ-1076	M	Unknown	+	*SLC35A2*	X	48,761,896	c.700G>C	NM_001032289	p.Gly234Arg	0 / 869	-	+	+
				ODZ1	X	123,699,317	c.2171G>A	NM_001163278	p.Arg724His	0 / 869	+	+	+
SIZ-1101	M	Unknown	+	*ARSF*	X	3,030,331	c.1507C>A	NM_001201538	p.Pro503Thr	0 / 869	+	+	+

Supplementary Table 5. Hemizygous mutations identified by whole exome sequencing.

Mutations not found in control individuals were listed.

Truncation mutations and corresponding gene names are displayed in bold. These variants in patients have been validated by Sanger sequencing. *ZFY* and *KDM5D* genes are located on chromosome Y.

The 575 in-house controls consist of 281 males and 294 females.

Trigonocephaly / Epilepsy +: present, –: absent.

[1] Mutation taster: http://mutationtaster.org/

[2] PolyPhen2: http://genetics.bwh.harvard.edu/pph2/

[3] SIFT: http://sift.jcvi.org

Predictive outcome: +: damaging / disease causing, –: polymorphism / benign, N/A: not available, RefSeq: Reference Sequence.

Patient ID	Gender	Trigonocephaly	Epilepsy	Gene	Chr.	Position (hg19)	Nucleotide change	RefSeq gene ID	Amino acid change	Mutant allele count in In-house controls	Mutation taster[1]	PolyPhen2[2]	SIFT[3]
SIZ-335	M	Unknown	+	***LILRA3***	19	54,803,245	**c.432delG**	NM_006865	**p.Gln144fs**	0 / 1150	+	N/A	N/A
SIZ-672	F	Unknown	+										
SIZ-978	F	Unknown	+	***ZNF749***	19	57,956,746_57,956,747	**c.2230_2231insA**	NM_001023561	**p.Thr744fs**	0 / 1150	+	N/A	N/A
OKA-03-33	F	Unknown	+	*GOLGA6B*	15	72,954,623	c.878T>C	NM_018652	p.Val293Ala	0 / 1150	-	-	-
OKI-007-1	M	+	Unknown	*CCDC8*	19	46,914,479	c.1589A>C	NM_032040	p.Glu530Ala	0 / 1150	-	+	-
OKI-010-1	M	+	Unknown	*TFIP11*	22	26,895,192	c.1207A>T	NM_012143	p.Ile403Phe	0 / 1150	+	-	-
OKI-020-1	M	+	-	*AHNAK2*	14	105416832_105416839	c.4949^4956.CGGTGA CT>GCATGACA	NM_138420	p.AlaVal1650_1651GlyMet	0 / 1150	N/A	N/A	N/A
OKI-034-1	M	+	Unknown	*ANKRD36*	2	97,864,326	c.2686T>A	NM_001164315	p.Ser896Thr	0 / 1150	-	+	+
OKI-034-4	M	+	Unknown										
SIZ-396	M	Unknown	+	*HEATR5B*	2	37,241,024	c.4244C>T	NM_019024	p.Thr1415Met	0 / 1150	+	-	-
				ARID2	12	46,244,028	c.2122C>T	NM_152641	p.Pro708Ser	0 / 1150	+	+	+
SIZ-503	F	Unknown	+	*LAMA5*	20	60,889,639	c.8339G>A	NM_005560	p.Arg2780His	0 / 1150	-	-	-
SIZ-782	M	Unknown	+	*PALMD*	1	100,154,649	c.833C>A	NM_017734	p.Thr278Ans	0 / 1150	-	+	+
				BDP1	5	70,810,793	c.4493C>T	NM_018429	p.Ser1498Leu	0 / 1150	-	-	-
				CCDC73	11	32,635,270	c.2594A>G	NM_001008391	p.Glu865Gly	0 / 1150	-	-	+
				TRPV4	12	110,230,485	c.1475C>T	NM_001177433	p.Thr492Met	0 / 1150	+	+	-
SIZ-990	F	Unknown	+	*CTAGE6P*	7	143,452,534	c.2218A>G	NM_178561	p.Arg740Gly	0 / 1150	N/A	-	N/A
SIZ-1068	M	Unknown	+	*IL22*	12	68,647,114	c.115C>T	NM_020525	p.His39Tyr	0 / 1150	-	-	+

Supplementary Table 6. Homozygous mutations identified by whole exome sequencing.

Mutations not found in control individuals were listed.

Truncation mutations and corresponding gene names are displayed in bold. The 575 in-house controls consist of 281 males and 294 females.

Trigonocephaly or Epilepsy +: present, –: absent.

[1] Mutation taster: http://mutationtaster.org/

[2] PolyPhen2: http://genetics.bwh.harvard.edu/pph2/

[3] SIFT: http://sift.jcvi.org

Predictive outcome: +: damaging / disease causing, –: polymorphism / benign, N/A: not available, RefSeq: Reference Sequence.

Patient ID	Gender	Trigonocephaly	Epilepsy	Gene	Chr.	Position (hg19)	Nucleotide change	RefSeq gene ID	Amino acid change	Mutant allele count in In-house controls	Mutation taster[1]	PolyPhen2[2]	SIFT[3]
OKI-027-1	M	+	Unknown	***ANKS1B***	12	99,201,638	c.140T>C	NM_001204065	p.Ile47Thr	0 / 1150	-	+	-
						99,201,699	**c.85-6C>T**		**3' splice site**		+	N/A	N/A
OKI-030-1	F	+	Unknown	***STAB2***	12	104,152,924	c.7121G>A	NM_017564	p.Arg2374Gln	0 / 1150	+	+	-
						104,081,944	**c.2998-2A>T**		**3' splice site**		N/A	N/A	N/A
OKI-044-1	M	+	Unknown	***RTTN***	18	67,759,911	**c.4032+1G>A**	NM_173630	**5' splice site**	0 / 1150	+	N/A	N/A
						67,836,272	c.1508C>T		p.Thr503Ile		-	+	+
OKI-004-1	M	+	Unknown			179,542,549	c.30358C>A	NM_133378	p.Leu10120Ile	0 / 1150	-	-	-
						179,650,441	c.2399A>G		p.Glu800Gly		-	+	+
SIZ-503	F	Unknown	+	*TTN*	2	179,459,184	c.31217T>C	NM_133432	p.Val10406Ala	0 / 1150	-	-	-
						179,597,743	c.12428G>C	NM_133378	p.Arg4143Thr		-	-	-
SIZ-1034	F	Unknown	+			179,440,595	c.G43069C	NM_003319	p.Gly14357Arg	0 / 1150	+	+	+
						179,614,820	c.G12307A	NM_133379	p.Val4103Ile		+	+	-
OKI-001-1	M	+	Unknown	*SYNE1*	6	152,443,792	c.26029G>A	NM_033071	p.Asp8677Asn	0 / 1150	-	+	-
						152,708,479	c.8236G>A		p.Val2746Ile		-	-	+
OKI-007-1	M	+	Unknown	*ONECUT1*	15	53,049,957	c.1193G>A	NM_004498	p.Arg398Gln	0 / 1150	+	+	+
						53,081,690_53,081,691	c.389_390insCCA		p.130_131insPro		-	N/A	N/A
OKI-008-1	F	+	Unknown	*TCHH*	1	152,081,778	c.3915G>C	NM_007113	p.Lys1305Asn	0 / 1150	-	+	+
						152,082,953	c.2740C>G		p.Gln914Glu		-	-	+
OKI-012-1	M	+	Unknown	*RIN1*	11	66,103,540	c.176G>A	NM_004292	p.Arg59Gln	0 / 1150	+	+	-
						66,102,117	c.1153G>T		p.Asp385Tyr		+	+	+
OKI-016-1	M	+	Unknown	*GPR179*	17	36,483,012	c.6440G>A	NM_001004334	p.Arg2147Lys	0 / 1150	-	-	-
						36,486,426	c.3026A>G		p.Gln1009Arg		-	+	+
OKI-017-1	M	+	Unknown	*FAT4*	4	126,412,615	c.14638G>C	NM_024582	p.Asp4880His	0 / 1150	+	+	-
						126,238,669	c.1103A>G		p.Asp368Gly		+	+	-
OKI-019-1	M	+	Unknown	*IGF1R*	15	99,434,827	c.914A>T	NM_000875	p.Gln305Leu	0 / 1150	-	-	-
						99,486,277	c.3583G>A		p.Val1195Ile		+	+	+
OKI-032-1	M	+	Unknown	*PTPRM*	18	7,955,360	c.1080G>T	NM_001105244	p.Glu360Asp	0 / 1150	+	-	-
						7,888,269	c.362A>G		p.Asn121Ser		+	+	+
SIZ-552	F	Unknown	+	*LGI3*	8	22,006,196	c.1124G>A	NM_139278	p.Arg375His	0 / 1150	+	+	+
						22,006,344	c.976G>A		p.Val326Met		+	+	+
OKA-218	F	Unknown	-	*PCDHB7*	5	140,552,968	c.552T>G	NM_018940	p.Asp184Glu	0 / 1150	-	-	-
						140,554,715	c.2299C>G		p.Pro767Ala		+	+	+
OKA-224	F	Unknown	+	*CAPN2*	1	223,900,347	c.5C>T	NM_001748	p.Ala2Val	0 / 1150	+	+	+
						223,959,575	c.1734C>G	NM_001146068	p.Ile578Met		+	-	-

Supplementary Table 7. Compound heterozygous mutations identified by whole exome sequencing.

Mutations not found in control individuals were listed.

Truncation mutations and corresponding gene names are displayed in bold. These variants in patients and parents have been validated by Sanger sequencing.

The 575 in-house controls consist of 281 males and 294 females.

Trigonocephaly / Epilepsy +: present, –: absent.

[1] Mutation taster: http://mutationtaster.org/

[2] PolyPhen2: http://genetics.bwh.harvard.edu/pph2/

[3] SIFT: http://sift.jcvi.org

Predictive outcome: +: damaging / disease causing, –: polymorphism / benign, N/A: not available, RefSeq: Reference Sequence.

Patient ID		SIZ-978	OKI-005-1	OKI-005-2	OKI-020-4	OKI-020-1	SIZ-897	OKI-061-1	NAG-NP167	OKI-011-1	NAG-NP089	NAG-NP149	SIZ-894	RUM062
Gene		***PJA1***									***MSX2***			
Chromosome		X									5			
Position (hg19)		68,382,459	68,381,956							68,381,625	174,151,736	174,151,837	174,156,298_174,156,299	174,156,476
Nucleotide change		c.623C>T	c.1126C>T							c.1457C>A	c.74G>T	c.175C>T	c.516_517insG	c.694G>A
RefSeq gene ID		NM_145119									NM_002449			
Mutation		p.Ser208Phe	**p.Arg376Cys (p.R376C)**							p.Pro486His	p.Gly25Val	p.Pro59Ser	**p.Ala173fs (p.A173fs)**	p.Ala232Thr
Mutant allele count in	cases	1 / 711	7 / 711							1 / 711	1 / 1116	1 / 1116	1 / 1116	1 / 1116
	controls (WES)	0 / 869	0 / 869							0 / 869	0 / 1150	0 / 1150	0 / 1150	0 / 1150
	controls (targeted seq)	0 / 441	0 / 441							0 / 441	0 / 622	0 / 622	0 / 622	0 / 622
	gnomAD[1]	NR	14 / 204,624							NR	NR	NR	NR	3 / 251,158
Zygosity		Hetero.	Hemi.	Hemi.	Hemi.	Hemi.	Hemi.	Hemi.	Hemi.	Hetero.	Hetero.	Hetero.	Hetero.	Hetero.
Gender		female	male	male	male	male	male	male	male	female	female	female	male	male
Craniofacial abnormality		unknown	**Tri**	**Tri**	**Tri**	**Tri**	–	**Tri**	unknown	**Tri**	unkown	unkown	C.M.	unknown
Epilepsy		SGE	-	-	-	-	PE	-	-	–	unkown	-	PE	-
Autistic traits		-	+	+	-	+	+	+	+	+	+	+	+	+
Delay in speech acquisition		+	+	+	+	+	+	+	+	+	unkown	+	+	+
Intellectual disability		+	+	+	+	+	+	+	+	+	unkown	-	very mild	+
Motor developmental delay		+	-	+	-	-	-	-	unknown	-	unkown	+	+	-
Hyperkinesia		-	+	+	+	+	+	+	+	-	unkown	-	-	+
Self-injurious behavior		-	+	+	-	-	unknown	+	+	-	unkown	-	-	-
Panic disorders		-	+	-	-	+	unknown	-	-	+	unkown	-	-	-
Sleep disorders		-	+	-	+	+	unknown	+	+	-	unkown	+	-	-
Note		Hemizygous mutation found in father without symptoms.								A young brother with NDD (OKI-011-2) does not have this mutation. The mutation was found in father without symptoms.	Parents samples were not available.	Parents samples were not available.	***de novo* mutation.**	This heterozygous mutation was also found in mother without symptoms.

Supplementary Table 8. *PJA1* and *MSX2* non-synonymous mutations and phenotypic features in individuals with the mutations.
Controls (886 individuals in total) consist of 575 in-house controls for whole exome sequencing (WES) (281 males and 294 females) and 311 additional independent controls for targeted sequencing (181 males and 130 females).
Tri: Trigonocephaly, C.M.: Cranial Meningocele, Hemi.: Hemizygous, Hetero.: Heterozygous, NR; not registered, +: present, –: absent, PE: Partial Epilepsy, SGE: Symptomatic Generalized Epilepsy, RefSeq: Reference Sequence.
[1]gnomAD: https://gnomad.broadinstitute.org

Population	East Asian	South Asian	Latino	European (non-Finnish)	European (Finnish)	African	Ashkenazi Jewish	Other	Total
Mutant allele count	5	3	1	5	0	0	0	0	14
Total allele number	14,828	19,066	28,039	92,352	18,569	18,794	7,656	5,320	204,624
Number of hemizygous	1	2	0	1	0	0	0	0	4
Allele frequency	3.37E-04	1.57E-04	3.57E-05	5.41E-05	0	0	0	0	6.84E-05

Supplementary Table 9. Allele frequency of PJA1 p.Arg376Cys mutation in various populations.
Allele frequency of R376C is higher in East and South Asian populations than in other ethnic backgrounds according to gnomAD database.
